# Correction: Safety verification for polysorbate 20, pharmaceutical excipient for intramuscular administration, in Sprague-Dawley rats and New Zealand White rabbits

**DOI:** 10.1371/journal.pone.0267238

**Published:** 2022-04-13

**Authors:** Junhyung Kim, Seongsung Kwak, Mi-Sun Park, Chang-Hoon Rhee, Gi-Hyeok Yang, Jangmi Lee, Woo-Chan Son, Won-ho Kang

The ORCID iD is missing for the eighth author, Won-ho Kang. The author’s ORCID iD is: 0000-0003-0184-1613 (https://orcid.org/0000-0003-0184-1613).

There is an error in affiliation 4 for author Woo-Chan Son. The correct affiliation 4 is: Department of Pathology, Asan Medical Center, University of Ulsan College of Medicine, Songpa-gu, Seoul, Korea.
