# Supplementary material for: Safety verification for polysorbate 20, pharmaceutical excipient for intramuscular administration, in Sprague-Dawley rats and New Zealand White rabbits
Source: PLoS One. 2021 Aug 27;16(8):e0256869. doi: 10.1371/journal.pone.0256869 (PMC8396741; doi:10.1371/journal.pone.0256869)
Supplement: Attachment — Submitted filename: Review PLONE.pdf [file pone.0256869.s005.pdf]

PLOS ONE

Safety verification for polysorbate 20, pharmaceutical excipient of newly developed Botulinum toxin serotype A products, in Sprague-Dawley rats and New Zealand White rabbits

--Manuscript Draft--

**Manuscript Number:**	PONE-D-21-03019
**Article Type:**	Research Article
**Full Title:**	Safety verification for polysorbate 20, pharmaceutical excipient of newly developed Botulinum toxin serotype A products, in Sprague-Dawley rats and New Zealand White rabbits
**Short Title:**	In vivo safety evaluation of polysorbate 20
**Corresponding Author:**	Won-ho Kang<br>MedyTox Inc.<br>Suwon-si, KOREA, REPUBLIC OF
**Keywords:**	Botulinum toxin type A products; Pharmaceutical excipient; Polysorbate 20; Human serum albumin
**Abstract:**	Human serum albumin (HSA) has been widely used as a pharmaceutical excipient in Botulinum toxin serotype A (BoNT/A) products that are indicated for use in therapeutics and cosmetics. However, HSA as a human-derived material has some concerns, such as the potential risk of transmission of infectious agents, an insufficient supply, and difficulty in maintaining a certain quality. For those reasons, newly developed BoNT/A products (CORETOX ® , Medytox Ltd., Republic of Korea) contained polysorbate 20, a non-human-derived excipient, to replace the HSA. However, most safety studies of polysorbate 20 have been conducted with non-invasive routes of administration, and thus there are few studies on the safety of polysorbate 20 when administered intramuscularly. To secure the in vivo safety profile of polysorbate 20, a four-week repeated intramuscular dose toxicity study (0.02, 0.1, and 0.4 mg/kg, one injection every two weeks for a total of three injections) was conducted in 66 Sprague-Dawley (SD) rats. Then, an intradermal irritation study was further conducted with 18 New Zealand White (NZW) rabbits. The toxicological evaluation of HSA (0.06 and 0.12 mg/kg) was also carried out as a comparative substance. Systemic and local toxicities were not observed in any of the SD rats or NZW rabbits based on clinical signs, body weight, hematology, clinical chemistry, macroscopic findings on necropsy, histopathology of the injection site, and allergic reactions. The current study suggested that intramuscular administration of polysorbate 20 was considered to be safe at a level similar to that of HSA, which has an in vivo safety profile accumulated over the years. This provided the basis for the in vivo safety profile of polysorbate 20 administered intramuscularly and the scientific reliability of the use of polysorbate 20 as an alternative to HSA, which is used as an excipient for various pharmaceuticals in terms of its safety.
**Order of Authors:**	Junhyung Kim
	Seongsung Kwak
	Mi-Sun Park
	Chang-Hoon Rhee
	Gi-Hyeok Yang
	Woo-Chan Son
	Won-ho Kang
**Additional Information:**	
**Question**	**Response**
**Financial Disclosure**	This study was supported by the Technology Innovation Program, establishment of risk management platform with aim of reduce attrition of new drugs and its service [grant

Enter a financial disclosure statement that describes the sources of funding for the work included in this submission. Review the submission guidelines for detailed requirements. View published research articles from *PLOS ONE* for specific examples.<br><br>This statement is required for submission and **will appear in the published article** if the submission is accepted. Please make sure it is accurate.<br><br>**Unfunded studies**<br>Enter: *The author(s) received no specific funding for this work.*<br><br>**Funded studies**<br>Enter a statement with the following details:<br>• Initials of the authors who received each award<br>• Grant numbers awarded to each author<br>• The full name of each funder<br>• URL of each funder website<br>• Did the sponsors or funders play any role in the study design, data collection and analysis, decision to publish, or preparation of the manuscript?<br>• **NO** - Include this sentence at the end of your statement: *The funders had no role in study design, data collection and analysis, decision to publish, or preparation of the manuscript.*<br>• **YES** - Specify the role(s) played.<br><br>* typeset	number: 10067737, 2016], funded by the Ministry of Trade, Industry & Energy in Republic of Korea (WCS).<br>The funders had no role in study design, data collection and analysis, decision to publish, or preparation of the manuscript.
**Competing Interests**<br><br>Use the instructions below to enter a competing interest statement for this submission. On behalf of all authors, disclose any competing interests that could be perceived to bias this work—acknowledging all financial support and any other relevant financial or non-financial competing interests.<br><br>This statement is required for submission and **will appear in the published article** if the submission is accepted. Please make	The authors have declared that no competing interests exist.

sure it is accurate and that any funding sources listed in your Funding Information later in the submission form are also declared in your Financial Disclosure statement.<br><br>View published research articles from *PLOS ONE* for specific examples.<br><br>**NO authors have competing interests**<br><br>Enter: *The authors have declared that no competing interests exist.*<br><br>**Authors with competing interests**<br><br>Enter competing interest details beginning with this statement:<br><br>*I have read the journal's policy and the authors of this manuscript have the following competing interests: [insert competing interests here]*<br><br>* typeset	
**Ethics Statement**<br><br>Enter an ethics statement for this submission. This statement is required if the study involved:<br><br>• Human participants<br>• Human specimens or tissue<br>• Vertebrate animals or cephalopods<br>• Vertebrate embryos or tissues<br>• Field research<br><br>Write "N/A" if the submission does not require an ethics statement.<br><br>General guidance is provided below. Consult the submission guidelines for detailed instructions. **Make sure that all information entered here is included in the Methods section of the manuscript.**	All studies complied with the Animal Protection Act (No. 13023) and Laboratory animal Act (No. 15944). The four-week repeated intramuscular dose toxicity study was reviewed and approved by the Institutional Animal Care and Use Committee of Medytox, Inc. (A-2019-009). The intradermal irritation study was reviewed and approved by the Institutional Animal Care and Use Committee of Korea Testing & Research Institute (KTR, IAC2019-1500).<br>All Sprague-Dawley rats were euthanized by exsanguination from the posterior vena cava and abdominal aorta under isoflurane anesthesia, while all New Zealand White rabbits were euthanized by intravenous injection of T-61 solution.

Powered by Editorial Manager® and ProduXion Manager® from Aries Systems Corporation

**Format for specific study types**<br><br>**Human Subject Research (involving human participants and/or tissue)**<br>• Give the name of the institutional review board or ethics committee that approved the study<br>• Include the approval number and/or a statement indicating approval of this research<br>• Indicate the form of consent obtained (written/oral) or the reason that consent was not obtained (e.g. the data were analyzed anonymously)<br><br>**Animal Research (involving vertebrate animals, embryos or tissues)**<br>• Provide the name of the Institutional Animal Care and Use Committee (IACUC) or other relevant ethics board that reviewed the study protocol, and indicate whether they approved this research or granted a formal waiver of ethical approval<br>• Include an approval number if one was obtained<br>• If the study involved *non-human primates*, add *additional details* about animal welfare and steps taken to ameliorate suffering<br>• If anesthesia, euthanasia, or any kind of animal sacrifice is part of the study, include briefly which substances and/or methods were applied<br><br>**Field Research**<br><br>Include the following details if this study involves the collection of plant, animal, or other materials from a natural setting:<br>• Field permit number<br>• Name of the institution or relevant body that granted permission	
**Data Availability**<br><br>Authors are required to make all data underlying the findings described fully available, without restriction, and from the time of publication. PLOS allows rare exceptions to address legal and ethical concerns. See the PLOS Data Policy and FAQ for detailed information.	Yes - all data are fully available without restriction

A Data Availability Statement describing where the data can be found is required at submission. Your answers to this question constitute the Data Availability Statement and **will be published in the article**, if accepted.<br><br>**Important:** Stating 'data available on request from the author' is not sufficient. If your data are only available upon request, select 'No' for the first question and explain your exceptional situation in the text box.<br><br>Do the authors confirm that all data underlying the findings described in their manuscript are fully available without restriction?	
**Describe where the data may be found in full sentences. If you are copying our sample text, replace any instances of XXX with the appropriate details.**<br><br>• If the data are **held or will be held in a public repository**, include URLs, accession numbers or DOIs. If this information will only be available after acceptance, indicate this by ticking the box below. For example: *All XXX files are available from the XXX database (accession number(s) XXX, XXX.).*<br>• If the data are all contained **within the manuscript and/or Supporting Information files**, enter the following: *All relevant data are within the manuscript and its Supporting Information files.*<br>• If neither of these applies but you are able to provide **details of access elsewhere**, with or without limitations, please do so. For example:<br><br>*Data cannot be shared publicly because of [XXX]. Data are available from the XXX Institutional Data Access / Ethics Committee (contact via XXX) for researchers who meet the criteria for access to confidential data.*<br><br>*The data underlying the results presented in the study are available from (include the name of the third party*	All relevant data are within the manuscript and its Supporting Information files.

and contact information or URL).<br>• This text is appropriate if the data are owned by a third party and authors do not have permission to share the data.<br><br>* typeset	
Additional data availability information:	

Manuscript
Click here to access/download;Manuscript;Manuscript.doc

Safety verification for polysorbate 20, pharmaceutical excipient of newly developed Botulinum toxin serotype A products, in Sprague-Dawley rats and New Zealand White rabbits

Short title: *In vivo* safety evaluation of polysorbate 20

Junhyung Kim[1⁋], Seongsung Kwak[1, 2⁋], Mi-Sun Park[1], Chang-Hoon Rhee[3], Gi-Hyeok Yang[1, 3], Woo-Chan Son[2&*], and Won-ho Kang[1&*]

[1]Gwanggyo R&D Center, Medytox Inc., Suwon-si, Gyeonggi-do, Republic of Korea

[2]Department of Pathology, Asan Medical Center, Songpa-gu, Seoul, Republic of Korea

[3]Osong R&D Center, Medytox Inc., Cheongju-si, Chungcheongbuk-do, Republic of Korea

*Corresponding author

E-mail: whkang@medytox.com (WhK)

E-mail: wcson32@hanmail.net (WCS)

[⁋] These authors contributed equally to this work as first authors

[&]These authors were contributed equally to this work as corresponding authors

Abstract

Human serum albumin (HSA) has been widely used as a pharmaceutical excipient in Botulinum toxin serotype A (BoNT/A) products that are indicated for use in therapeutics and cosmetics. However, HSA as a human-derived material has some concerns, such as the potential risk of transmission of infectious agents, an insufficient supply, and difficulty in maintaining a certain quality. For those reasons, newly developed BoNT/A products (CORETOX®, Medytox Ltd., Republic of Korea) contained polysorbate 20, a non-human-derived excipient, to replace the HSA. However, most safety studies of polysorbate 20 have been conducted with non-invasive routes of administration, and thus there are few studies on the safety of polysorbate 20 when administered intramuscularly. To secure the *in vivo* safety profile of polysorbate 20, a four-week repeated intramuscular dose toxicity study (0.02, 0.1, and 0.4 mg/kg, one injection every two weeks for a total of three injections) was conducted in 66 Sprague-Dawley (SD) rats. Then, an intradermal irritation study was further conducted with 18 New Zealand White (NZW) rabbits. The toxicological evaluation of HSA (0.06 and 0.12 mg/kg) was also carried out as a comparative substance. Systemic and local toxicities were not observed in any of the SD rats or NZW rabbits based on clinical signs, body weight, hematology, clinical chemistry, macroscopic findings on necropsy, histopathology of the injection site, and allergic reactions. The current study suggested that intramuscular administration of polysorbate 20 was considered to be safe at a level similar to that of HSA, which has an *in vivo* safety profile accumulated over the years. This provided the basis for the *in vivo* safety profile of polysorbate 20 administered intramuscularly and the scientific reliability of the use of polysorbate 20 as an alternative to HSA, which is used as an excipient for various pharmaceuticals in terms of its safety.

Introduction

Botulinum toxin serotype A (BoNT/A) is one of the neurotoxic proteins produced by bacteria of the genus *Clostridium*, particularly *Clostridium botulinum* [1]. A BoNT/A product was first approved by the Food and Drug Administration (FDA) in 1989 for the treatment of strabismus and blepharospasm associated with dystonia [2]. Since then, it has been further approved for the treatment of various diseases, such as cervical dystonia, severe primary axillary hyperhidrosis, overactive bladder, and post-stroke spasticity [3]. In parallel, BoNT/A products were approved by the FDA in 2002 for the treatment of moderate-to-severe glabellar lines, and they began to be widely used for cosmetic purposes [4,5]. As a result, the cosmetic use of BoNT/A has increased every year, and BoNT/A injection is currently the most common cosmetic surgery in the United States (7.4 million procedures in 2018) [6].

Most BoNT/A products use human serum albumin (HSA), which has few reports of toxicity and immunogenicity in humans, as an excipient [7,8]. HSA, the most abundant protein in human plasma, has the functions of regulating the colloid osmotic pressure, binding to and transporting various molecules, antioxidant action, anticoagulant action, regulating membrane permeability, and a direct neuroprotective action [9,10]. Therefore, it is used as an excipient for various pharmaceuticals in biomedical aspects, in addition to therapeutic purposes for hemorrhage, hypovolemia, and hypoalbuminemia [9,11]. In BoNT/A products, HSA plays a role in stabilizing the neurotoxic proteins during manufacturing, transportation, and administration and in preventing the aggregation of neurotoxic proteins [12]. However, there are several concerns with HSA, although it is produced under strict regulations and has a safety profiling accumulated over the years [10,12]. Since the production of HSA mainly relies on human blood donation, the quality of HSA could differ depending on the population of the human blood donors (race or geographical distribution) [13]. Furthermore, the market demand for HSA has dramatically increased (over 500 tons), and it is one of the most widely used biopharmaceutical solutions today [13,14]. This could lead to a worldwide shortage of

HSA in the market [10]. Finally, the use of HSA has a theoretical risk of spreading blood-derived pathogens [12,15]. Currently, the transmission of viral diseases, such as hepatitis B virus, human immunodeficiency virus, and West Nile virus are well controlled through strict management of HSA, but there are still concerns about the transmission of prion-derived variant Creutzfeldt-Jakob disease [16,17].

For those reasons, Medytox, Inc. (Republic of Korea) has recently developed a complexing protein-free BoNT/A product (CORETOX®) that uses polysorbate 20, a non-human-derived excipient, to replace the HSA [18,19]. Polysorbates, a series of polyoxyethylenated sorbitan esters, are hydrophilic and nonionic surfactants that have been widely used as a food additive and stabilizer in pharmaceuticals [20,21]. Among them, polysorbate 20, called polyoxyethylene-20-sorbitan monolaurate, has 20 repeating units of polyethylene glycol. It is mainly used as a direct/indirect additive in food and emulsifiers/surfactants in cosmetics that come into direct contact with the human skin, such as shampoo, bath soap, skin cleansing products, and hair spray [21]. It has also been used as an excipient in pharmaceuticals as a stabilizer, particularly in tablet and ophthalmic solutions. Hence, most of the safety studies on polysorbate 20 have been conducted on oral toxicity and skin irritation [20]. There are few studies on the safety of polysorbate 20 administered by other routes, especially intramuscular administration, which is the route of BoNT/A injection.

The objective of this study was to verify the *in vivo* safety properties of polysorbate 20, an excipient in a newly developed BoNT/A product, when administered intramuscularly. First, a four-week repeated intramuscular dose toxicity study in Sprague-Dawley (SD) rats (one injection every two weeks for a total of three injections) was carried out to evaluate its systemic toxicity. Second, an intradermal irritation study in New Zealand White (NZW) rabbits was carried out to evaluate local toxicity and allergic reactions.

Materials and Methods

Animals and Facility

This study was reviewed and approved by the Institutional Animal Care and Use Committee of Medytox, Inc. (A-2019-009) and the Korea Testing & Research Institute (KTR, IAC2019-1500). In addition, the study design was established based on the ARRIVE guidelines [22].

In the four-week repeated intramuscular dose toxicity study performed at Medytox R&D Center (Republic of Korea), a total of 66 SD rats, consisting of 30 females and 36 males, were used (Table 1). Healthy five-week-old SD rats were purchased from Orient Bio, Inc. (Republic of Korea). Upon receipt, all animals were macroscopically examined and acclimated for 7 days to the laboratory conditions (a specific pathogen-free animal room; temperature, 23 ± 3°C; humidity, 55 ± 15%; ventilation, 15 air changes per hour; light/dark cycle, 12 hours; light intensity, 150 ~ 300 Lux; polycarbonate cage; 2 ~ 3 animals per cage).

In the intradermal irritation study performed at KTR (Republic of Korea), a total of 18 male NZW rabbits were used (Table 1). Healthy three-month-old rabbits were purchased from DooYeol Biotech (Republic of Korea). Upon receipt, all animals were macroscopically examined and acclimated for 6 days to the laboratory conditions (specific pathogen-free animal room; temperature, 20.3 ~ 21.9°C; humidity, 54.5 ~ 64.9%; ventilation, 10~ 20 air changes per hour; light/dark cycle, 12 hours; light intensity, 150 ~ 300 Lux; stainless steel cage; 1 animal per cage).

Table 1. Study design of four-week repeated intramuscular dose toxicity study in Sprague-Dawley rats and intradermal irritation study in New Zealand white rabbits

Study	Species	Group	Substance	Dose volume	Dose	Route and Frequency of administration	No. animals (Male/Female)
**Four-week repeated intramuscular dose toxicity study**	**Sprague-Dawley rats**	G1	Vehicle (0.9% saline)	0.2 mL/kg	0 mg/kg	Intramuscular injection (Gastrocnemius muscle)<br>Every two weeks (Total 3 times)	11 (6/5)
		G2	Polysorbate 20		0.02 mg/kg		11 (6/5)
		G3	Polysorbate 20		0.1 mg/kg		11 (6/5)
		G4	Polysorbate 20		0.4 mg/kg		11 (6/5)
		G5	Human serum albumin		0.06 mg/kg		11 (6/5)
		G6	Human serum albumin		0.12 mg/kg		11 (6/5)
		**Total**					**66 (36/30)**
**Intradermal irritation study**	**New Zealand White rabbits**	G1	Vehicle (0.9% saline)	0.4 mL/kg/site (Total 5 sites)	0 mg/kg/site	Intradermal injection (Dorsal back skin)<br>Once (Total 1 time)	3 (3/0)
		G2	Polysorbate 20		0.02 mg/kg/site		3 (3/0)
		G3	Polysorbate 20		0.1 mg/kg/site		3 (3/0)
		G4	Polysorbate 20		0.4 mg/kg/site		3 (3/0)
		G5	Human serum albumin		0.06 mg/kg/site		3 (3/0)
		G6	Human serum albumin		0.12 mg/kg/site		3 (3/0)
		**Total**					**18 (18/0)**

Vehicle, positive control substance, and test substance

Saline (0.9% NaCl, DAI HAN PHARM. Co., Republic of Korea) was used as the vehicle in this study conducted in SD rats and NZW rabbits. HSA (Green Cross Corp., Republic of Korea) was selected as the comparative substance for the polysorbate 20 (Merck, Germany) that was the test substance of the current study. In the four-week repeated intramuscular dose toxicity study, the dose of HSA was calculated by multiplying the animal equivalent dose calculation factor (6.2) and the content of HSA in most BoNT/A products, 0.01 and 0.02 mg/kg (0.5 and 1.0 mg/vial, 50 kg human) [7,23]. As a result, 0.06 and 0.12 mg/kg were selected as the dose of HSA. The dose of polysorbate 20 was selected based on the content of polysorbate 20 in CORETOX® (1.0 mg/vial, 60 kg human) and the human dosage of CORETOX® (0.2 ~ 3.6 vial) [18,19]. Then, the dose of polysorbate 20 was also calculated in the same manner as when determining the dose of HSA. As a result, 0.02, 0.1, and 0.4 mg/kg were selected as the dose of polysorbate 20.

The same dose of HSA and polysorbate 20 used in the four-week repeated intramuscular dose toxicity study was applied in the intradermal irritation study in NZW rabbits. Detailed information is provided in Table 1.

In vivo administration

In the four-week repeated intramuscular dose toxicity study in SD rats, all substances (saline, HSA, polysorbate 20) were administered intramuscularly. The dosing formulation was prepared on the day of administration using saline (HSA: 0.3, 0.6 mg/mL; polysorbate 20: 0.1, 0.5, 2.0 mg/mL). The dose volume was selected as 0.2 mL/kg and the individual dose volume for each animal was calculated based on the body weight of the animal on the day of administration [24]. All substances were injected once every two weeks for four weeks (a total of 3 times; days 1, 15, 29) into one site of the gastrocnemius muscle of the right hindlimb.

In the intradermal irritation study in NZW rabbits, all substances were administered via the intradermal route. The dosing formulation was prepared on the day of administration using saline (HSA: 0.15, 0.3 mg/mL; polysorbate 20: 0.05, 0.25, 1.0 mg/mL). The dose volume was selected as 0.4 mL/kg/site (total 2 mL/kg/head) and the individual dose volume for each animal was calculated based on the body weight of animal on the day of administration [24]. Before administration, the back skin of the rabbits was depilated of hair. Then, all substances were injected once into five sites (three left side and two right side) on the back skin (total 1 time; day 1).

Clinical observation and body weights measurement

In the four-week repeated intramuscular dose toxicity study in SD rats, all animals were observed for clinical signs and general condition once daily throughout the study period (days 1 ~ 29). Their body weights were measured once a week (total five times), including the day of administration and at autopsy.

In the intradermal irritation study in NZW rabbits, clinical observation was conducted once daily throughout the study period (days 1 ~ 29). Furthermore, skin reactions at the administration site, including the degree of erythema, crust formation, and edema formation, were observed once daily based on the scoring system for skin reactions of the International Organization for Standardization [25]. Body weights were measured once a week (total five times), including on the day of administration and at autopsy.

Hematology, clinical chemistry, and allergic reaction test

All animals (SD rats and NZW rabbits) were fasted overnight before the last day of the study. Then, the SD rats were anesthetized with isoflurane and blood samples were collected from their

abdominal vein. In the NZW rabbits, blood samples were collected from the marginal ear vein without anesthesia.

Approximately 1 mL of the blood sample of all animals was put into a tube containing EDTA. After that, the following parameters were analyzed in KTR using a hematological autoanalyzer (ADVIA 120, SIEMENS, Germany): white blood cell count (WBC), differential leucocyte count (neutrophils; lymphocytes; monocytes; eosinophils; basophils), red blood cell count (RBC), hemoglobin (HGB), hematocrit (HCT), RBC indices (mean corpuscular volume, MCV; mean corpuscular hemoglobin, MCH; mean corpuscular hemoglobin concentration, MCHC), reticulocyte, and platelets.

The blood samples (except those used in the hematological analysis) were centrifuged at 3,000 rpm for 10 minutes to obtain the serum. Then, the following parameters were examined in KTR using an automatic analyzer (TBA-120FR, TOSHIBA, Japan): total protein, albumin, albumin/globulin ratio, total bilirubin, total bile acid, alkaline phosphatase (ALP), aspartate aminotransferase (AST), aspartate aminotransferase (AST), alanine aminotransferase (ALT), gamma glutamyltransferase (GGT), creatinine, blood urea nitrogen (BUN), total cholesterol, triglycerides (TG), glucose, calcium, inorganic phosphorus, creatine phosphokinase, cholinesterase, sodium, potassium, and chloride. Among these, total bile acid and cholinesterase were only analyzed for the NZW rabbits.

In addition, the levels of serum IgE and histamine of all animals were measured using an enzyme-linked immunosorbent assay (ELISA) kit.

Necropsy and histopathology

After blood sampling from the SD rats and NZW rabbits, autopsies were performed on all

animals at day 29 based on the AVMA Guidelines for the Euthanasia of Animals [26]. All SD rats were euthanized by exsanguination from the posterior vena cava and abdominal aorta under isoflurane anesthesia. All NZW rabbits were euthanized by intravenous injection of T-61 solution (MSD animal health, Republic of Korea). At necropsy, their external appearance, internal organs, and injection sites were observed macroscopically using the naked eye. Then, the injection site (one site of the gastrocnemius muscle of the right hindlimb for the SD rats and the five sites of the back skin for the NZW rabbits) were collected and stored in 10% neutral buffered formalin. Slides of all preserved tissues for histopathology were prepared through general tissue processing, including dehydration and paraffin embedding. Then, all slides were stained with hematoxylin & eosin and examined microscopically at KTR.

Statistical analysis

The Kruskal-Wallis test was performed to compare toxicological indicators, including body weights, hematology level, clinical chemistry level, serum IgE level, and serum histamine levels among the groups of SD rats and NZW rabbits. In the case of body weight, since there was a difference in the mean body weight among the groups before the first dose administration, the statistical analysis was performed using the ratio of body weight at the time of the measurement to the body weight before the first administration. All statistical analyses were performed using GraphPad Prism 8.0 (GraphPad Software, Inc., San Diego, CA) and a p-value of < 0.05 was accepted to indicate statistical significance.

Results

Mortality and Clinical Signs

In the four-week repeated intramuscular dose toxicity study, all SD rats survived throughout the study period. In addition, no specific clinical signs were observed in all animals, including no symptoms at the site of administration. In the intradermal irritation study, no deaths or abnormal findings were observed in all NZW rabbits. Skin reactions, including erythema, eschar formation, and edema formation, were not observed regardless of the administered substance.

Changes in body weights

For both the SD rats and NZW rabbits, their body weight was measured once a week for a total of five times. Individual body weights of all animals by time point are presented in the S1 and S2 Tables.

In the four-week repeated intramuscular dose toxicity study, a gradual body weight gain of the SD rats was observed regardless of the administered substance (Fig 1a). Compared to the body weight before the first administration, all male SD rats had a body weight of 138.4% at day 8, 173.6% at day 15, 200.4% at day 22, and 225.0% at day 29, respectively, while all female SD rats had a body weight of 132.2% at day 8, 153.1% at day 15, 173.1% at day 22, and 187.3% at day 29, respectively. There was no significant increase or decrease of the body weight of the SD rats administered polysorbate 20 and HSA compared to the vehicle control group at any of the time points.

In the intradermal irritation study, a gradual body weight gain of the NZW rabbits was also observed regardless of the administered substance (Fig 1b). Compared to their body weight before administration, all NZW rabbits had a weight of 105.0% at day 8, 111.0% at day 15, 116.7% at day 22, and 120.8% at day 29. There was also no significant difference in body weight of the NZW rabbits administered polysorbate 20 and HSA compared to the vehicle control group at any of the time points.

Fig 1. Body weight changes in a four-week repeated intramuscular dose toxicity study in Sprague-Dawley (SD) rats and an intradermal irritation study in New Zealand White (NZW) rabbits. Body weight changes measured once a week in a) SD rats and b) NZW rabbits, for a total of five times during the study period. In SD rats, all substances were injected once every two weeks for four weeks (total three times; days 1, 15, 29) into one site of the gastrocnemius muscle of the right hindlimb. Neither male nor female rat groups showed significant differences in body weight on days 8, 15, 22, and 29 (Kruskal-Wallis test). In NZW rabbits, all substances were injected once into five sites of the back skin (total one time; day 1). There was no significant difference in body weight on days 8, 15, 22, and 29 in all groups (Kruskal-Wallis test).

Findings of the level of hematology, clinical chemistry, serum IgE, and serum histamine

Hematological tests and clinical chemistry tests were performed on all SD rats and NZW rabbits. Individual results are presented in the S3 and S4 Tables.

In SD rats, there were no polysorbate 20 (0.02, 0.1, and 0.4 mg/kg) or HSA (0.06 and 0.12 mg/kg) related changes in the hematology parameters. However, in clinical chemistry, the levels of albumin/globulin ratio, total bilirubin, glucose, sodium, and chloride were significantly different in the polysorbate 20 or HSA administered groups compared to the vehicle control group (Tables 2 and 3). Furthermore, in the allergic reaction test (ELISA analysis of serum IgE and histamine), significantly higher serum histamine levels were found in the female group administered HSA at a dose of 0.06 and 0.12 mg/kg compared to the vehicle control group (Table 3, Kruskal-Wallis test, $p < 0.05$).

In NZW rabbits, there were no changes in the hematology and clinical chemistry parameters

in the polysorbate 20 (0.02, 0.1, and 0.4 mg/kg/site) or HSA (0.06 and 0.12 mg/kg/site) administered animals. There was also no significant increase or decrease in the level of serum IgE and histamine in NZW rabbits administered polysorbate 20 and HSA compared to the vehicle control group.

Table 2. The level of hematology, clinical chemistry, serum IgE, and histamine in male Sprague-Dawley (SD) rats in the current study.

Category		Median [range]					
		G1	G2	G3	G4	G5	G6
Hematological findings	RBC (10^6cells/μL)	7.3 [7.0-8.0]	7.4 [7.0-7.8]	7.4 [6.9-7.7]	7.5 [7.0-7.9]	7.5 [7.2-7.9]	7.4 [6.9-7.8]
	HGB (g/dL)	14.5 [14.0-15.1]	14.5 [13.9-15.4]	14.7 [13.6-15.7]	14.4 [14.2-15.7]	14.8 [14.3-15.0]	14.5 [13.4-15.2]
	HCT (%)	46.2 [45.2-47.9]	46 [44.3-48.9]	46.4 [42.8-47.9]	45.9 [44.8-48.5]	46.7 [45.2-47.5]	46.1 [42.4-48.5]
	MCV (fL)	63.2 [60.0-65.8]	62.7 [60.7-64.5]	62.4 [61.6-65.4]	62.1 [60.1-63.7]	61.6 [59.4-64.9]	61.9 [61.8-62.4]
	MCH (pg)	19.8 [18.9-20.4]	19.7 [19.3-20.4]	19.8 [19.4-20.5]	19.6 [18.6-20.5]	19.6 [18.7-20.4]	19.5 [19.0-20.0]
	MCHC (g/dL)	31.3 [30.9-32.1]	31.6 [31.4-31.9]	31.6 [31.1-32.8]	31.4 [31-32.3]	31.6 [31.2-32.1]	31.6 [30.7-32.1]
	RETIC (10^9cells/μL)	222 [186.6-246.4]	210 [188-252]	226 [185-257]	243 [225-248]	211.2 [161-221]	222 [183-257]
	PLT (10^3 cells/μL)	1149 [993-1429]	1132 [1050-1428]	1193 [885-1382]	1185 [1087-1352]	1217 [1155-1306]	1161 [1047-1326]
	WBC (10^3 cells/μL)	9.5 [8.6-14.5]	11.2 [8.4-13.7]	9.9 [6.5-13.1]	8.4 [7.1-9.9]	7.4 [4.1-9.3]	8.1 [5.4-11.4]
Biochemical findings	TP (g/dL)	5.9 [5.7-6.1]	6.1 [5.8-6.3]	6.0 [5.6-6.3]	5.9 [5.8-6.4]	6.2 [5.7-6.3]	6.1 [5.8-6.4]
	ALB (g/dL)	3.9 [3.7-4.0]	3.9 [3.8-4.0]	3.9 [3.6-4.0]	3.9 [3.7-3.9]	4.0 [3.8-4.1]	3.9 [3.7-4.1]
	A/G ratio	1.9 [1.7-2.0]	1.8 [1.7-1.9]	1.8 [1.7-1.9]	1.8 [1.6-1.9]	1.9 [1.7-2.0]	1.7 [1.7-1.8]*
	T-BIL (mg/dL)	0.04 [0.03-0.06]	0.05 [0.04-0.06]	0.04 [0.02-0.06]	0.03 [0.02-0.04]	0.03 [0.01-0.04]	0.04 [0.02-0.07]
	ALP (U/L)	572 [507-696]	711 [507-903]	531 [425-684]	564 [497-671]	567 [444-658]	644 [510-839]
	AST (U/L)	91 [71-111]	92.5 [78-269]	92 [80-107]	79.5 [70-101]	109 [91-120]	101 [67-134]
	ALT (U/L)	26 [21-44]	30.5 [25-127]	28.5 [19-38]	23 [18-29]	27 [24-42]	26 [18-35]
	CREA (mg/dL)	0.44 [0.4-0.47]	0.44 [0.39-0.53]	0.47 [0.38-0.49]	0.43 [0.38-0.52]	0.41 [0.37-0.44]	0.43 [0.39-0.44]
	BUN (mg/dL)	12.2 [8.9-13.0]	12.2 [9.9-16.2]	12.3 [9.5-13.4]	9.8 [8.8-12.9]	11.0 [9.9-13.3]	11.1 [9.2-12.2]
	T-CHO (mg/dL)	70 [63-86]	77.5 [64-93]	77 [60-89]	70 [58-92]	64.5 [52-83]	85 [70-89]
	TG (mg/dL)	81.5 [61-104]	98.5 [53-141]	106 [70-181]	82.5 [70-148]	82.5 [42-104]	86.5 [45-173]
	GLU (mg/dL)	125.5 [119-140]	109 [101-127]	101 [93-117]*	121 [88-142]	102 [89-109]*	92.5 [84-123]*
	CA (mg/dL)	10.1 [9.9-10.5]	10.3 [10.1-10.4]	10.2 [9.8-10.4]	10.4 [10.1-10.5]	9.9 [9.8-10.4]	10.2 [9.8-10.8]
	IP (mg/dL)	9.0 [8.1-9.4]	8.8 [8.2-9.2]	8.7 [8.2-9.1]	9.0 [8.2-9.0]	9.0 [8.8-9.3]	8.9 [8.6-9.3]
	GGT (IU/L)	0.36 [0-0.87]	0.12 [0-1.0]	0.67 [0-1.74]	0.24 [0-1]	0.12[0-0.53]	0.32 [0-1.71]
	CK (U/L)	493 [430-724]	526 [454-630]	574 [389-812]	458 [296-577]	735 [584-913]	696 [334-914]
	Na (mmol/L)	143 [142-144]	144 [144-147]	144 [142-145]	145 [143-146]**	144 [143-145]	145 [144-147]*
	K (mmol/L)	4.7 [4.3-4.9]	4.7 [4.5-4.8]	4.8 [4.4-4.9]	4.9 [4.4-5.2]	4.9 [4.8-5.2]	5.0 [4.5-5.1]
	Cl (mmol/L)	101 [100-102]	103 [102-105]	102 [98.3-104]	103 [102-105]	102 [99-103]	102 [101-105]
ELISA analysis	IgE (ng/mL)	0.23 [0.187-0.284]	0.263 [0.24-0.302]	0.246 [0.206-0.309]	0.183 [0.156-0.203]	0.190 [0.157-0.266]	0.189 [0.174-0.201]
	Histamine (ng/mL)	0.48 [0.35-0.591]	0.3625 [0.305-0.555]	0.38 [0.281-0.519]	0.414 [0.335-0.532]	0.531[0.352-0.651]	0.416 [0.22-0.487]

*vs. Vehicle Control group: $p < 0.05$, ** vs. Vehicle Control group: $p < 0.01$

Statistically significant items (Kruskal-Wallis test) compared to the vehicle control are shaded gray.

Table 3. The level of hematology, clinical chemistry, serum IgE, and histamine in female Sprague-Dawley (SD) rats in the current study.

Category		Median [range]					
		G1	G2	G3	G4	G5	G6
Hematological findings	RBC (10^6cells/µL)	7.2 [6.5-7.7]	6.7 [6.2-7.1]	7.1 [6.8-7.3]	7.4 [7.0-7.5]	7.3 [6.9-8.2]	7.3 [7.0-7.8]
	HGB (g/dL)	14.2 [13.4-14.9]	13.9 [12.8-14.6]	13.9 [13.4-14.4]	14.5 [14.3-14.9]	14.5 [14.2-15.8]	14.3 [13.7-15.3]
	HCT (%)	42.7 [40.5-44.9]	42.9 [38.9-44.7]	42 [41.0-43.1]	44.6 [44.0-44.8]	44.4 [41.7-47.1]	44.3 [42.1-46.5]
	MCV (fL)	59.0 [57.6-62]	62.7 [59.4-65.9]	59.1 [57.8-63.0]	61.0 [59.4-63.1]	60.4 [57.3-62.0]	60.0 [59.8-62.5]
	MCH (pg)	19.5 [19.4-20.6]	20.4 [20.3-20.6]	19.5 [19.1-21.1]	20.1 [19.3-20.6]	19.9 [19.0-20.5]	19.7 [19.5-20.5]
	MCHC (g/dL)	33.2 [32.2-33.9]	32.6 [31.3-34.3]	33.4 [32.7-33.5]	32.8 [32.4-33.4]	32.6 [32.5-33.9]	32.9 [32.2-33.0]
	RETIC (10^9cells/µL)	163 [118-176]	174 [120-222]	157 [119-198]	172 [152-181]	148 [101-182]	156 [145-188]
	PLT (10^3 cells/µL)	1153 [519-1382]	1021 [104-1210]	1278 [817-1476]	1150 [969-1436]	1164 [948-1211]	1191 [1022-1370]
	WBC (10^3 cells/µL)	3.4 [2.4-4.8]	4.6 [1.0-7.0]	4.4 [3.3-8.6]	5.5 [3.0-9. 8]	4.5 [3.6-5.5]	4.4 [3.4-5.6]
Biochemical findings	TP (g/dL)	6.1 [5.9-6.2]	6.1 [5.7-6.4]	5.8 [5.7-6.9]	6.1 [5.9-6.1]	6.1 [5.7-6.2]	6.1 [5.8-6.5]
	ALB (g/dL)	4.2 [3.9-4.2]	4.1 [3.7-4.3]	3.9 [3.5-4.6]	4.0 [3.7-4.0]	3.9 [3.8-4.0]	4.0 [3.8-4.3]
	A/G ratio	2.1 [1.9-2.2]	2.0 [1.8-2.2]	1.9 [1.5-2.1]	1.9 [1.7-2.0]	1.8 [1.8-2.0]	1.9 [1.8-2.0]
	T-BIL (mg/dL)	0.07 [0.06-0.07]	0.03 [0.02-0.04]*	0.06 [0.01-0.07]	0.03 [0-0.06]*	0.04 [0.02-0.06]	0.04 [0.01-0.07]
	ALP (U/L)	355 [324-413]	449 [367-612]	419 [369-538]	389 [321-590]	436 [325-600]	517 [334-573]
	AST (U/L)	80 [77-89]	101 [80-109]	79 [62-127]	91 [87-98]	81 [73-98]	84 [69-105]
	ALT (U/L)	27 [23-27]	28 [21-35]	26 [21-49]	26 [19-31]	32 [29-39]	28 [26-37]
	CREA (mg/dL)	0.41 [0.39-0.47]	0.42 [0.41-0.52]	0.43 [0.39-0.48]	0.42 [0.36-0.47]	0.39 [0.37-0.42]	0.42 [0.38-0.48]
	BUN (mg/dL)	17.7 [14.7-26.1]	15.8 [12.3-17.8]	14.8 [14.3-18.8]	13.3 [10.4-16.9]	14.5 [12.2-17.2]	17.9 [13.3-18.9]
	T-CHO (mg/dL)	87 [66-108]	89 [73-95]	84 [77-104]	90 [63-100]	83 [79-101]	102 [74-127]
	TG (mg/dL)	64 [37-122]	42 [36-77]	52 [40-58]	32 [25-113]	53 [33-74]	64 [41-89]
	GLU (mg/dL)	116 [97-121]	118 [111-148]	102 [98-113]	98 [93-118]	108 [106-130]	125 [105-137]
	CA (mg/dL)	10.1 [9.8-10.3]	9.9 [9.6-10.3]	10.1 [9.7-10.8]	9.9 [9.8-10.5]	10.3 [10.2-10.4]	10.5 [10.1-10.7]
	IP (mg/dL)	7.7 [6.7-7.8]	7.5 [6.4-7.7]	7.7 [6.8-8.0]	7.5 [6.8-8.0]	7.6 [7.3-8.3]	8.1 [7.6-8.3]
	GGT (IU/L)	0.38 [0-0.55]	0.12 [0-0.37]	0.0 [0-0.57]	0.29 [0-0.65]	0.11 [0-0.71]	0.46 [0-0.74]
	CK (U/L)	606 [552-637]	745 [490-840]	423 [292-936]	484 [405-554]	404 [331-565]	480 [203-888]
	Na (mmol/L)	141 [140-142]	142 [140-143]	142 [139-143]	142 [141-143]	141 [140-142]	141 [140-142]
	K (mmol/L)	4.4 [4.3-4.6]	4.2 [4.1-4.8]	4.5 [3.9-4.9]	4.4 [4.2-4.7]	4.6 [4.2-4.8]	4.5 [3.6-5.0]
	Cl (mmol/L)	100 [99.5-101]	103 [101-104]*	104 [101-104]**	102 [98.8-105]	102 [101-103]	102 [101-102]
ELISA analysis	IgE (ng/mL)	0.245 [0.132-0.263]	0.214 [0.183-0.229]	0.235 [0.173-0.376]	0.204 [0.142-0.299]	0.182 [0.139-0.265]	0.175 [0.158-0.245]
	Histamine (ng/mL)	0.278 [0.172-0.336]	0.291 [0.232-0.476]	0.363 [0.317-0.46]	0.224 [0.194-0.361]	0.43 [0.327-0.976]*	0.536 [0.353-0.648]*

*vs. Vehicle Control group: $p < 0.05$, ** vs. Vehicle Control group: $p < 0.01$

Statistically significant items (Kruskal-Wallis test) compared to the vehicle control are shaded gray.

Findings in necropsy and histopathology

During the necropsy on all SD rats, polysorbate 20 or HSA related macroscopic findings were not observed, including in external appearance, internal organs, and administration sites. In histopathological examination of the administration site, mild macrophage infiltration (vehicle control group: 6 male and 5 female rats; polysorbate 20-0.02 mg/kg: 6 male and 5 female rats; polysorbate 20-0.1 mg/kg: 4 male and 4 female rats; polysorbate 20-0.4 mg/kg: 6 male and 5 female rats; HSA-0.06 mg/kg: 6 male and 5 female rats; HSA-0.12 mg/kg: 6 male and 5 female rats) and neovascularization (vehicle control group: 6 male and 5 female rats; polysorbate 20-0.02 mg/kg: 4 male and 5 female rats; polysorbate 20-0.1 mg/kg: 3 male and 3 female rats; polysorbate 20-0.4 mg/kg: 4 male and 2 female rats; HSA-0.06 mg/kg: 3 male and 4 female rats; HSA-0.12 mg/kg: 5 male and 4 female rats) were observed with a similar degree and frequency in all administration groups, including the vehicle control group. In addition, inflammatory cells infiltration (1 male rat) and fat infiltration (2 female rats) were observed in the vehicle control group. Among the polysorbate 20 administration group, fat infiltration (3 female rats) was observed in the 0.02 mg/kg group; fat infiltration, giant cells, and necrosis (1 male rat) were observed in the 0.1 mg/kg group; inflammatory cells infiltration (1 male rat) and fat infiltration (1 female rat) were observed in the 0.4 mg/kg group. Among the HSA administration group, inflammatory cells infiltration, fibrosis, fat infiltration, and necrosis for 1 male rat, fibrosis for 2 female rats, and inflammatory cells infiltration, fat infiltration, and necrosis for 1 female were observed in the 0.06 mg/kg group; fibrosis (1 male rat) and fat infiltration (1 female rats) were observed in the 0.12 mg/kg group (Fig 2).

In the case of the NZW rabbits, macroscopic findings at necropsy and histopathological findings at the sites of administration were not observed regardless of the administered substance (Fig 3).

Fig 2. Histopathological section of the administration site in the four-week repeated intramuscular dose toxicity study in Sprague-Dawley (SD) rats. Hematoxylin & eosin stained histopathological section (original magnification 100x) of the gastrocnemius muscle of the SD rats: a) Vehicle control group, male; b) Vehicle control group, female; c) Polysorbate 20-0.02 mg/kg, male; d) Polysorbate 20-0.02 mg/kg, female; e) Polysorbate 20-0.1 mg/kg, male; f) Polysorbate 20-0.1 mg/kg, female; g) Polysorbate 20-0.4 mg/kg, male; h) Polysorbate 20-0.4 mg/kg, female; i) Human serum albumin-0.06 mg/kg, male; j) Human serum albumin-0.06 mg/kg, female; k) Human serum albumin-0.12 mg/kg, male; l) Human serum albumin-0.12 mg/kg, female. Mild macrophage infiltration, neovascularization, inflammatory cells infiltration, and fat infiltration were observed with a similar degree and frequency in all administration groups.

Fig 3. Histopathological section of the administration site in the intradermal irritation study in New Zealand White (NZW) rabbits. Hematoxylin & eosin stained histopathological section (original magnification 50x) of the dorsal back skin of the NZW rabbits: a) Vehicle control group; b) Polysorbate 20-0.02 mg/kg; c) Polysorbate 20-0.1 mg/kg; d) Polysorbate 20-0.4 mg/kg; e) Human serum albumin (HSA)-0.06 mg/kg; f) HSA-0.12 mg/kg, male. No histopathological findings were found at the site of administration in all administration groups.

Discussion

Polysorbate 20 was approved by the regulatory authorities as an additive in foods, cosmetics, eye drops, and tablets in various countries [20,21]. Therefore, most clinical and nonclinical safety studies of polysorbate 20 have been conducted using non-invasive routes of administration (e.g., oral, skin, and ophthalmic administration) [27,28]. In particular, as a food additive, polysorbate 20 has various *in vivo* safety data, including *in vitro* genotoxicity and DNA reactivity based on structural

alerts [29]. Recently, polysorbate 20, which was one of the excipients of CORETOX® approved by the Ministry of Food and Drug Safety (MFDS, Republic of Korea), has been used in humans via an intramuscular route of administration [18]. However, there are few studies on the safety of polysorbate 20 when administered intramuscularly. In a study conducted in four monkeys, it was reported that intramuscular injection of 275 mg/kg of polysorbate20 for 20 days did not cause toxicity in the liver and kidney [30]. This study did not evaluate clinical symptoms, body weight, or hematology and clinical chemistry, which are essential criteria for evaluating toxicity [31]. Therefore, the current study was conducted to verify the safety of intramuscular administration of polysorbate 20 through four-week repeated intramuscular dose toxicity in SD rats and an intradermal irritation study in NZW rabbits. This study is the first to fully evaluate the *in vivo* safety of polysorbate 20 when administered intramuscularly based on current guidelines of non-clinical safety research.

To evaluate the systemic toxicity of intramuscular injection of polysorbate 20, a four-week repeated dose toxicity study in SD rats was conducted. According to the MFDS, the clinical dosage of CORETOX®, which contains 1 mg of polysorbate 20 in one vial, ranged from 0.2 vial (treatment of forehead wrinkles) to 3.6 vials (stiff muscles in the upper limbs after a stroke) [18]. Thus, the dose (0.02, 0.1, and 0.4 mg/kg) of polysorbate 20 in the current study was established based on the therapeutic indication of CORETOX® in humans and the animal equivalent dose calculation factor [23]. In addition, 0.9% saline was set as the vehicle control substance, while 0.06 and 0.12 mg/kg doses of HSA, which has an *in vivo* safety profile accumulated over the years for intramuscular administration, was set as a comparative substance [10,12].

Generally, mortality, body weight, and clinical symptoms have the most significance in repeated dose toxicity studies [32,33]. Furthermore, changes in the levels of hematology and clinical chemistry in laboratory animals could enable more accurate predictions of human toxicity [34,35]. In the current study, adverse effects, including mortality, clinical symptoms, and macroscopic findings on necropsy, were not found at any dose level of HSA and polysorbate 20 administered during the

study period in all SD rats. In addition, significant changes in the body weight and the level of hematology were not observed in the polysorbate 20 and HSA administered groups compared to the vehicle control group. In the clinical chemistry test and allergy reaction test, significant differences in albumin/globulin ratio, total bilirubin, glucose, sodium, and chloride were observed in a polysorbate 20 or HSA administration group compared to the vehicle control group. However, these changes had no toxicological significance as they were not dose-responsive and the levels were within the normal range for the same age of SD rats [36,37]. This indicated that intramuscular administration of 0.02, 0.1, and 0.4 mg/kg dose of polysorbate 20 did not cause systemic toxicity in SD rats.

Histopathological examination of the administration site was conducted to observe the local toxicity of the polysorbate 20 in all SD rats. At the administration site, macrophage infiltration, neovascularization, giant cell, fibrosis, and necrosis were observed to a similar degree and frequency in all administration groups regardless of the administered substance. However, these findings were observed in most of the administered groups without a dose response, including the vehicle control group. Furthermore, inflammatory lesions could occur in the body of SD rats as a defense mechanism against intramuscular injection of any foreign substances [38]. Therefore, those findings were presumed to be due to the intramuscular injection rather than any effect of polysorbate 20. In other words, there were no toxicological changes at the site of administration after intramuscular administration of polysorbate 20 based on histopathology. Furthermore, serum IgE and histamine levels were measured to determine whether allergic reactions occurred after the administration of polysorbate 20 in all SD rats. In addition to clinical symptoms, increased levels of serum IgE and histamine are representative indicators of an allergic reaction [39–41]. In the current study, allergic reactions were not observed in response to the administration of polysorbate 20, which indicated that polysorbate 20 is not an allergen in SD rats.

However, despite its very low frequency, side effects in the allergy category have been reported in humans using shampoos (8.4% polysorbate 20, 2 cases among 5.88 million uses), cuticle

softeners (2.0% polysorbate 20, 24 cases among 131 million use), and paste masks (2.0% polysorbate 20, 11 cases among 120.7 million uses) containing polysorbate 20 [20,21]. Therefore, an additional intradermal irritation study was conducted in NZW rabbits to confirm that polysorbate 20 was not an allergen *in vivo*. Traditionally, the intradermal test is the gold standard method for diagnosing allergic reactions [42,43]. If an immune response, such as erythema, crust formation, and edema formation, occurs at the administration site after intradermal administration of a substance, it could be concluded that the substance was an allergen to the administered subjects [25]. In the current study, immune responses of the administration site were not observed at all polysorbate 20 treated NZW rabbits after intradermal administration during the study period, which is the same as the results in the vehicle control group and the HSA administration group. Furthermore, in the polysorbate 20 administration group, toxicological changes based on the histopathological examination of the site of administration and increased levels of serum IgE and histamine were not observed. There were also no toxicologically significant changes, such as changes in the body weight or the hematology and clinical chemistry in all rabbits administered polysorbate 20 and all toxicological values in NZW rabbits were within the normal range for the same age of the NZW rabbits [44,45]. These results of the intradermal irritation study indicated that polysorbate 20 was not an allergen in NZW rabbits, which was consistent with the results of the four-week repeated dose toxicity study in SD rats.

Conclusions

The current study, a four-week repeated intramuscular dose toxicity study in SD rats and an intradermal irritation study in NZW rabbits, provides the basis for the *in vivo* safety profile of intramuscular administration of polysorbate 20, an excipient of newly developed BoNT/A products (CORETOX®). Polysorbate 20, which did not induce systemic and local toxicities in this study, was considered to be a safe substance for intramuscular administration, just like HSA for which a safety

profile has been obtained over the years. In addition, since polysorbate 20 is a synthetic product rather than an animal-derived product, it has the advantages of a consistent quality of the product, ease of manufacture and supply, and a low risk of pathogen transmission compared to HSA. Therefore, the findings of this study suggest the scientific reliability of the use of polysorbate 20 as an alternative to HSA, which is used as an excipient for most BoNT/A products and various pharmaceuticals, particularly in terms of its safety.

Acknowledgments

Not applicable

Author contributions

Conceptualization: JK, SK, WCS, WhK

Data curation: JK, SK, MSP, CHR, GHY

Formal analysis: JK, SK

Funding Acquisition: WCS

Investigation and Methodology: JK, SK, MSP, CHR, GHY

Project administration: MSP, WCS, WhK

Resources: CHR, GHY

Supervision: WCS, WhK

Validation: MSP, CHR, GHY

Visualization: JK

Writing – Original Draft Preparation: JK, SK

Writing – Review & Editing: JK, SK, MSP, CHR, GHY, WCS, WhK

References

1. Schantz EJ, Johnson EA. Properties and use of botulinum toxin and other microbial neurotoxins in medicine. Microbiol Mol Biol Rev. 1992;56: 80–99.
2. Brin MF. Development of future indications for BOTOX®. Toxicon. 2009;54: 668–674.
3. Naumann M, Carruthers A, Carruthers J, Aurora SK, Zafonte R, Abu- Shakra S, et al. Meta- analysis of neutralizing antibody conversion with onabotulinumtoxinA (BOTOX®) across multiple indications. Mov Disord. 2010;25: 2211–2218.
4. Clark RP, Berris CE. Botulinum toxin: a treatment for facial asymmetry caused by facial nerve paralysis. Plast Reconstr Surg. 1989;84: 353–355.
5. Carruthers JDA, Carruthers JA. Treatment of glabellar frown lines with C. botulinum- A exotoxin. J Dermatol Surg Oncol. 1992;18: 17–21.
6. Ahn CS, Davis SA, Dabade TS, Williford PM, Feldman SR. Noncosmetic Skin- Related Procedures Performed in the United States: An Analysis of National Ambulatory Medical Care Survey Data from 1995 to 2010. Dermatologic Surg. 2013;39: 1912–1921.
7. Benedetto A V. What's new in cosmetic dermatology. Dermatol Clin. 2019;37: 117–128.
8. Park J-Y, Sunga O, Wanitphakdeedecha R, Frevert J. Neurotoxin Impurities: A Review of Threats to Efficacy. Plast Reconstr Surg Glob Open. 2020;8.
9. Fanali G, Di Masi A, Trezza V, Marino M, Fasano M, Ascenzi P. Human serum albumin: from bench to bedside. Mol Aspects Med. 2012;33: 209–290.
10. Zhou T, Lu S, Liu X, Zhang Y, Xu F. Review of the rational use and adverse reactions to human serum albumin in the People's Republic of China. Patient Prefer Adherence. 2013;7: 1207.

11. Bosse D, Praus M, Kiessling P, Nyman L, Andresen C, Waters J, et al. Phase I comparability of recombinant human albumin and human serum albumin. J Clin Pharmacol. 2005;45: 57–67.

12. Malhotra R, Huilgol SC, Selva D. Botulinum toxin and human serum albumin. Arch Ophthalmol. 2003;121: 1661.

13. Chen Z, He Y, Shi B, Yang D. Human serum albumin from recombinant DNA technology: challenges and strategies. Biochim Biophys Acta (BBA)-General Subj. 2013;1830: 5515–5525.

14. Matejtschuk P, Dash CH, Gascoigne EW. Production of human albumin solution: a continually developing colloid. Br J Anaesth. 2000;85: 887–895.

15. Chamberland ME, Alter HJ, Busch MP, Nemo G, Ricketts M. Emerging infectious disease issues in blood safety. Emerg Infect Dis. 2001;7: 552.

16. Tumber PS, Louis I. Botulinum toxin type a therapy and human serum albumin. Anesthesiol J Am Soc Anesthesiol. 2006;104: 1108.

17. Norsworthy PJ, Thompson AGB, Mok TH, Guntoro F, Dabin LC, Nihat A, et al. A blood miRNA signature associates with sporadic Creutzfeldt-Jakob disease diagnosis. Nat Commun. 2020;11. doi:10.1038/s41467-020-17655-x

18. Lee J, Chun MH, Ko YJ, Lee S-U, Kim DY, Paik N-J, et al. Efficacy and safety of MT10107 (Coretox®) in post-stroke upper limb spasticity treatment: A randomized, double-blind, active drug-controlled, multi-center, phase III clinical trial. Arch Phys Med Rehabil. 2020.

19. Kwak S, Kang W, Rhee C-H, Yang G-H, Cruz DJM. Comparative Pharmacodynamics Study of 3 Different Botulinum Toxin Type A Preparations in Mice. Dermatologic Surg. 2020;46: e132–e138.

20. Moore J. Final report on the safety assessment of polysorbates 20, 21, 40, 60, 61, 65, 80, 81, and 85. J Am Coll Toxicol. 1984;3: 1–82.

21. Lanigan RS. Final report on the safety assessment of PEG-20 sorbitan cocoate; PEG-40 sorbitan diisostearate; PEG-2,-5, and-20 sorbitan isostearate; PEG-40 and-75 sorbitan lanolate; PEG-10,-40,-44,-75, and-80 sorbitan laurate; PEG-3, and-6 sorbitan oleate; PEG-80 sorb. Int J Toxicol. 2000;19: 43–89.

22. Percie du Sert N, Ahluwalia A, Alam S, Avey MT, Baker M, Browne WJ, et al. Reporting animal research: Explanation and elaboration for the ARRIVE guidelines 2.0. PLoS Biol. 2020;18: e3000411.

23. Nair AB, Jacob S. A simple practice guide for dose conversion between animals and human. J basic Clin Pharm. 2016;7: 27.

24. Diehl K, Hull R, Morton D, Pfister R, Rabemampianina Y, Smith D, et al. A good practice guide to the administration of substances and removal of blood, including routes and volumes. J Appl Toxicol An Int J. 2001;21: 15–23.

25. Iso B, STANDARD B. Biological evaluation of medical devices. Part. 2009;1: 10993.

26. Leary SL, Underwood W, Anthony R, Cartner S, Corey D, Grandin T, et al. AVMA guidelines for the euthanasia of animals: 2013 edition. American Veterinary Medical Association Schaumburg, IL; 2013.

27. Rowe RC, Sheskey P, Quinn M. Handbook of pharmaceutical excipients. Libros Digitales-Pharmaceutical Press; 2009.

28. Lewis RJ, Sax N. Sax's dangerous properties of industrial materials. New York; 1996.

29. (ANS) EP on FA and NS added to F. Scientific Opinion on the re- evaluation of polyoxyethylene sorbitan monolaurate (E 432), polyoxyethylene sorbitan monooleate (E 433), polyoxyethylene sorbitan monopalmitate (E 434), polyoxyethylene sorbitan monostearate (E 435) and polyoxyethylene sorbitan tristearate (E 436) as food additives. EFSA J. 2015;13:

4152.

30. Krantz JC, Carr CJ, Bird JG, Cook S. SUGAR ALCOHOLS: XXVI. Pharmacodynamic studies of polyoxyalkylene derivatives of hexitol anhydride partial fatty acid esters. J Pharmacol Exp Ther. 1948;93: 188–195.

31. Guideline ICHHT. Guidance on nonclinical safety studies for the conduct of human clinical trials and marketing authorization for pharmaceuticals M3 (R2). International conference on harmonisation of technical requirements for registration of pharmaceuticals for human use. 2009.

32. Liu T, Li L, Teng X, Huang X, Liu H, Chen D, et al. Single and repeated dose toxicity of mesoporous hollow silica nanoparticles in intravenously exposed mice. Biomaterials. 2011;32: 1657–1668.

33. Shende P, Kulkarni YA, Gaud RS, Deshmukh K, Cavalli R, Trotta F, et al. Acute and repeated dose toxicity studies of different β- cyclodextrin- based nanosponge formulations. J Pharm Sci. 2015;104: 1856–1863.

34. El Hilaly J, Israili ZH, Lyoussi B. Acute and chronic toxicological studies of Ajuga iva in experimental animals. J Ethnopharmacol. 2004;91: 43–50.

35. Rhiouani H, El-Hilaly J, Israili ZH, Lyoussi B. Acute and sub-chronic toxicity of an aqueous extract of the leaves of Herniaria glabra in rodents. J Ethnopharmacol. 2008;118: 378–386.

36. Lillie LE, Temple NJ, Florence LZ. Reference values for young normal Sprague-Dawley rats: weight gain, hematology and clinical chemistry. Hum Exp Toxicol. 1996;15: 612–616.

37. Petterino C, Argentino-Storino A. Clinical chemistry and haematology historical data in control Sprague-Dawley rats from pre-clinical toxicity studies. Exp Toxicol Pathol. 2006;57: 213–219.

38. Suttie AW. Boorman's Pathology of the Rat. Elsevier; 2015.

39. Wachholz PA, Dearman RJ, Kimber I. Detection of allergen-specific IgE antibody responses. J Immunotoxicol. 2005;1: 189–199.

40. Barbee RA, Brown WG, Kaltenborn W, Halonen M. Allergen skin-test reactivity in a community population sample: correlation with age, histamine skin reactions, and total serum immunoglobulin E. J Allergy Clin Immunol. 1981;68: 15–19.

41. Tanizaki Y, Komagoe H, Morinaga H, Kitani H, Goda Y, Kimura I. Allergen-and anti-IgE-induced histamine release from whole blood. Int Arch Allergy Immunol. 1984;73: 141–145.

42. Ruzicka T, Gerstmeier M, Przybilla B, Ring J. Allergy to local anesthetics: comparison of patch test with prick and intradermal test results. J Am Acad Dermatol. 1987;16: 1202–1208.

43. Wood RA, Phipatanakul W, Hamilton RG, Eggleston PA. A comparison of skin prick tests, intradermal skin tests, and RASTs in the diagnosis of cat allergy. J Allergy Clin Immunol. 1999;103: 773–779.

44. Gallego M. Laboratory reference intervals for systolic blood pressure, rectal temperature, haematology, biochemistry and venous blood gas and electrolytes in healthy pet rabbits. Open Vet J. 2017;7: 203–207.

45. Hewitt CD, Innes DJ, Savory J, Wills MR. Normal biochemical and hematological values in New Zealand white rabbits. Clin Chem. 1989;35: 1777–1779.

Supporting information

S1 Table. Individual body weights of male (n: 36) and female (n: 30) Sprague-Dawley (SD) rats in the current study.

S2 Table. Individual body weights of male New Zealand White (NZW) rabbits (n: 18) in the current study.

S3 Table. Individual levels of hematology, clinical chemistry, serum IgE, and histamine in Sprague-Dawley (SD) rats in the current study.

S4 Table. Individual levels of hematology, clinical chemistry, serum IgE, and histamine in New Zealand White (NZW) rabbits in the current study.

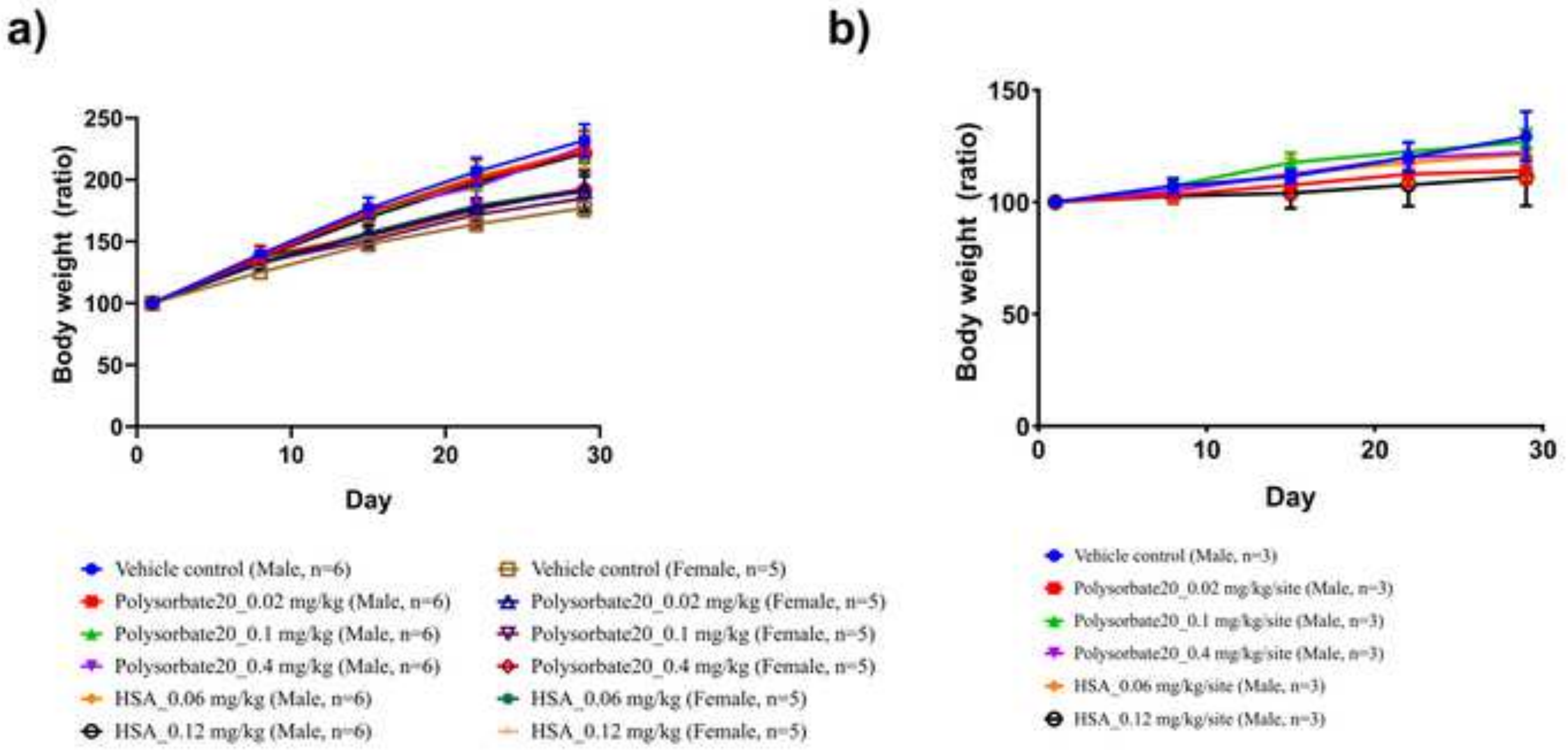
a)
Body weight (ratio)
250
200
150
100
50
0
0
10
20
30
Day
Vehicle control (Male, n=6)
Polysorbate20_0.02 mg/kg (Male, n=6)
Polysorbate20_0.1 mg/kg (Male, n=6)
Polysorbate20_0.4 mg/kg (Male, n=6)
HSA_0.06 mg/kg (Male, n=6)
HSA_0.12 mg/kg (Male, n=6)
Vehicle control (Female, n=5)
Polysorbate20_0.02 mg/kg (Female, n=5)
Polysorbate20_0.1 mg/kg (Female, n=5)
Polysorbate20_0.4 mg/kg (Female, n=5)
HSA_0.06 mg/kg (Female, n=5)
HSA_0.12 mg/kg (Female, n=5)
b)
Body weight (ratio)
150
100
50
0
0
10
20
30
Day
Vehicle control (Male, n=3)
Polysorbate20_0.02 mg/kg/site (Male, n=3)
Polysorbate20_0.1 mg/kg/site (Male, n=3)
Polysorbate20_0.4 mg/kg/site (Male, n=3)
HSA_0.06 mg/kg/site (Male, n=3)
HSA_0.12 mg/kg/site (Male, n=3)

Click here to access/download;Figure;Fig 2.tif

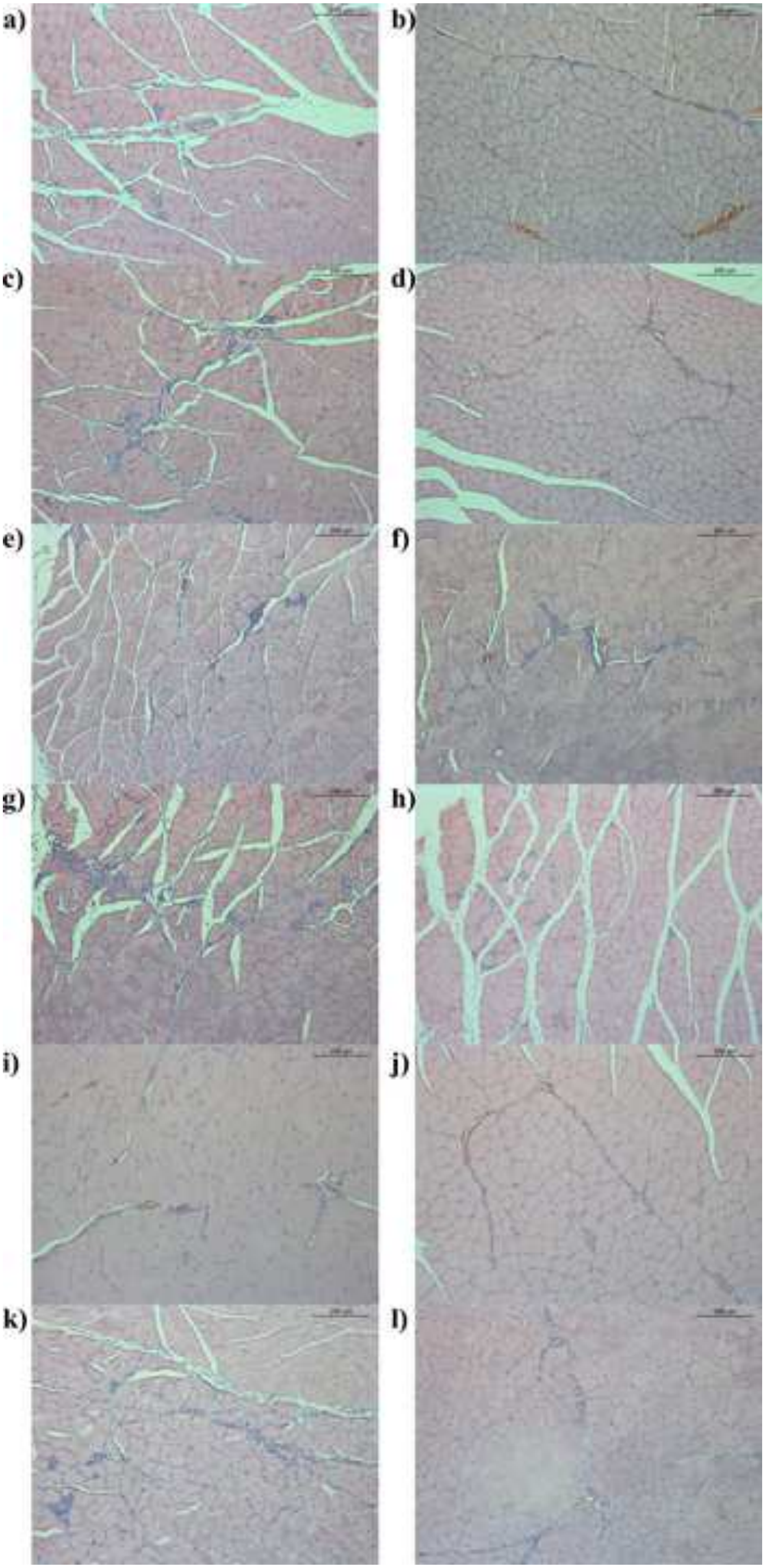

Click here to access/download;Figure;Fig 3.tif

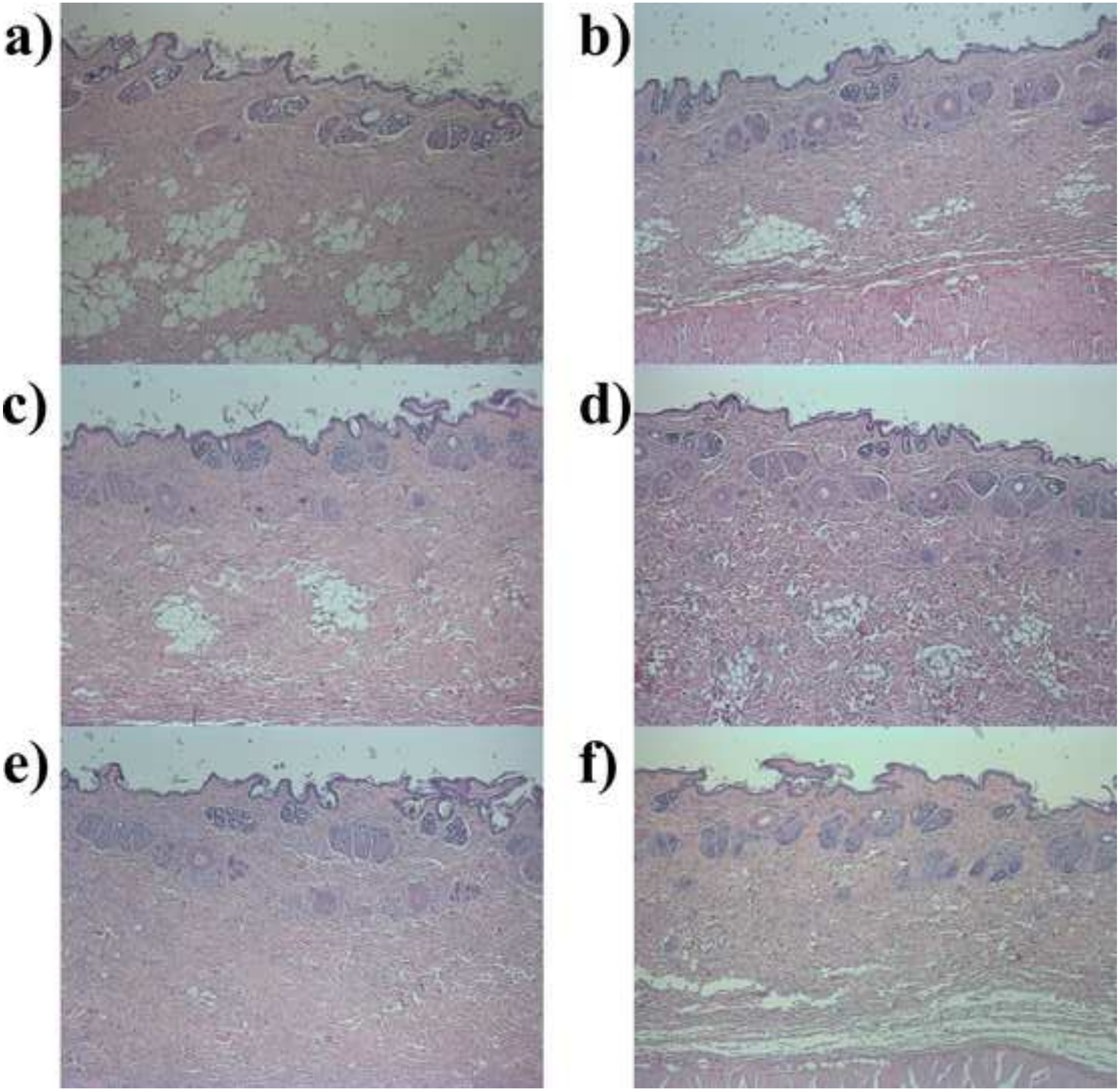

Click here to access/download
Supporting Information
S1 Table.xlsx

Click here to access/download
Supporting Information
S2 Table.xlsx

Click here to access/download
Supporting Information
S3 Table.xlsx

Supporting Information 4

Click here to access/download
Supporting Information
S4 Table.xlsx
