# Supplementary material for: Safety verification for polysorbate 20, pharmaceutical excipient for intramuscular administration, in Sprague-Dawley rats and New Zealand White rabbits
Source: PLoS One. 2021 Aug 27;16(8):e0256869. doi: 10.1371/journal.pone.0256869 (PMC8396741; doi:10.1371/journal.pone.0256869)
Supplement: Attachment — Submitted filename: PONE-D-21-03019_R1_reviewer.pdf [file pone.0256869.s007.pdf]

PLOS ONE

Safety verification for polysorbate 20, pharmaceutical excipient for intramuscular administration, in Sprague-Dawley rats and New Zealand White rabbits

--Manuscript Draft--

**Manuscript Number:**	PONE-D-21-03019R1
**Article Type:**	Research Article
**Full Title:**	Safety verification for polysorbate 20, pharmaceutical excipient for intramuscular administration, in Sprague-Dawley rats and New Zealand White rabbits
**Short Title:**	In vivo safety evaluation of polysorbate 20
**Corresponding Author:**	Won-ho Kang<br>MedyTox Inc.<br>Suwon-si, KOREA, REPUBLIC OF
**Keywords:**	Botulinum toxin type A products; Pharmaceutical excipient; Polysorbate 20; Human serum albumin
**Abstract:**	Human serum albumin (HSA) has been widely used as a pharmaceutical excipient in Botulinum toxin serotype A (BoNT/A) products that are indicated for use in therapeutics and cosmetics. However, HSA as a human-derived material has some concerns, such as the potential risk of transmission of infectious agents, an insufficient supply, and difficulty in maintaining a certain quality. For those reasons, newly developed BoNT/A products (CORETOX ® , Medytox, Inc., Republic of Korea) contained polysorbate 20, a non-human-derived excipient, to replace the HSA. However, most safety studies of polysorbate 20 have been conducted with non-invasive routes of administration, and thus there are a few studies on the safety of polysorbate 20 when administered intramuscularly. To secure the in vivo safety profile of polysorbate 20, a four-week repeated intramuscular dose toxicity study (0.02, 0.1, and 0.4 mg/kg, one injection every two weeks for a total of three injections) was conducted in 66 Sprague-Dawley (SD) rats. Then, an intradermal irritation study was further conducted with 18 New Zealand White (NZW) rabbits. The toxicological evaluation of HSA (0.06 and 0.12 mg/kg) was also carried out as a comparative substance. Systemic and local toxicities were not observed in any of the SD rats or NZW rabbits based on clinical signs, body weight, hematology, clinical biochemistry, macroscopic findings on necropsy, histopathology of the injection site, and allergic reactions. The current study suggested that intramuscular administration of polysorbate 20 was considered to be safe at a level similar to that of HSA, which has an in vivo safety profile accumulated over the years. This provided the basis for the in vivo safety profile of polysorbate 20 administered intramuscularly and the scientific reliability of the use of polysorbate 20 as an alternative to HSA, which is used as an excipient for various pharmaceuticals in terms of its safety.
**Order of Authors:**	Junhyung Kim
	Seongsung Kwak
	Mi-Sun Park
	Chang-Hoon Rhee
	Gi-Hyeok Yang
	Jangmi Lee
	Woo-Chan Son
	Won-ho Kang
**Response to Reviewers:**	[We have upload a separate file labeled 'Response to Reviewers'.]<br><br>Dear editor and reviewers,<br>We would like to thank the editor and the reviewers for helpful and constructive comments and critiques. We have done our best to revise the manuscript in order to satisfactorily address each of the points raised. We believe that this revised manuscript

Powered by Editorial Manager® and ProduXion Manager® from Aries Systems Corporation

has greatly benefited from these changes and hope that it will be considered satisfactory for publication in PLOS ONE.

Response to Editor's comment
- We are grateful to the editor for the comments and suggestions, which have helped us to improve our manuscript. We have revised the manuscript based on the editor's comments and have provided our point-by-point responses to each comment below.

1. Why did you measure IgE levels and not for example HSA/PS specific IgG?
- Thank you for your comments. Generally, allergic reactions are divided into four types (Type I, II, III, and IV), of which type I and IV are the most common types of drug-related allergy (#1). Therefore, we conducted in vivo test, a standard method, to determine whether polysorbate 20 induces type I or IV allergies. Then, we confirmed that there was no allergic reaction, including abnormalities at the site of administration, due to polysorbate 20 in both studies (four-week repeated intramuscular dose toxicity study in SD rats and intradermal irritation study in NZW rabbits). After in vivo test, since only IgE-mediated anaphylaxis cases (type I allergy) were reported due to polysorbates (#2), we further measured the levels of serum IgE and histamine, which were type I allergy-related factors. The level of IgE and histamine also did not increase after administration of polysorbate 20 in both studies.
-#1: Böhm R, Proksch E, Schwarz T, Cascorbi I. Drug hypersensitivity: diagnosis, genetics, and prevention. Dtsch Arztebl Int. 2018;115: 501.
-#2: Maggio E. Polysorbates, biotherapeutics, and anaphylaxis: a review. Bioprocess Int. 2017.

2. What was the rationale for using the gastrocnemius muscle for injection rather than the thigh muscles?
- We thank you for pointing this out. In the case of BOTOX®, the first FDA-approved Botulinum toxin serotype A (BoNT/A) product, the route of administration was set to gastrocnemius muscle rather than thigh muscle in the toxicity study in rodents (Biologic license application number: 103000). In addition, in the compound muscle action potential (CMAP) and digit abduction scoring (DAS) techniques, which are generally used to evaluate the effectiveness of BoNT/A products, the route of administration was also set to gastrocnemius muscle rather than thigh muscle in the toxicity study in rodents (#1, 2, 3). Therefore, in the current study, the administration route was set to the gastrocnemius muscle for accurate comparison with the previously reported results. We have added the rationale for setting the route of administration to the revised manuscript.
-#1: Kim S-H, Kim S-B, Yang G-H, Rhee C-H. Mouse compound muscle action potential assay: An alternative method to conduct the LD50 botulinum toxin type A potency test. Toxicon. 2012;60: 341–347.
-#2: Aoki KR. A comparison of the safety margins of botulinum neurotoxin serotypes A, B, and F in mice. Toxicon. 2001;39: 1815–1820.
-#3: Oh H-M, Park JH, Song DH, Chung ME. Efficacy and safety of a new botulinum toxin type A free of complexing proteins. Toxins (Basel). 2016;8: 4.

3. We recommend rephrasing Lines 202-203 to removed the toxicologists' bias of looking for death in a study ("all animals survived..") to "..all animals remained well throughout the study and showed no clinical signs of disease, pain or distress.". Please also replace uses of the word 'symptoms' (what a patient reports) instead of 'signs' (what is observable).
- Thank you for your recommendation. As you suggest, we have revised the phrase "all SD rats survived throughout the study period" in the revised manuscript. In addition, we have modified the word 'symptoms' to 'signs' throughout the revised manuscript.

4. We note that the manuscript focuses mainly on the implications of this study for the development of cosmetics, and therefore request that you explicitly assert and emphasize the medical implications of this study in order to show the full range of applications of your findings.
- Thank you for your recommendation. As you suggest, we have modified the "Introduction" and "Conclusion" part accordingly in the revised manuscript. We have emphasized the significance of this study and mentioned the scope of application of our findings in the future.

Powered by Editorial Manager® and ProduXion Manager® from Aries Systems Corporation

Response to Reviewers' comments (Reviewer #1)
Comments are mentioned in the attached pdf file. Although a nice work, statistics needs to be reviewed. A little bit of language errors. Histological images needs to be marked with arrows pointing to the damage or change seen.
- Thank you for your encouraging feedback. Based on the comments in PDF file, we have revised the manuscript, including language errors and histological images. We have provided our point-by-point responses to each comment below.

1. Line 18: Modify "were" to "have". In addition, only one author can be the corresponding author.
- Thank you for pointing this out. We have modified the text accordingly in the revised manuscript.
- According to the PLOS ONE's submission guidelines, the following is stated in relation to the corresponding author: “Only one corresponding author can be designated in the submission system, but this does not restrict the number of corresponding authors that may be listed on the article in the event of publication. Whoever is designated as a corresponding author on the title page of the manuscript file will be listed as such upon publication. Include an email address for each corresponding author listed on the title page of the manuscript." Therefore, we kept two corresponding authors in the current study.

2. Line 25-27, 51-52, 80-81: Modify "few" to "a few".
-Thank you for pointing this out. We have modified the text accordingly in the revised manuscript.

3. Line 90 “Animals and Facility”: Mention the grouping of animals in this section. For example, Animals were divided into 5 groups with 11 animals in each group. Group 1 were administered 0.01 ml of substance x for y days and so on. Kindly do this for both the substances.
- Thank you for your comments. As you suggest, we have revised the text accordingly in the revised manuscript.

4. Line 95, 101: Provide weight range of animals
- Thank you for your comments. As you suggest, we have revised the text accordingly in the revised manuscript.

5. Line 101: Females are considered to be more sensitive. It would have been better if female animals were included. It will help in determining if there are any sex related differences.
- Thank you for pointing this out. In the ISO guideline, the following is stated in relation to the intradermal irritation study (#1): “Healthy young adult albino rabbits of either sex from a single strain, weighing not less than 2 kg, shall be used. A minimum of three animals shall initially be used to evaluate the test material. Inject intracutaneously 0,2 ml of the extract obtained with polar or non-polar solvent at five sites on one side of each rabbit.” Since the test substance was administered to five sites on the back skin, we selected a male as a test system, which was expected to have less interference between the administered substances because it is larger than that of female. In the current study, since there was no reaction by the administration of polysorbate 20 in the male SD rats, it is expected that there will be no side effects by the administration of polysorbate 20 in female SD rats.
-#1: Iso B, STANDARD B. Biological evaluation of medical devices. Part. 2009;1: 10993.

6. Were the rats and rabbits placed in the same animal room or different ones? Since the temperatures are different, is it that the mentioned temperatures are required for their survival?
- Thank you for your comments. The four-week repeated intramuscular dose toxicity study in SD rats were conducted in Medytox R&D Center (Republic of Korea) and all SD rats were purchased from Orient Bio, Inc. (Republic of Korea). The intradermal irritation study in NZW rabbits were performed at KTR (Republic of Korea), and all NZW rabbits were purchased from DooYeol Biotech (Republic of Korea). SD rats and NZW rabbits used in these two studies were placed in different facilities. In addition, the overall environment of the facilities for SD rats and NZW rabbits was optimally set based on the guidelines (#1 and #2)

Powered by Editorial Manager® and ProduXion Manager® from Aries Systems Corporation

-#1: ARRP. Guideline 20: Guidelines for the Housing of Rats in Scientific Institutions. Animal Research Review Panel. 2007.
-#2: ARRP. Guideline 18: Guidelines for the Housing of Rabbits in Scientific Institutions. Animal Research Review Panel. 2003

7. Line 178-179: Modify “based on” to “according to”.
- Thank you for pointing this out. We have modified the text accordingly in the revised manuscript.

8. Line 182-183: Delete “using the naked eye”.
- Thank you for pointing this out. We have modified the text accordingly in the revised manuscript.

9. Line 187-188: Modify “Then, all slides were” to “All slides were then” and “hematoxylin & eosin” to “hematoxylin and eosin”. In addition, delete “at KTR”. Avoid mentioning everytime that it was done at KTR. One time mention will suffice.
- Thank you for pointing this out. We have modified the text accordingly in the revised manuscript.

10. Line 227-230 “In SD rats, all substances were injected once every two weeks for four weeks (total three times; days 1, 15, 29) into one site of the gastrocnemius muscle of the right hindlimb.”: What was the basis for selection of these days for dosing. Why not daily as in acute toxicity studies?
- Thank you for your comments. The objective of this study was to verify the in vivo safety properties of polysorbate 20, an excipient of CORETOX® (newly developed BoNT/A product), when administered intramuscularly. Therefore, the dosing schedule (interval and frequency) was set based on the clinical application schedule of BoNT/A products (#1). The clinical application interval of BOTOX® (first FDA-approved BoNT/A product), which is similar to that of CORETOX®, is once every 3 months (Biologic license application number: 103000). In addition, according to the FDA approval document of BOTOX®, a 4-week repeated dose intramuscular toxicity study in rats was performed. Therefore, we have selected the dosing schedule of polysorbate 20 (once every two weeks for four weeks, total three times; days 1, 15, 29) based on clinical application dosing schedule, information of FDA-approved BoNT/A product, and ICH guideline. Furthermore, we thought that this four-week repeated intramuscular dose toxicity study could sufficiently replace the acute toxicity study. Because the dose of polysorbate 20 in the current study (0.02, 0.1, and 0.4 mg/kg) is more than six times the clinical application dose (0.003 ~ 0.06 mg/kg) (#2 and #3) and polysorbate 20 (with CORETOX®) is administered every 3 months (or single dose) in human, as mentioned above. Since the commented part is the figure legend section, we have modified the text accordingly in the method section.
-#1: Guideline ICHHT. Guidance on nonclinical safety studies for the conduct of human clinical trials and marketing authorization for pharmaceuticals M3 (R2). International conference on harmonisation of technical requirements for registration of pharmaceuticals for human use. 2009.
-#2: Lee J, Chun MH, Ko YJ, Lee S-U, Kim DY, Paik N-J, et al. Efficacy and safety of MT10107 (Coretox®) in post-stroke upper limb spasticity treatment: A randomized, double-blind, active drug-controlled, multi-center, phase III clinical trial. Arch Phys Med Rehabil. 2020.
-#3: Kwak S, Kang W, Rhee C-H, Yang G-H, Cruz DJM. Comparative Pharmacodynamics Study of 3 Different Botulinum Toxin Type A Preparations in Mice. Dermatologic Surg. 2020;46: e132–e138.

11. Line 231-232 “In NZW rabbits, all substances were injected once into five sites of the back skin (total one time; day 1).”: What is the basis of dosing selection?
- Thank you for your comments. As mentioned above (Q5), the following is stated in relation to the intradermal irritation study in the ISO guideline (#1): “Inject intracutaneously (intradermally) 0,2 ml of the extract obtained with polar or non-polar solvent at five sites on one side of each rabbit.” In addition, polysorbate 20 (with CORETOX®) is administered every 3 months (or single dose) in human, as mentioned above (Q10). Therefore, we have selected the dosing method of polysorbate 20 based on the ISO guideline and clinical application dosing schedule. Since the commented part is the figure legend section, we have modified the text accordingly in the method section.

Powered by Editorial Manager® and ProduXion Manager® from Aries Systems Corporation

12. Line 235-236, 332, Table 2 : Modify “hematology, clinical chemistry, serum IgE, and serum histamine” to “hematology, clinical chemistry and serum IgE and histamine”. Also, instead of chemistry, I feel the appropriate word used could be clinical biochemistry.
- Thank you for pointing this out. We have modified the text accordingly in the revised manuscript.

13. Line 239-240: Doses have already been mentioned in methods section. Repetition unnecessary. If any changes were observed, then mention the doses on which changes were observed only.
- Thank you for pointing this out. We have modified the text accordingly in the revised manuscript.

14. Line 241, 244: Significanlty higher or lower, please mention. In addition, modify “group administered” to “group that were administered”.
- Thank you for pointing this out. We have modified the text accordingly in the revised manuscript.

15. Line 242, 245, 284, 294, 342, 363: Delete “Vehicle”. Mention in the beginning of the manuscript that control group was administered the vehicle only. And then just mention control in all the places in the manuscript.
- Thank you for pointing this out. We have modified the text accordingly in the revised manuscript.

16. Line 248: If the doses are already mentioned in the methods section, there is no need to mention them again and again. It can be simply mentioned that no changes were seen in all the administered doses. If there is a change, them mention the specific dose at which the change was seen.
- Thank you for pointing this out. We have modified the text accordingly in the revised manuscript.

17. Line 253 and 256: Why arent the data represented as mean=/- standard error of mean? Furthermore, ANOVA or Kruskal-Wallis test are usually followed by a post hoc test such as Dunnet's test, Bonferoni's test or Tukey's test. However, none of the tests have been done to make comparasions. Authors kindly perform a test which best suits your data represented to make comparasions between test and control values and one test and another test values. Remove the shadings, significant values can be represented by a superscript *
- Thank you for pointing this out. The Kruskal-Wallis test performed to compare toxicological indicators in the current study is a ranking-based nonparametric statistical test. Therefore, we presented the median and range in Tables 2 and 3 to show the data without statistical bias. However, since the raw data of the information presented in Tables 2 and 3 are all presented in S3 table, we have modified Tables 2 and 3 (mean ± standard error of the mean, no shadings) accordingly in the revised manuscript. In addition, the Dunn's Rank Sum test was performed as a post hoc test of Kruskal-Wallis test for the significance of intergroup difference between the control and administered groups. We have modified the text accordingly in the method section.

18. Line 257: Modify "in" to "of".
- Thank you for pointing this out. We have modified the text accordingly in the revised manuscript.

19. Line 258: Modify "polysorbate 20 or HSA related macroscopic findings were not observed ~" to " no polyabsorbate 20 or....... macroscopic findings were observed such as changes in external appearance......".
- Thank you for pointing this out. We have modified the text accordingly in the revised manuscript.

20. Line 259-276: Changes observed should be represented as the mean or average changes observed in the whole group, not individual animal changes. Do not mention individual animal changes, rather describe the mean value obtained from the observed group. Don’t mention individual rat results, mention what was most commonly observed. Follow this for all the below results too. Please mention only the most

Powered by Editorial Manager® and ProduXion Manager® from Aries Systems Corporation

common effects seen over all. Please refer some toxicity papers and observe how data are represented.
- Thank you your recommendations. In the current study, there was no significant histopathological change according to the administered substance in SD rats. Therefore, we have deleted individual changes and only represented the change of the whole groups for the concise histopathology results. We have modified the text accordingly in the revised manuscript.

21. Line 267, 364: Modify “administration group” to “administered group”.
- Thank you for pointing this out. We have modified the text accordingly in the revised manuscript.

22. Line 332-334: Mention p value. And Modify “a” to “the”.
- Thank you for pointing this out. We have modified the text accordingly in the revised manuscript.

23. Figure 2: Histological images needs to be marked with arrows pointing to the damage or change seen.
- Thank you for pointing this out. In Figure 2, macrophage infiltration and neovascularization were seen in all administered group (Fig 2a ~ l). In Figure 3, No histopathological findings were seen in all administered groups (Fig 3a ~ f). Therefore, we have modified the Figure 2 and Figure legend 2 accordingly in the revised manuscript.

Response to Reviewers' comments (Reviewer #2)
The paper mentioned the use of polysorbate-20 as a replacement of the HSA in the BoNT/A formulation.
- Thank you for your overall comments, which have substantially improved the manuscript. We have provided our point-by-point responses to each comment below.

1. The study mentioned the use of Polysorbate-20 as an excipient for the BoNT products. But no where in the paper any results mention about presence of Botulinum neurotoxin along with polysorbate-20. According to study results polysorbate-20 is nontoxic excipient, and similar as HSA for the any pharmaceutical products administer through transdermal or intramuscular route. Not just limited for the BoNT. Additionally, Polysorbate-20 is already approved exicipient from FDA, so significance of this study is limited.
- Thank you for your comments. Polysorbate 20 is an inactive ingredient for drug products that have already been approved by the FDA for auricular, intramuscular, intravenous, nasal, ophthalmic, oral, subcutaneous, topical, and vaginal administration (Drug Approvals and Databases). However, since it is mainly used as an excipient in tablet and ophthalmic solutions, most of the safety studies on polysorbate 20 have been conducted on oral toxicity and skin irritation (#1 and 2). There are few studies on the safety of polysorbate 20 when administered intramuscularly. Therefore, the current study was conducted to obtain in vivo safety data for intramuscular administration of polysorbate 20, which has little data currently accumulated. Findings of the current study suggest that polysorbate 20 could be used as a safe excipient for various pharmaceuticals administered intramuscularly. In addition, BoNT/A product (CORETOX®) that uses polysorbate 20 as an excipient have already been approved by the Ministry of Food and Drug Safety (Republic of Korea) and are on the market. In the approval process, in vivo safety and efficacy studies were already conducted in the presence of Botulinum neurotoxin along with polysorbate 20 and the relevant results were submitted to regulatory authorities. Therefore, in the current study, the safety of Botulinum neurotoxin along with polysorbate 20 was not investigated.
-#1: Moore J. Final report on the safety assessment of polysorbates 20, 21, 40, 60, 61, 65, 80, 81, and 85. J Am Coll Toxicol. 1984;3: 1–82.
-#2: Lanigan RS. Final report on the safety assessment of PEG-20 sorbitan cocoate; PEG-40 sorbitan diisostearate; PEG-2,-5, and-20 sorbitan isostearate; PEG-40 and-75 sorbitan lanolate; PEG-10,-40,-44,-75, and-80 sorbitan laurate; PEG-3, and-6 sorbitan oleate; PEG-80 sorb. Int J Toxicol. 2000;19: 43–89.

2. The title as well the introduction should be rewritten without emphasizing the Botulinum toxin. The current data seems the controls used for the formulation study. However, authors can include the possible use of polysorbated-20 as an excipient for

	Botulinum toxin products in the discussion but should not be only therapeutic product.<br>- Thank you for your recommendation. As you suggest, we have revised the title to "Safety verification for polysorbate 20, pharmaceutical excipient for intramuscular administration, in Sprague-Dawley rats and New Zealand White rabbits". Throughout the 'Introduction' part, we have rewritten the manuscript to emphasize polysorbate 20 and HSA rather than Botulinum toxin. In addition, in 'Introduction' and 'Conclusions' part, we have revised the manuscript to emphasize that the findings of the current study could be applied to a various therapeutic product, not limited to BoNT/A product.<br><br>3. To use the polysorbated-20 as an excipient in the BoNT based formulation required in depth efficacy, safety, pharmacokinetics and pharmacodynamic study of the Botulinum toxin in the presence of polysorbate-20. The data should be compared with the existing formulation like Botox or dysport etc. The study having BoNT formulation with Polysorbate-20 has already published and mentioned in the paper as a reference # 18 and 19. So the significance of this particular control study reduces.<br>- Thank you for your comments. As mentioned in the answer to Q2, in vivo safety, efficacy, pharmacodynamic studies were already conducted in the presence of Botulinum neurotoxin along with polysorbate 20 and the relevant results were submitted to regulatory authorities. Considering the characteristics of Botulinum toxin (local diffusion at the injection site, followed by rapid systemic metabolism and excretion), pharmacokinetic studies were not conducted. In those studies, the efficacy and toxicity of BOTOX® (uses HSA as an excipient) and CORETOX® (uses polysorbate 20 as an excipient) were also compared. In addition, since polysorbate 20 is an inactive ingredient, it is considered that efficacy, pharmacokinetics, and pharmacodynamic studies other than safety study are unnecessary when used alone. Therefore, the current study was conducted to obtain in vivo safety data of polysorbate 20. Although polysorbate 20 have already been approved by the FDA for the pharmaceutical excipient, the current study is considered worthwhile given the lack of safety data on intramuscular administration of polysorbate 20 (#1 and #2). It is expected that findings of the current study provides the basis for the in vivo safety profile of polysorbate 20 administered intramuscularly and the scientific reliability of the use of polysorbate 20 as an excipient for various pharmaceuticals in terms of its safety.<br>-#1: Moore J. Final report on the safety assessment of polysorbates 20, 21, 40, 60, 61, 65, 80, 81, and 85. J Am Coll Toxicol. 1984;3: 1–82.<br>-#2: Lanigan RS. Final report on the safety assessment of PEG-20 sorbitan cocoate; PEG-40 sorbitan diisostearate; PEG-2,-5, and-20 sorbitan isostearate; PEG-40 and-75 sorbitan lanolate; PEG-10,-40,-44,-75, and-80 sorbitan laurate; PEG-3, and-6 sorbitan oleate; PEG-80 sorb. Int J Toxicol. 2000;19: 43–89.<br><br>4. Data table 2 and 3 title are same. Please specify if table 2 belong to polysorbate-20 or for HSA.<br>- Thank you for your comments. Table 2 shows the level of hematology, clinical biochemistry, and serum IgE and histamine in male SD rats, while Table 3 shows the same values as table 2 in female SD rats.
**Additional Information:**	
**Question**	**Response**
**Financial Disclosure**<br><br>Enter a financial disclosure statement that describes the sources of funding for the work included in this submission. Review the submission guidelines for detailed requirements. View published research articles from *PLOS ONE* for specific examples.<br><br>This statement is required for submission and **will appear in the published article** if	This study was supported by the Technology Innovation Program, establishment of risk management platform with aim of reduce attrition of new drugs and its service [grant number: 10067737, 2016], funded by the Ministry of Trade, Industry & Energy in Republic of Korea (WCS).<br>The funders had no role in study design, data collection and analysis, decision to publish, or preparation of the manuscript.

the submission is accepted. Please make sure it is accurate.<br><br>**Unfunded studies**<br>Enter: *The author(s) received no specific funding for this work.*<br><br>**Funded studies**<br>Enter a statement with the following details:<br>• Initials of the authors who received each award<br>• Grant numbers awarded to each author<br>• The full name of each funder<br>• URL of each funder website<br>• Did the sponsors or funders play any role in the study design, data collection and analysis, decision to publish, or preparation of the manuscript?<br>• **NO** - Include this sentence at the end of your statement: *The funders had no role in study design, data collection and analysis, decision to publish, or preparation of the manuscript.*<br>• **YES** - Specify the role(s) played.<br><br>* typeset	
**Competing Interests**<br><br>Use the instructions below to enter a competing interest statement for this submission. On behalf of all authors, disclose any competing interests that could be perceived to bias this work—acknowledging all financial support and any other relevant financial or non-financial competing interests.<br><br>This statement is required for submission and **will appear in the published article** if the submission is accepted. Please make sure it is accurate and that any funding sources listed in your Funding Information later in the submission form are also declared in your Financial Disclosure statement.<br><br>View published research articles from *PLOS ONE* for specific examples.	The authors have declared that no competing interests exist.

**NO authors have competing interests**<br><br>Enter: *The authors have declared that no competing interests exist.*<br><br>**Authors with competing interests**<br><br>Enter competing interest details beginning with this statement:<br><br>*I have read the journal's policy and the authors of this manuscript have the following competing interests: [insert competing interests here]*<br><br>* typeset	
**Ethics Statement**<br><br>Enter an ethics statement for this submission. This statement is required if the study involved:<br><br>• Human participants<br>• Human specimens or tissue<br>• Vertebrate animals or cephalopods<br>• Vertebrate embryos or tissues<br>• Field research<br><br>Write "N/A" if the submission does not require an ethics statement.<br><br>General guidance is provided below. Consult the submission guidelines for detailed instructions. **Make sure that all information entered here is included in the Methods section of the manuscript.**	All studies complied with the Animal Protection Act (No. 13023) and Laboratory animal Act (No. 15944). The four-week repeated intramuscular dose toxicity study was reviewed and approved by the Institutional Animal Care and Use Committee of Medytox, Inc. (A-2019-009). The intradermal irritation study was reviewed and approved by the Institutional Animal Care and Use Committee of Korea Testing & Research Institute (KTR, IAC2019-1500).<br>All Sprague-Dawley rats were euthanized by exsanguination from the posterior vena cava and abdominal aorta under isoflurane anesthesia, while all New Zealand White rabbits were euthanized by intravenous injection of T-61 solution.

Powered by Editorial Manager® and ProduXion Manager® from Aries Systems Corporation

**Format for specific study types**<br><br>**Human Subject Research (involving human participants and/or tissue)**<br>• Give the name of the institutional review board or ethics committee that approved the study<br>• Include the approval number and/or a statement indicating approval of this research<br>• Indicate the form of consent obtained (written/oral) or the reason that consent was not obtained (e.g. the data were analyzed anonymously)<br><br>**Animal Research (involving vertebrate animals, embryos or tissues)**<br>• Provide the name of the Institutional Animal Care and Use Committee (IACUC) or other relevant ethics board that reviewed the study protocol, and indicate whether they approved this research or granted a formal waiver of ethical approval<br>• Include an approval number if one was obtained<br>• If the study involved *non-human primates*, add *additional details* about animal welfare and steps taken to ameliorate suffering<br>• If anesthesia, euthanasia, or any kind of animal sacrifice is part of the study, include briefly which substances and/or methods were applied<br><br>**Field Research**<br><br>Include the following details if this study involves the collection of plant, animal, or other materials from a natural setting:<br>• Field permit number<br>• Name of the institution or relevant body that granted permission	
**Data Availability**<br><br>Authors are required to make all data underlying the findings described fully available, without restriction, and from the time of publication. PLOS allows rare exceptions to address legal and ethical concerns. See the PLOS Data Policy and FAQ for detailed information.	Yes - all data are fully available without restriction

Powered by Editorial Manager® and ProduXion Manager® from Aries Systems Corporation

A Data Availability Statement describing where the data can be found is required at submission. Your answers to this question constitute the Data Availability Statement and **will be published in the article**, if accepted.<br><br>**Important:** Stating 'data available on request from the author' is not sufficient. If your data are only available upon request, select 'No' for the first question and explain your exceptional situation in the text box.<br><br>Do the authors confirm that all data underlying the findings described in their manuscript are fully available without restriction?	
**Describe where the data may be found in full sentences. If you are copying our sample text, replace any instances of XXX with the appropriate details.**<br><br>• If the data are **held or will be held in a public repository**, include URLs, accession numbers or DOIs. If this information will only be available after acceptance, indicate this by ticking the box below. For example: *All XXX files are available from the XXX database (accession number(s) XXX, XXX.).*<br>• If the data are all contained **within the manuscript and/or Supporting Information files**, enter the following: *All relevant data are within the manuscript and its Supporting Information files.*<br>• If neither of these applies but you are able to provide **details of access elsewhere**, with or without limitations, please do so. For example:<br><br>*Data cannot be shared publicly because of [XXX]. Data are available from the XXX Institutional Data Access / Ethics Committee (contact via XXX) for researchers who meet the criteria for access to confidential data.*<br><br>*The data underlying the results presented in the study are available from (include the name of the third party*	All relevant data are within the manuscript and its Supporting Information files.

and contact information or URL).<br>• This text is appropriate if the data are owned by a third party and authors do not have permission to share the data.<br><br>* typeset	
Additional data availability information:	

Powered by Editorial Manager® and ProduXion Manager® from Aries Systems Corporation

Manuscript Click here to access/download;Manuscript;Manuscript.doc

Safety verification for polysorbate 20, pharmaceutical excipient for intramuscular administration, in Sprague-Dawley rats and New Zealand White rabbits

Short title: *In vivo* safety evaluation of Polysorbate 20

Junhyung Kim[1¶], Seongsung Kwak[1,4¶], Mi-Sun Park[1], Chang-Hoon Rhee[2], Gi-Hyeok Yang[1], Jangmi Lee[3], Woo-Chan Son[4&*], and Won-ho Kang[1&*]

[1]Gwanggyo R&D Center, Medytox Inc., Suwon-si, Gyeonggi-do, Korea

[2]Osong R&D Center, Medytox Inc., Cheongju-si, Chungcheongbuk-do, Korea

[3]Department of Medical Science, Asan Medical Institute of Convergence Science and Technology, Asan Medical Center, University of Ulsan College of Medicine, Songpa-gu, Seoul, Korea

[4]Department of Pathology, Asan Medical Center, Songpa-gu, Seoul, Korea

*Corresponding author

E-mail: whkang@medytox.com (WhK)

E-mail: wcson32@hanmail.net (WCS)

[¶]These authors contributed equally to this work as first author.

[&]These authors contributed equally to this work as corresponding author.

Abstract

Human serum albumin (HSA) has been widely used as a pharmaceutical excipient in Botulinum toxin serotype A (BoNT/A) products that are indicated for use in therapeutics and cosmetics. However, HSA as a human-derived material has some concerns, such as the potential risk of transmission of infectious agents, an insufficient supply, and difficulty in maintaining a certain quality. For those reasons, newly developed BoNT/A products (CORETOX®, Medytox, Inc., Republic of Korea) contained polysorbate 20, a non-human-derived excipient, to replace the HSA. However, most safety studies of polysorbate 20 have been conducted with non-invasive routes of administration, and thus there are a few studies on the safety of polysorbate 20 when administered intramuscularly. To secure the *in vivo* safety profile of polysorbate 20, a four-week repeated intramuscular dose toxicity study (0.02, 0.1, and 0.4 mg/kg, one injection every two weeks for a total of three injections) was conducted in 66 Sprague-Dawley (SD) rats. Then, an intradermal irritation study was further conducted with 18 New Zealand White (NZW) rabbits. The toxicological evaluation of HSA (0.06 and 0.12 mg/kg) was also carried out as a comparative substance. Systemic and local toxicities were not observed in any of the SD rats or NZW rabbits based on clinical signs, body weight, hematology, clinical biochemistry, macroscopic findings on necropsy, histopathology of the injection site, and allergic reactions. The current study suggested that intramuscular administration of polysorbate 20 was considered to be safe at a level similar to that of HSA, which has an *in vivo* safety profile accumulated over the years. This provided the basis for the *in vivo* safety profile of polysorbate 20 administered intramuscularly and the scientific reliability of the use of polysorbate 20 as an alternative to HSA, which is used as an excipient for various pharmaceuticals in terms of its safety.

Introduction

Human serum albumin (HSA), the most abundant protein in human plasma, has the functions of regulating the colloid osmotic pressure, binding to and transporting various molecules, antioxidant action, anticoagulant action, regulating membrane permeability, and a direct neuroprotective action [1,2]. Therefore, HSA has been used for therapeutic purposes in patients with hemorrhage, hypovolemia, and hypoalbuminemia since about the 19th century [2]. In addition to therapeutic purposes, it is used as an excipient for various pharmaceuticals in biomedical aspects, [1,3].

Most botulinum toxin serotype A (BoNT/A) products that are indicated for use in therapeutics and cosmetics also use HSA as an excipient [4,5]. In BoNT/A products, HSA plays a role in stabilizing the neurotoxic proteins during manufacturing, transportation, and administration and in preventing the aggregation of neurotoxic proteins [6]. However, there are several concerns with HSA, although it is produced under strict regulations and has a safety profiling accumulated over the years [2,6]. Since the production of HSA mainly relies on human blood donation, the quality of HSA could differ depending on the population of the human blood donors (race or geographical distribution) [7]. Furthermore, the market demand for HSA has dramatically increased (over 500 tons), and it is one of the most widely used biopharmaceutical solutions today [7,8]. This could lead to a worldwide shortage of HSA in the market [2]. Finally, the use of HSA has a theoretical risk of spreading blood-derived pathogens [6,9]. Currently, the transmission of viral diseases, such as hepatitis B virus, human immunodeficiency virus, and West Nile virus are well controlled through strict management of HSA, but there are still concerns about the transmission of prion-derived variant Creutzfeldt-Jakob disease [10,11].

For those reasons, Medytox, Inc. (Republic of Korea) has recently developed a

complexing protein-free BoNT/A product (CORETOX®) that uses polysorbate 20, a non-human-derived excipient, to replace the HSA [12,13]. Polysorbates, a series of polyoxyethylenated sorbitan esters, are hydrophilic and nonionic surfactants that have been widely used as a food additive and stabilizer in pharmaceuticals [14,15]. Among them, polysorbate 20, called polyoxyethylene-20-sorbitan monolaurate, has 20 repeating units of polyethylene glycol. It is mainly used as a direct/indirect additive in food and emulsifiers/surfactants in cosmetics that come into direct contact with the human skin, such as shampoo, bath soap, skin cleansing products, and hair spray [15]. It has also been used as an excipient in pharmaceuticals as a stabilizer, particularly in tablet and ophthalmic solutions. Hence, most of the safety studies on polysorbate 20 have been conducted on oral toxicity and skin irritation [14]. As a result, it was found that excessive dose of polysorbate20 could induce mild/temporary eye irritation and skin irritation [14–17]. In addition, when administered orally, it adversely affects the gastro-intestinal tract and, in severe cases, could cause death [14,16]. However, there are a few studies on the safety of polysorbate 20 administered by other routes, especially intramuscular administration, which is the route of BoNT/A injection.

The objective of this study was to verify the *in vivo* safety properties of polysorbate 20 when administered intramuscularly. First, a four-week repeated intramuscular dose toxicity study in Sprague-Dawley (SD) rats (one injection every two weeks for a total of three injections) was carried out to evaluate its systemic toxicity. Second, an intradermal irritation study in New Zealand White (NZW) rabbits was carried out to evaluate local toxicity and allergic reactions. It was expected the current study could enable the evaluation of the safety of polysorbate 20 for intramuscular administration and would provide the basis for the *in vivo* safety profile of polysorbate 20 as a pharmaceutical excipient.

Materials and methods

Animals and facility

This study was reviewed and approved by the Institutional Animal Care and Use Committee of Medytox, Inc. (A-2019-009) and the Korea Testing & Research Institute (KTR, IAC2019-1500). In addition, the study design was established based on the ARRIVE guidelines [18].

In the four-week repeated intramuscular dose toxicity study performed at Medytox R&D Center (Republic of Korea), a total of 66 SD rats, consisting of 30 females (130.7 ~ 146.2g) and 36 males (163.5 ~ 187.2g), were used (Table 1). Healthy five-week-old SD rats were purchased from Orient Bio, Inc. (Republic of Korea). Upon receipt, all animals were macroscopically examined and acclimated for 7 days to the laboratory conditions (a specific pathogen-free animal room; temperature, 23 ± 3°C; humidity, 55 ± 15%; ventilation, 15 air changes per hour; light/dark cycle, 12 hours; light intensity, 150 ~ 300 Lux; polycarbonate cage; 2 ~ 3 animals per cage). Then, animals were divided into six groups with 11 animals (six male and five female SD rats) in each group. Group 1 (control group) were administered 0.9% saline every two weeks. Group 2, 3, and 4 were administered 0.02, 0.1, and 0.4 mg/kg of polysorbate 20 every two weeks, respectively. Group 5 and 6 were administered 0.06 and 0.12 mg/kg of HSA every two weeks, respectively.

In the intradermal irritation study performed at KTR (Republic of Korea), a total of 18 male NZW rabbits (2307.7 ~ 2947.8 g) were used (Table 1). Healthy three-month-old rabbits were purchased from DooYeol Biotech (Republic of Korea). Upon receipt, all animals were macroscopically examined and acclimated for 6 days to the laboratory conditions (specific pathogen-free animal room; temperature, 20.3 ~ 21.9°C; humidity, 54.5 ~ 64.9%; ventilation, 10~ 20 air changes per hour; light/dark cycle, 12 hours; light intensity, 150 ~ 300 Lux; stainless steel

cage; 1 animal per cage). Then, animals were divided into six groups with 3 male NZW rabbits in each group. Group 1 (control group) were administered 0.9% saline once. Group 2, 3, and 4 were administered 0.02, 0.1, and 0.4 mg/kg/site of polysorbate 20 once, respectively. Group 5 and 6 were administered 0.06 and 0.12 mg/kg/site of HSA once, respectively.

Table 1. Study design of four-week repeated intramuscular dose toxicity study in Sprague-Dawley rats and intradermal irritation study in New Zealand white rabbits

Study	Species	Group	Substance	Dose volume	Dose	Route and Frequency of administration	No. animals (Male/Female)
**Four-week repeated intramuscular dose toxicity study**	**Sprague-Dawley Rats**	G1	Control (0.9 % saline)	0.2 mL/kg	0 mg/kg	Intramuscular injection (Gastrocnemius muscle) Once per 2 weeks (Total 3 times)	11 (6/5)
		G2	Polysorbate 20		0.02 mg/kg		11 (6/5)
		G3	Polysorbate 20		0.1 mg/kg		11 (6/5)
		G4	Polysorbate 20		0.4 mg/kg		11 (6/5)
		G5	Human serum albumin		0.06 mg/kg		11 (6/5)
		G6	Human serum albumin		0.12 mg/kg		11 (6/5)
		**Total**					**66 (36/30)**
**Intradermal irritation study**	**New Zealand White rabbits**	G1	Control (0.9 % saline)	0.4 mL/site (Total 5 sites)	0 mg/kg/site	Intradermal injection (Dorsal back skin) Once (Total 1 time)	3 (3/0)
		G2	Polysorbate 20		0.02 mg/kg/site		3 (3/0)
		G3	Polysorbate 20		0.1 mg/kg/site		3 (3/0)
		G4	Polysorbate 20		0.4 mg/kg/site		3 (3/0)
		G5	Human serum albumin		0.06 mg/kg/site		3 (3/0)
		G6	Human serum albumin		0.12 mg/kg/site		3 (3/0)
		**Total**					**18 (18/0)**

Vehicle, positive control substance, and test substance

Saline (0.9% NaCl, DAI HAN PHARM. Co., Republic of Korea) was used as the vehicle in this study conducted in SD rats and NZW rabbits. HSA (Green Cross Corp., Republic of Korea) was selected as the comparative substance for the polysorbate 20 (Merck, Germany) that was the test substance of the current study. In the four-week repeated intramuscular dose toxicity study, the dose of HSA was calculated by multiplying the animal equivalent dose calculation factor (6.2) and the content of HSA in most BoNT/A products, 0.01 and 0.02 mg/kg (0.5 and 1.0 mg/vial, 50 kg human) [4,19]. As a result, 0.06 and 0.12 mg/kg were selected as the dose of HSA. The dose of polysorbate 20 was selected based on the content of polysorbate 20 in CORETOX® (1.0 mg/vial, 60 kg human) and the human dosage of CORETOX® (0.2 ~ 3.6 vial) [12,13]. Then, the dose of polysorbate 20 was also calculated in the same manner as when determining the dose of HSA. As a result, 0.02, 0.1, and 0.4 mg/kg were selected as the dose of polysorbate 20.

The same dose of HSA and polysorbate 20 used in the four-week repeated intramuscular dose toxicity study was applied in the intradermal irritation study in NZW rabbits. Detailed information is provided in Table 1.

In vivo administration

In the four-week repeated intramuscular dose toxicity study in SD rats, all substances (saline, HSA, polysorbate 20) were administered intramuscularly. The dosing formulation was prepared on the day of administration using saline (HSA: 0.3, 0.6 mg/mL; polysorbate 20: 0.1, 0.5, 2.0 mg/mL). The dose volume was selected as 0.2 mL/kg and the individual dose volume for each animal was calculated based on the body weight of the animal on the day of administration [20]. All substances were injected once every two weeks for four weeks (a total of 3 times; days 1, 15, 29) into one site of the gastrocnemius muscle of the right hindlimb. The dosing schedule

(interval and frequency) was set based on the clinical application schedule (total 1 time, at least three month dosing interval) and the administration site (gastrocnemius muscle) was selected based on the site generally used to evaluate the effectiveness of BoNT/A in rodents [12,21–23].

In the intradermal irritation study in NZW rabbits, all substances were administered via the intradermal route. The dosing formulation was prepared on the day of administration using saline (HSA: 0.15, 0.3 mg/mL; polysorbate 20: 0.05, 0.25, 1.0 mg/mL). The dose volume was selected as 0.4 mL/kg/site (total 2 mL/kg/head) and the individual dose volume for each animal was calculated based on the body weight of animal on the day of administration [20]. Before administration, the back skin of the rabbit was depilated of hair. Then, all substances were injected once into five sites (three left side and two right side) on the back skin (total 1 time; day 1) based on the guideline of the International Organization for Standardization (ISO) [24].

Clinical observation and body weights measurement

In the four-week repeated intramuscular dose toxicity study in SD rats, all animals were observed for clinical signs and general condition once daily throughout the study period (days 1 ~ 29). Their body weights were measured once a week (total five times), including the day of administration and at autopsy.

In the intradermal irritation study in NZW rabbits, clinical observation was conducted once daily throughout the study period (days 1 ~ 29). Furthermore, skin reactions at the administration site, including the degree of erythema, crust formation, and edema formation, were observed once daily based on the scoring system for skin reactions of the ISO [24]. Body weights were measured once a week (total five times), including on the day of administration and at autopsy.

Hematology, clinical biochemistry, and allergic reaction test

All animals (SD rats and NZW rabbits) were fasted overnight before the last day of the study. Then, the SD rats were anesthetized with isoflurane and blood samples were collected from their abdominal vein. In the NZW rabbits, blood samples were collected from the marginal ear vein without anesthesia.

Approximately 1 mL of the blood sample of all animals was put into a tube containing EDTA. After that, the following parameters were analyzed in KTR using a hematological autoanalyzer (ADVIA 120, SIEMENS, Germany): white blood cell count (WBC), differential leucocyte count (neutrophils; lymphocytes; monocytes; eosinophils; basophils), red blood cell count (RBC), hemoglobin (HGB), hematocrit (HCT), RBC indices (mean corpuscular volume, MCV; mean corpuscular hemoglobin, MCH; mean corpuscular hemoglobin concentration, MCHC), reticulocyte, and platelets.

The blood samples (except those used in the hematological analysis) were centrifuged at 3,000 rpm for 10 minutes to obtain the serum. Then, the following parameters were examined in KTR using an automatic analyzer (TBA-120FR, TOSHIBA, Japan): total protein, albumin, albumin/globulin ratio, total bilirubin, total bile acid, alkaline phosphatase (ALP), aspartate aminotransferase (AST), aspartate aminotransferase (AST), alanine aminotransferase (ALT), gamma glutamyltransferase (GGT), creatinine, blood urea nitrogen (BUN), total cholesterol, triglycerides (TG), glucose, calcium, inorganic phosphorus, creatine phosphokinase, cholinesterase, sodium, potassium, and chloride. Among these, total bile acid and cholinesterase were only analyzed for the NZW rabbits.

In addition, the levels of serum IgE and histamine of all animals were measured using an

enzyme-linked immunosorbent assay (ELISA) kit.

Necropsy and histopathology

After blood sampling from the SD rats and NZW rabbits, autopsies were performed on all animals at day 29 according to the AVMA Guidelines for the Euthanasia of Animals [25]. All SD rats were euthanized by exsanguination from the posterior vena cava and abdominal aorta under isoflurane anesthesia. All NZW rabbits were euthanized by intravenous injection of T-61 solution (MSD animal health, Republic of Korea). At necropsy, their external appearance, internal organs, and injection sites were observed macroscopically. Then, the injection site (one site of the gastrocnemius muscle of the right hindlimb for the SD rats and the five sites of the back skin for the NZW rabbits) were collected and stored in 10% neutral buffered formalin. Slides of all preserved tissues for histopathology were prepared through general tissue processing, including dehydration and paraffin embedding. All slides were then stained with hematoxylin and eosin and examined microscopically.

Statistical analysis

The Kruskal-Wallis test was performed to compare toxicological indicators, including body weights, hematology level, clinical biochemistry level, and serum IgE and histamine levels among the groups of SD rats and NZW rabbits. In the case of body weight, since there was a difference in the mean body weight among the groups before the first dose administration, the statistical analysis was performed using the ratio of body weight at the time of the measurement to the body weight before the first administration. The significance of intergroup difference

between the control and administered groups was assessed using Dunn's Rank Sum test. All statistical analyses were performed using GraphPad Prism 8.0 (GraphPad Software, Inc., San Diego, CA) and a *p*-value of < 0.05 was accepted to indicate statistical significance.

Results

Mortality and clinical Signs

In the four-week repeated intramuscular dose toxicity study, all SD rats remained well throughout the study and showed no clinical signs of disease, pain or distress. In the intradermal irritation study, no deaths or abnormal findings were observed in all NZW rabbits. Skin reactions, including erythema, eschar formation, and edema formation, were not observed regardless of the administered substance.

Changes in body weights

For both the SD rats and NZW rabbits, their body weight was measured once a week for a total of five times. Individual body weights of all animals by time point are presented in the S1 and S2 Tables.

In the four-week repeated intramuscular dose toxicity study, a gradual body weight gain of the SD rats was observed regardless of the administered substance (Fig 1a). Compared to the body weight before the first administration, all male SD rats had a body weight of 138.4% at day 8, 173.6% at day 15, 200.4% at day 22, and 225.0% at day 29, respectively, while all female SD rats had a body weight of 132.2% at day 8, 153.1% at day 15, 173.1% at day 22, and 187.3% at

day 29, respectively. There was no significant increase or decrease of the body weight of the SD rats administered polysorbate 20 and HSA compared to the control group at any of the time points.

In the intradermal irritation study, a gradual body weight gain of the NZW rabbits was also observed regardless of the administered substance (Fig 1b). Compared to their body weight before administration, all NZW rabbits had a weight of 105.0% at day 8, 111.0% at day 15, 116.7% at day 22, and 120.8% at day 29. There was also no significant difference in body weight of the NZW rabbits administered polysorbate 20 and HSA compared to the control group at any of the time points.

Fig 1. Body weight changes in a four-week repeated intramuscular dose toxicity study in Sprague-Dawley (SD) rats and an intradermal irritation study in New Zealand White (NZW) rabbits. Body weight changes measured once a week in a) SD rats and b) NZW rabbits, for a total of five times during the study period. In SD rats, all substances were injected once every two weeks for four weeks (total three times; days 1, 15, 29) into one site of the gastrocnemius muscle of the right hindlimb. Neither male nor female rat groups showed significant differences in body weight on days 8, 15, 22, and 29 (Kruskal-Wallis test). In NZW rabbits, all substances were injected once into five sites of the back skin (total one time; day 1). There was no significant difference in body weight on days 8, 15, 22, and 29 in all groups (Kruskal-Wallis test).

Findings of the level of hematology, clinical biochemistry, and serum IgE and histamine

Hematological tests and clinical biochemistry tests were performed on all SD rats and NZW rabbits. Individual results are presented in the S3 and S4 Tables. In both male and female SD rats, there were no polysorbate 20 or HSA related changes in the hematology parameters. In clinical biochemistry, compared to the control group, the albumin/globulin ratio was significantly decreased in male group 6 ($p < 0.05$), the level of glucose was significantly decreased in male group 3, 5, and 6 ($p < 0.05$, respectively), and the level of sodium was significantly increased in male group 4 ($p < 0.01$) and 6 ($p < 0.05$) (Table 2). In females, the level of total bilirubin was significantly decreased in group 2 and 4 ($p < 0.05$, respectively) and the level of chloride was significantly increased in group 2 ($p < 0.05$) and 3 ($p < 0.01$), compared to the control group (Table 3). Furthermore, in the allergic reaction test (ELISA analysis of serum IgE and histamine), significantly higher serum histamine levels were found in the female group 5 and 6 compared to the control group ($p < 0.05$).

In NZW rabbits, there were no changes in the hematology and clinical biochemistry parameters in the polysorbate 20 or HSA administered animals. There was also no significant increase or decrease in the level of serum IgE and histamine in NZW rabbits administered polysorbate 20 and HSA compared to the control group.

Table 2. The level of hematology, clinical biochemistry, and serum IgE and histamine in male Sprague-Dawley (SD) rats in the current study.

Category		Male					
		**G1**	**G2**	**G3**	**G4**	**G5**	**G6**
**Hematological findings**	**RBC (10^6cells/μL)**	7.37±0.13	7.38±0.14	7.31±0.11	7.48±0.14	7.50±0.10	7.37±0.14
	**HGB (g/dL)**	14.52±0.20	14.57±0.22	14.57±0.31	14.58±0.23	14.7±0.12	14.37±0.24
	**HCT (%)**	46.30±0.40	46.15±0.68	45.87±0.85	46.22±0.57	46.42±0.36	45.67±0.89
	**MCV (fL)**	62.95±0.81	62.57±0.55	62.73±0.56	61.82±0.57	61.95±1.00	62.02±0.09
	**MCH (pg)**	19.72±0.27	19.73±0.16	19.90±0.17	19.50±0.31	19.57±0.27	19.50±0.13
	**MCHC (g/dL)**	31.33±0.19	31.60±0.07	31.72±0.24	31.55±0.23	31.62±0.12	31.48±0.19
	**RETIC (10^9cells/μL)**	218.7±8.4	213.2±9.4	225.7±11.4	238.7±4.2	198.7±11.3	222.8±10.0
	**PLT (10^3 cells/μL)**	1156±63	1168±55	1161±66	1210±39	1228±28	1170±47
	**WBC (10^3 cells/μL)**	10.81±1.14	10.90±0.81	9.68±0.95	8.56±0.44	7.15±0.76	8.14±0.87
**Clinical biochemical findings**	**TP (g/dL)**	5.90±0.06	6.07±0.08	5.97±0.11	6.00±0.09	6.10±0.09	6.13±0.10
	**ALB (g/dL)**	3.85±0.04	3.88±0.03	3.83±0.06	3.85±0.03	3.97±0.04	3.88±0.06
	**A/G ratio**	1.88±0.04	1.78±0.03	1.78±0.04	1.80±0.04	1.87±0.04	1.73±0.02*
	**T-BIL (mg/dL)**	0.04±0.00	0.05±0.00	0.04±0.00	0.03±0.00	0.03±0.00	0.05±0.00
	**ALP (U/L)**	596.3±33.6	711.0±57.2	532.3±38.5	577.2±30.6	560.2±35.6	647.5±47.3
	**AST (U/L)**	90.33±6.35	119.2±30.2	92.67±4.34	81.00±4.46	106.5±4.8	101.5±9.7
	**ALT (U/L)**	28.33±3.46	45.50±16.37	27.17±2.85	22.83±1.56	29.00±2.66	26.17±2.44
	**CREA (mg/dL)**	0.44±0.01	0.45±0.02	0.46±0.02	0.44±0.02	0.40±0.01	0.42±0.01
	**BUN (mg/dL)**	11.78±0.60	12.60±1.00	11.93±0.54	10.42±0.74	11.17±0.48	10.90±0.41
	**T-CHO (mg/dL)**	71.00±3.39	78.50±4.65	76.33±4.26	72.33±5.33	65.83±5.68	82.00±3.01
	**TG (mg/dL)**	80.33±7.06	99.67±13.84	112.5±15.3	96.83±13.26	81.33±9.54	93.67±17.89
	**GLU (mg/dL)**	127.7±2.9	111.0±4.2	103.5±3.7*	117.0±7.9	99.33±3.33*	99.50±6.82*
	**CA (mg/dL)**	10.15±0.11	10.23±0.05	10.13±0.10	10.32±0.06	10.00±0.10	10.23±0.14
	**IP (mg/dL)**	8.90±0.18	8.68±0.17	8.63±0.15	8.78±0.13	9.02±0.09	8.90±0.11
	**GGT (IU/L)**	0.39±0.16	0.34±0.18	0.69±0.26	0.34±0.16	0.19±0.09	0.48±0.26
	**CK (U/L)**	529.7±45.1	529.7±29.3	578.5±60.3	452.5±39.0	745.5±50.4	652.3±79.4
	**Na (mmol/L)**	143.0±0.3	144.4±0.5	143.6±0.3	145.2±0.4**	144.3±0.4	144.9±0.4*
	**K (mmol/L)**	4.68±0.08	4.68±0.06	4.76±0.08	4.87±0.12	4.95±0.05	4.89±0.10
	**Cl (mmol/L)**	101.4±0.3	102.8±0.5	101.4±0.8	103.1±0.5	101.5±0.6	102.3±0.6
**ELISA analysis**	**IgE (ng/mL)**	0.233±0.014	0.270±0.001	0.246±0.015	0.181±0.008	0.200±0.016	0.188±0.004
	**Histamine (ng/mL)**	0.480±0.040	0.383±0.038	0.402±0.034	0.423±0.026	0.519±0.052	0.390±0.040

Data were presented as mean ± standard error of the mean.

All data were analyzed using Kruskal-Wallis test and the significance of intergroup difference between the control and administered groups was

assessed using Dunn's Rank Sum test. All statistical analyses were performed using GraphPad Prism 8.0 (GraphPad Software, Inc., San Diego, CA).

*vs. Control group: $p < 0.05$, ** vs. Control group: $p < 0.01$

Table 3. The level of hematology, clinical biochemistry, and serum IgE and histamine in female Sprague-Dawley (SD) rats in the current study.

Category		Female					
		G1	G2	G3	G4	G5	G6
**Hematological findings**	**RBC (10^6cells/μL)**	7.14±0.20	6.72±0.17	7.06±0.08	7.30±0.09	7.47±0.22	7.30±0.13
	**HGB (g/dL)**	14.08±0.30	13.78±0.31	13.96±0.20	14.58±0.12	14.74±0.28	14.52±0.28
	**HCT (%)**	42.54±0.88	42.08±1.01	42.06±0.46	44.42±0.16	44.60±0.88	44.40±0.76
	**MCV (fL)**	59.70±0.89	62.70±1.13	59.60±0.91	60.92±0.69	59.84±0.92	60.84±0.60
	**MCH (pg)**	19.76±0.22	20.46±0.06	19.78±0.36	20.00±0.26	19.76±0.29	19.88±0.19
	**MCHC (g/dL)**	33.12±0.27	32.70±0.50	33.20±0.15	32.84±0.17	33.00±0.28	32.70±0.15
	**RETIC (10^9cells/μL)**	156.9±10.2	170.9±16.1	151.9±14.7	169.0±5.2	143.1±13.7	159.8±7.6
	**PLT (10^3 cells/μL)**	995.6±167.9	858.2±201.0	1208±111	1164±78	1097±56	1217±63
	**WBC (10^3 cells/μL)**	3.48±0.43	4.24±0.97	5.62±1.00	5.88±1.13	4.71±0.35	4.38±0.37
**Clinical biochemical findings**	**TP (g/dL)**	6.10±0.05	6.06±0.14	6.10±0.23	6.04±0.04	6.02±0.09	6.20±0.13
	**ALB (g/dL)**	4.14±0.06	4.02±0.12	3.94±0.19	3.90±0.06	3.90±0.03	4.04±0.09
	**A/G ratio**	2.10±0.05	1.98±0.08	1.84±0.11	1.84±0.06	1.86±0.04	1.88±0.04
	**T-BIL (mg/dL)**	0.07±0.00	0.03±0.00*	0.05±0.01	0.03±0.01*	0.04±0.01	0.04±0.01
	**ALP (U/L)**	354.8±16.1	483.2±45.1	448.2±37.2	433.0±49.5	442.4±44.8	474.6±48.6
	**AST (U/L)**	83.00±2.51	96.60±6.17	92.60±13.1	92.00±2.00	82.00±4.32	84.20±7.04
	**ALT (U/L)**	26.20±0.80	27.00±2.55	31.20±5.00	25.40±2.38	33.20±2.06	29.60±1.96
	**CREA (mg/dL)**	0.43±0.02	0.46±0.03	0.43±0.01	0.41±0.02	0.39±0.01	0.43±0.02
	**BUN (mg/dL)**	19.88±2.57	15.58±1.01	15.94±0.88	13.46±1.22	14.62±0.86	16.86±1.08
	**T-CHO (mg/dL)**	86.80±9.28	85.80±3.81	87.00±4.55	86.60±6.59	86.40±3.82	97.80±9.41
	**TG (mg/dL)**	76.20±19.14	47.60±7.47	48.60±3.66	47.60±16.46	52.40±6.54	63.80±7.65
	**GLU (mg/dL)**	114.0±4.4	124.0±6.5	104.0±2.7	103.6±5.2	113.2±4.6	120.4±6.6
	**CA (mg/dL)**	10.04±0.10	9.96±0.13	10.16±0.20	10.08±0.14	10.28±0.04	10.44±0.10
	**IP (mg/dL)**	7.36±0.25	7.30±0.23	7.48±0.23	7.50±0.22	7.76±0.17	8.00±0.13
	**GGT (IU/L)**	0.34±0.09	0.17±0.07	0.21±0.13	0.32±0.14	0.20±0.13	0.43±0.12
	**CK (U/L)**	606.4±15.2	683.2±67.8	533.6±118.0	491.4±28.3	431.6±45.0	508.6±114.3
	**Na (mmol/L)**	140.8±0.3	141.6±0.5	141.7±0.7	141.9±0.3	141.1±0.3	141.2±0.3
	**K (mmol/L)**	4.44±0.07	4.29±0.14	4.50±0.16	4.44±0.09	4.54±0.12	4.43±0.22
	**Cl (mmol/L)**	100.2±0.3	102.5±0.4*	103.0±0.6**	101.6±0.9	102.3±0.4	101.5±0.3
**ELISA analysis**	**IgE (ng/mL)**	0.222±0.023	0.211±0.008	0.247±0.035	0.211±0.025	0.187±0.022	0.183±0.016
	**Histamine (ng/mL)**	0.253±0.030	0.311±0.043	0.385±0.027	0.257±0.034	0.570±0.128*	0.510±0.063*

Data were presented as mean ± standard error of the mean.

All data were analyzed using Kruskal-Wallis test and the significance of intergroup difference between the control and administered groups was assessed using Dunn's Rank Sum test. All statistical analyses were performed using GraphPad Prism 8.0 (GraphPad Software, Inc., San Diego, CA).

[*]vs. Control group: $p < 0.05$, [**] vs. Control group: $p < 0.01$

Findings of necropsy and histopathology

During the necropsy on all SD rats, no polysorbate 20 or HSA related macroscopic findings were observed such as changes in external appearance, internal organs, and administration sites. In histopathological examination of the administration site, mild macrophage infiltration and neovascularization were observed with a similar degree and frequency in all administered groups, including the control group. In addition, inflammatory cells infiltration, fat infiltration, giant cells, and necrosis were observed only in some individuals regardless of the administered substance and dose (Fig 2).

In the case of the NZW rabbits, macroscopic findings at necropsy and histopathological findings at the sites of administration were not observed regardless of the administered substance (Fig 3).

Fig 2. Histopathological section of the administration site in the four-week repeated intramuscular dose toxicity study in Sprague-Dawley (SD) rats. Hematoxylin and eosin stained histopathological section (original magnification 100x) of the gastrocnemius muscle of the SD rats: a) Control group, male; b) Control group, female; c) Polysorbate 20-0.02 mg/kg, male; d) Polysorbate 20-0.02 mg/kg, female; e) Polysorbate 20-0.1 mg/kg, male; f) Polysorbate 20-0.1 mg/kg, female; g) Polysorbate 20-0.4 mg/kg, male; h) Polysorbate 20-0.4 mg/kg, female; i) Human serum albumin-0.06 mg/kg, male; j) Human serum albumin-0.06 mg/kg, female; k) Human serum albumin-0.12 mg/kg, male; l) Human serum albumin-0.12 mg/kg, female. Mild macrophage infiltration, neovascularization, inflammatory cells infiltration, and fat infiltration were observed with a similar degree and frequency in all administered groups. An arrow pointing up (↑) indicated macrophage infiltration and an arrow pointing down (↓) indicated

neovascularization.

Fig 3. Histopathological section of the administration site in the intradermal irritation study in New Zealand White (NZW) rabbits. Hematoxylin and eosin stained histopathological section (original magnification 50x) of the dorsal back skin of the NZW rabbits: a) Control group; b) Polysorbate 20-0.02 mg/kg; c) Polysorbate 20-0.1 mg/kg; d) Polysorbate 20-0.4 mg/kg; e) Human serum albumin (HSA)-0.06 mg/kg; f) HSA-0.12 mg/kg, male. No histopathological findings were found at the site of administration in all administered groups.

Discussion

Polysorbate 20 was approved by the regulatory authorities as an additive in foods, cosmetics, eye drops, and tablets in various countries [14,15]. Therefore, most clinical and nonclinical safety studies of polysorbate 20 have been conducted using non-invasive routes of administration (e.g., oral, skin, and ophthalmic administration) [16,17]. In particular, as a food additive, polysorbate 20 has various *in vivo* safety data, including *in vitro* genotoxicity and DNA reactivity based on structural alerts [26]. Recently, polysorbate 20, which was one of the excipients of CORETOX® approved by the Ministry of Food and Drug Safety (MFDS, Republic of Korea), has been used in humans via an intramuscular route of administration [12]. However, there are few studies on the safety of polysorbate 20 when administered intramuscularly. In a study conducted in four monkeys, it was reported that intramuscular injection of 275 mg/kg of polysorbate20 for 20 days did not cause toxicity in the liver and kidney [27]. This study did not evaluate clinical signs, body weight, or hematology and clinical biochemistry, which are essential criteria for evaluating toxicity [28]. Therefore, the current study was conducted to verify the

safety of intramuscular administration of polysorbate 20 through four-week repeated intramuscular dose toxicity in SD rats and an intradermal irritation study in NZW rabbits. This study is the first to fully evaluate the *in vivo* safety of polysorbate 20 when administered intramuscularly based on current guidelines of non-clinical safety research.

To evaluate the systemic toxicity of intramuscular injection of polysorbate 20, a four-week repeated dose toxicity study in SD rats was conducted. According to the MFDS, the clinical dosage of CORETOX®, which contains 1 mg of polysorbate 20 in one vial, ranged from 0.2 vial (treatment of forehead wrinkles) to 3.6 vials (stiff muscles in the upper limbs after a stroke) [12]. Thus, the dose (0.02, 0.1, and 0.4 mg/kg) of polysorbate 20 in the current study was established based on the therapeutic indication of CORETOX® in humans and the animal equivalent dose calculation factor [19]. In addition, 0.9% saline was set as the vehicle control substance, while 0.06 and 0.12 mg/kg doses of HSA, which has an *in vivo* safety profile accumulated over the years for intramuscular administration, was set as a comparative substance [2,6].

Generally, mortality, body weight, and clinical signs have the most significance in repeated dose toxicity studies [29,30]. Furthermore, changes in the levels of hematology and clinical biochemistry in laboratory animals could enable more accurate predictions of human toxicity [31,32]. In the current study, adverse effects, including mortality, clinical signs, and macroscopic findings on necropsy, were not found at any dose level of HSA and polysorbate 20 administered during the study period in all SD rats. In addition, significant changes in the body weight and the level of hematology were not observed in the polysorbate 20 and HSA administered groups compared to the control group. In the clinical biochemistry test and allergy reaction test, significant decrease in albumin/globulin ratio ($p < 0.05$), glucose ($p < 0.05$), and total bilirubin ($p < 0.05$) and significant increase in sodium ($p < 0.05$ or $p < 0.01$), chloride ($p < 0.05$ or $p < 0.01$), and serum histamine ($p < 0.05$) were observed in the polysorbate 20 or HSA

administered group compared to the control group. However, these changes had no toxicological significance as they were not dose-responsive and the levels were within the normal range for the same age of SD rats [33,34]. This indicated that intramuscular administration of 0.02, 0.1, and 0.4 mg/kg dose of polysorbate 20 did not cause systemic toxicity in SD rats.

Histopathological examination of the administration site was conducted to observe the local toxicity of the polysorbate 20 in all SD rats. At the administration site, macrophage infiltration, neovascularization, giant cell, fibrosis, and necrosis were observed to a similar degree and frequency in all administered groups regardless of the administered substance. However, these findings were observed in most of the administered groups without a dose response, including the control group. Furthermore, inflammatory lesions could occur in the body of SD rats as a defense mechanism against intramuscular injection of any foreign substances [35]. Therefore, those findings were presumed to be due to the intramuscular injection rather than any effect of polysorbate 20. In other words, there were no toxicological changes at the site of administration after intramuscular administration of polysorbate 20 based on histopathology. Furthermore, serum IgE and histamine levels were measured to determine whether allergic reactions occurred after the administration of polysorbate 20 in all SD rats. In addition to clinical signs, increased levels of serum IgE and histamine are representative indicators of an allergic reaction [36–38]. In the current study, allergic reactions were not observed in response to the administration of polysorbate 20, which indicated that polysorbate 20 is not an allergen in SD rats.

However, despite its very low frequency, side effects in the allergy category have been reported in humans using shampoos (8.4% polysorbate 20, 2 cases among 5.88 million uses), cuticle softeners (2.0% polysorbate 20, 24 cases among 131 million use), and paste masks (2.0% polysorbate 20, 11 cases among 120.7 million uses) containing polysorbate 20 [14,15]. Therefore, an additional intradermal irritation study was conducted in NZW rabbits to confirm that

polysorbate 20 was not an allergen *in vivo*. Traditionally, the intradermal test is the gold standard method for diagnosing allergic reactions [39,40]. If an immune response, such as erythema, crust formation, and edema formation, occurs at the administration site after intradermal administration of a substance, it could be concluded that the substance was an allergen to the administered subjects [24]. In the current study, immune responses of the administration site were not observed at all polysorbate 20 treated NZW rabbits after intradermal administration during the study period, which is the same as the results in the control group and the HSA administered group. Furthermore, in the polysorbate 20 administered group, toxicological changes based on the histopathological examination of the site of administration and increased levels of serum IgE and histamine were not observed. There were also no toxicologically significant changes, such as changes in the body weight or the hematology and clinical biochemistry in all rabbits administered polysorbate 20 and all toxicological values in NZW rabbits were within the normal range for the same age of the NZW rabbits [41,42]. These results of the intradermal irritation study indicated that polysorbate 20 was not an allergen in NZW rabbits, which was consistent with the results of the four-week repeated dose toxicity study in SD rats.

Conclusions

The current study, a four-week repeated intramuscular dose toxicity study in SD rats and an intradermal irritation study in NZW rabbits, provides the basis for the *in vivo* safety profile of intramuscular administration of polysorbate 20, an excipient of newly developed BoNT/A products (CORETOX®). Polysorbate 20, which did not induce systemic and local toxicities in this study, was considered to be a safe substance for intramuscular administration, just like HSA for which a safety profile has been obtained over the years. In addition, since polysorbate 20 is a

synthetic product rather than an animal-derived product, it has the advantages of a consistent quality of the product, ease of manufacture and supply, and a low risk of pathogen transmission compared to HSA. Therefore, the findings of this study provide the scientific reliability of the use of polysorbate 20 as an alternative to HSA, which is used as an excipient for most BoNT/A products, particularly in terms of its safety. Furthermore, this study suggest the possibility that polysorbate 20, which is mainly used as an additive in foods, cosmetics, eye drops, and tablets, could be used as a safe excipient for various pharmaceuticals administered intramuscularly.

Acknowledgments

Not applicable

References

1. Fanali G, Di Masi A, Trezza V, Marino M, Fasano M, Ascenzi P. Human serum albumin: from bench to bedside. Mol Aspects Med. 2012;33: 209–290.

2. Zhou T, Lu S, Liu X, Zhang Y, Xu F. Review of the rational use and adverse reactions to human serum albumin in the People's Republic of China. Patient Prefer Adherence. 2013;7: 1207.

3. Bosse D, Praus M, Kiessling P, Nyman L, Andresen C, Waters J, et al. Phase I comparability of recombinant human albumin and human serum albumin. J Clin Pharmacol. 2005;45: 57–67.

4. Benedetto A V. What's new in cosmetic dermatology. Dermatol Clin. 2019;37: 117–128.

5. Park J-Y, Sunga O, Wanitphakdeedecha R, Frevert J. Neurotoxin Impurities: A Review of Threats to Efficacy. Plast Reconstr Surg Glob Open. 2020;8.

6. Malhotra R, Huilgol SC, Selva D. Botulinum toxin and human serum albumin. Arch Ophthalmol. 2003;121: 1661.

7. Chen Z, He Y, Shi B, Yang D. Human serum albumin from recombinant DNA technology: challenges and strategies. Biochim Biophys Acta (BBA)-General Subj. 2013;1830: 5515–5525.

8. Matejtschuk P, Dash CH, Gascoigne EW. Production of human albumin solution: a continually developing colloid. Br J Anaesth. 2000;85: 887–895.

9. Chamberland ME, Alter HJ, Busch MP, Nemo G, Ricketts M. Emerging infectious disease issues in blood safety. Emerg Infect Dis. 2001;7: 552.

10. Tumber PS, Louis I. Botulinum toxin type a therapy and human serum albumin. Anesthesiol J Am Soc Anesthesiol. 2006;104: 1108.

11. Norsworthy PJ, Thompson AGB, Mok TH, Guntoro F, Dabin LC, Nihat A, et al. A blood miRNA signature associates with sporadic Creutzfeldt-Jakob disease diagnosis. Nat Commun. 2020;11. doi:10.1038/s41467-020-17655-x

12. Lee J, Chun MH, Ko YJ, Lee S-U, Kim DY, Paik N-J, et al. Efficacy and safety of MT10107 (Coretox®) in post-stroke upper limb spasticity treatment: A randomized, double-blind, active drug-controlled, multi-center, phase III clinical trial. Arch Phys Med Rehabil. 2020.

13. Kwak S, Kang W, Rhee C-H, Yang G-H, Cruz DJM. Comparative Pharmacodynamics Study of 3 Different Botulinum Toxin Type A Preparations in Mice. Dermatologic Surg. 2020;46: e132–e138.

14. Moore J. Final report on the safety assessment of polysorbates 20, 21, 40, 60, 61, 65, 80, 81, and 85. J Am Coll Toxicol. 1984;3: 1–82.

15. Lanigan RS. Final report on the safety assessment of PEG-20 sorbitan cocoate; PEG-40 sorbitan diisostearate; PEG-2,-5, and-20 sorbitan isostearate; PEG-40 and-75 sorbitan lanolate; PEG-10,-40,-44,-75, and-80 sorbitan laurate; PEG-3, and-6 sorbitan oleate; PEG-80 sorb. Int J Toxicol. 2000;19: 43–89.

16. Rowe RC, Sheskey P, Quinn M. Handbook of pharmaceutical excipients. Libros Digitales-Pharmaceutical Press; 2009.

17. Lewis RJ, Sax N. Sax's dangerous properties of industrial materials. New York; 1996.

18. Percie du Sert N, Ahluwalia A, Alam S, Avey MT, Baker M, Browne WJ, et al. Reporting animal research: Explanation and elaboration for the ARRIVE guidelines 2.0. PLoS Biol. 2020;18: e3000411.

19. Nair AB, Jacob S. A simple practice guide for dose conversion between animals and human. J basic Clin Pharm. 2016;7: 27.

20. Diehl K, Hull R, Morton D, Pfister R, Rabemampianina Y, Smith D, et al. A good practice guide to the administration of substances and removal of blood, including routes and volumes. J Appl Toxicol An Int J. 2001;21: 15–23.

21. Oh H-M, Park JH, Song DH, Chung ME. Efficacy and safety of a new botulinum toxin type A free of complexing proteins. Toxins (Basel). 2016;8: 4.

22. Aoki KR. A comparison of the safety margins of botulinum neurotoxin serotypes A, B, and F in mice. Toxicon. 2001;39: 1815–1820.

23. Kim S-H, Kim S-B, Yang G-H, Rhee C-H. Mouse compound muscle action potential assay: An alternative method to conduct the LD50 botulinum toxin type A potency test. Toxicon. 2012;60: 341–347.

24. Iso B, STANDARD B. Biological evaluation of medical devices. Part. 2009;1: 10993.

25. Leary SL, Underwood W, Anthony R, Cartner S, Corey D, Grandin T, et al. AVMA guidelines for the euthanasia of animals: 2013 edition. American Veterinary Medical Association Schaumburg, IL; 2013.

26. (ANS) EP on FA and NS added to F. Scientific Opinion on the re- evaluation of polyoxyethylene sorbitan monolaurate (E 432), polyoxyethylene sorbitan monooleate (E 433), polyoxyethylene sorbitan monopalmitate (E 434), polyoxyethylene sorbitan monostearate (E 435) and polyoxyethylene sorbita. EFSA J. 2015;13: 4152.

27. Krantz JC, Carr CJ, Bird JG, Cook S. SUGAR ALCOHOLS: XXVI. Pharmacodynamic studies of polyoxyalkylene derivatives of hexitol anhydride partial fatty acid esters. J Pharmacol Exp Ther. 1948;93: 188–195.

28. Guideline ICHHT. Guidance on nonclinical safety studies for the conduct of human clinical trials and marketing authorization for pharmaceuticals M3 (R2). International conference on harmonisation of technical requirements for registration of pharmaceuticals for human use. 2009.

29. Liu T, Li L, Teng X, Huang X, Liu H, Chen D, et al. Single and repeated dose toxicity of mesoporous hollow silica nanoparticles in intravenously exposed mice. Biomaterials. 2011;32: 1657–1668.

30. Shende P, Kulkarni YA, Gaud RS, Deshmukh K, Cavalli R, Trotta F, et al. Acute and repeated dose toxicity studies of different β- cyclodextrin- based nanosponge formulations. J Pharm Sci. 2015;104: 1856–1863.

31. El Hilaly J, Israili ZH, Lyoussi B. Acute and chronic toxicological studies of Ajuga iva in experimental animals. J Ethnopharmacol. 2004;91: 43–50.

32. Rhiouani H, El-Hilaly J, Israili ZH, Lyoussi B. Acute and sub-chronic toxicity of an aqueous extract of the leaves of Herniaria glabra in rodents. J Ethnopharmacol. 2008;118: 378–386.

33. Lillie LE, Temple NJ, Florence LZ. Reference values for young normal Sprague-Dawley

rats: weight gain, hematology and clinical chemistry. Hum Exp Toxicol. 1996;15: 612–616.

34. Petterino C, Argentino-Storino A. Clinical chemistry and haematology historical data in control Sprague-Dawley rats from pre-clinical toxicity studies. Exp Toxicol Pathol. 2006;57: 213–219.

35. Suttie AW. Boorman's Pathology of the Rat. Elsevier; 2015.

36. Wachholz PA, Dearman RJ, Kimber I. Detection of allergen-specific IgE antibody responses. J Immunotoxicol. 2005;1: 189–199.

37. Barbee RA, Brown WG, Kaltenborn W, Halonen M. Allergen skin-test reactivity in a community population sample: correlation with age, histamine skin reactions, and total serum immunoglobulin E. J Allergy Clin Immunol. 1981;68: 15–19.

38. Tanizaki Y, Komagoe H, Morinaga H, Kitani H, Goda Y, Kimura I. Allergen-and anti-IgE-induced histamine release from whole blood. Int Arch Allergy Immunol. 1984;73: 141–145.

39. Ruzicka T, Gerstmeier M, Przybilla B, Ring J. Allergy to local anesthetics: comparison of patch test with prick and intradermal test results. J Am Acad Dermatol. 1987;16: 1202–1208.

40. Wood RA, Phipatanakul W, Hamilton RG, Eggleston PA. A comparison of skin prick tests, intradermal skin tests, and RASTs in the diagnosis of cat allergy. J Allergy Clin Immunol. 1999;103: 773–779.

41. Gallego M. Laboratory reference intervals for systolic blood pressure, rectal temperature, haematology, biochemistry and venous blood gas and electrolytes in healthy pet rabbits.

Open Vet J. 2017;7: 203–207.

42. Hewitt CD, Innes DJ, Savory J, Wills MR. Normal biochemical and hematological values in New Zealand white rabbits. Clin Chem. 1989;35: 1777–1779.

Supporting information

S1 Table. Individual body weights of male (n: 36) and female (n: 30) Sprague-Dawley (SD) rats in the current study.

S2 Table. Individual body weights of male New Zealand White (NZW) rabbits (n: 18) in the current study.

S3 Table. Individual levels of hematology, clinical biochemistry, and serum IgE and histamine in Sprague-Dawley (SD) rats in the current study.

S4 Table. Individual level of hematology, clinical biochemistry, and serum IgE and histamine in New Zealand White (NZW) rabbits in the current study.

Figure 1

Click here to access/download;Figure;Fig 1.tif

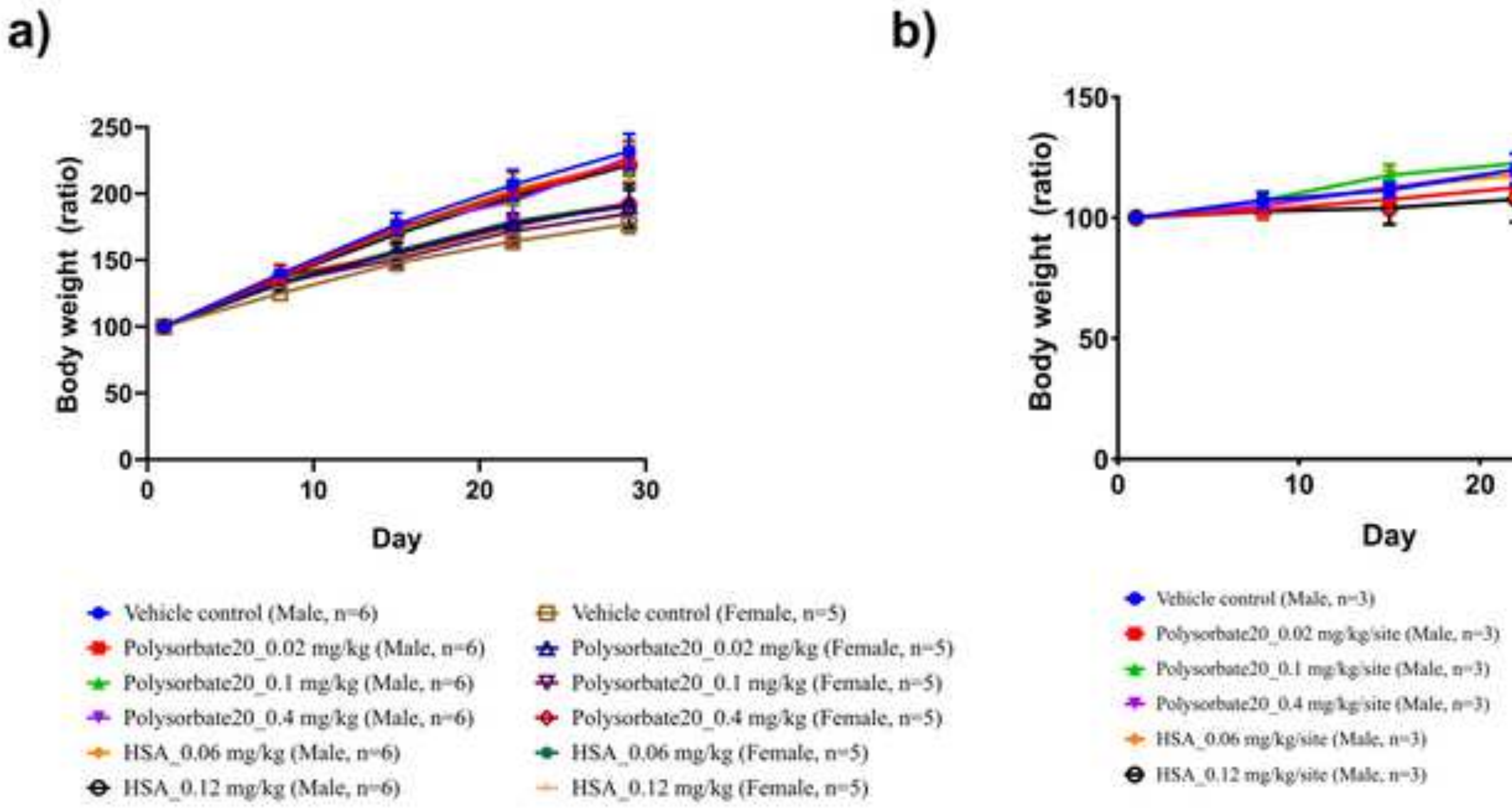

Click here to access/download;Figure;Fig 3.tif

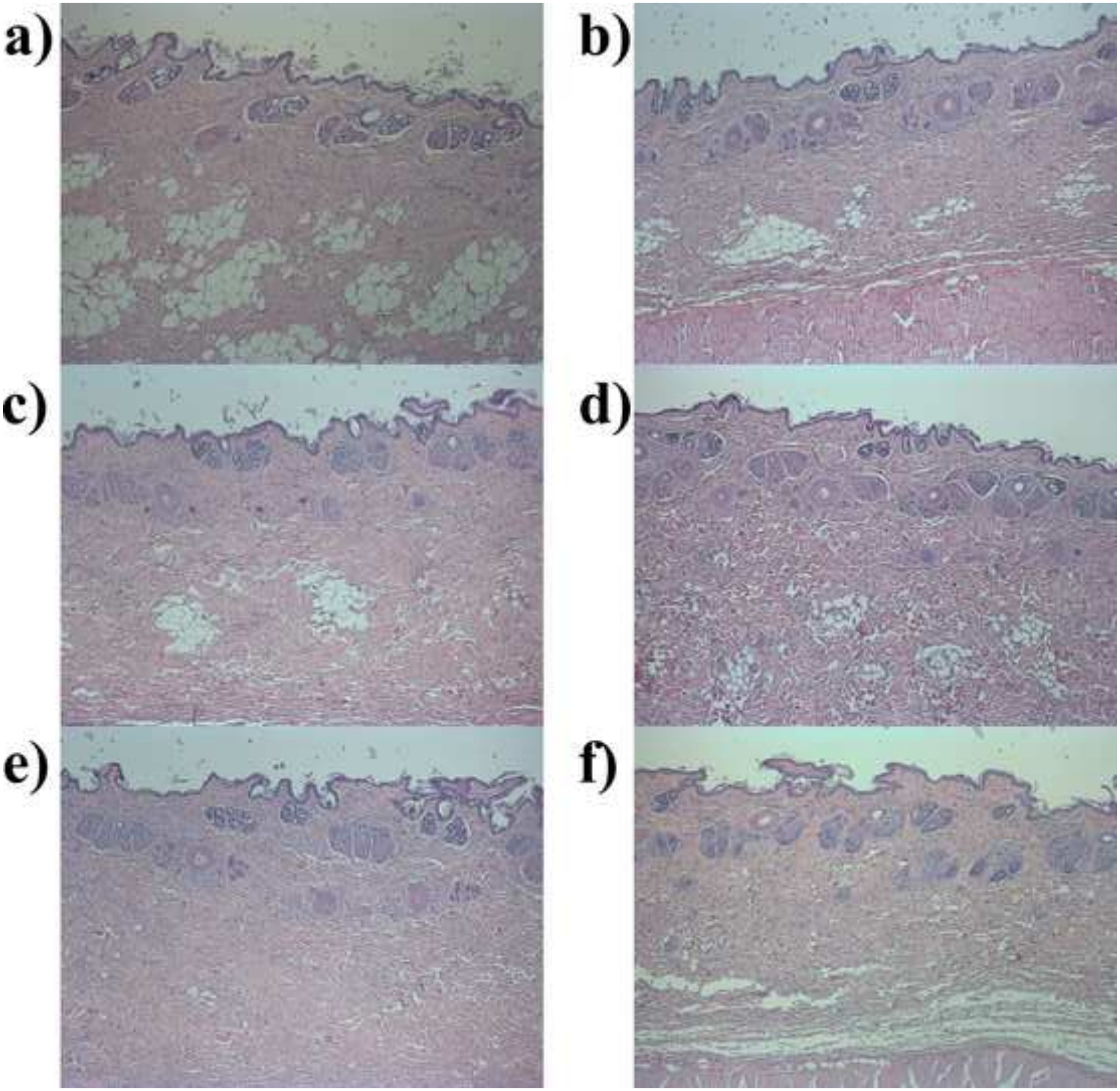

Click here to access/download;Figure;Revised Figure 2.tif

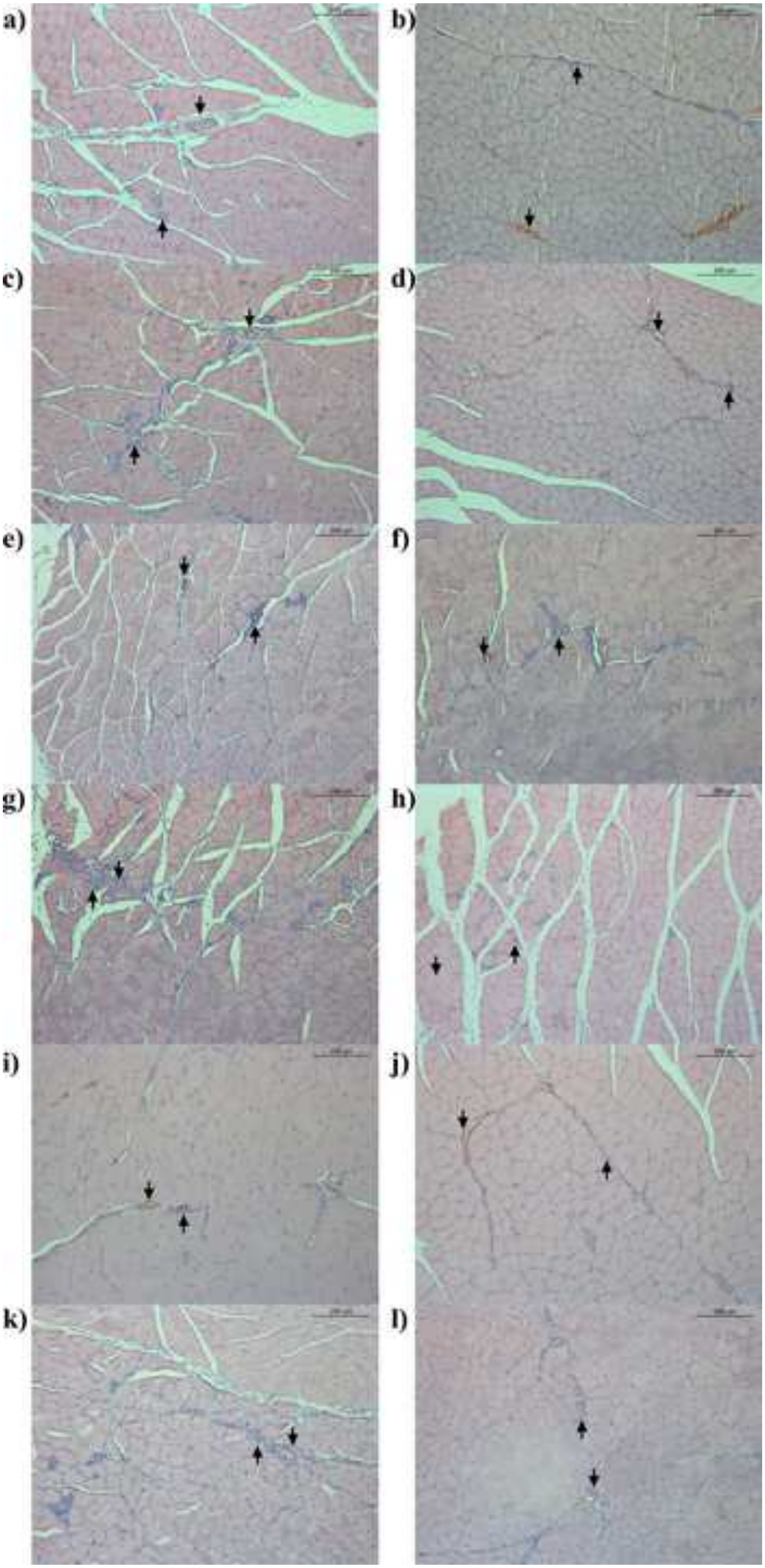

Supporting Information 1

Click here to access/download
Supporting Information
S1 Table.xlsx

Click here to access/download
Supporting Information
S2 Table.xlsx

Click here to access/download
Supporting Information
S3 Table.xlsx

Supporting Information 4

Click here to access/download
Supporting Information
S4 Table.xlsx

Safety verification for polysorbate 20, pharmaceutical excipient for intramuscular administration, in Sprague-Dawley rats and New Zealand White rabbits

Short title: *In vivo* safety evaluation of Polysorbate 20

Junhyung Kim[1¶], Seongsung Kwak[1,4¶], Mi-Sun Park[1], Chang-Hoon Rhee[2], Gi-Hyeok Yang[1], Jangmi Lee[3], Woo-Chan Son[4&*], and Won-ho Kang[1&*]

[1]Gwanggyo R&D Center, Medytox Inc., Suwon-si, Gyeonggi-do, Korea

[2]Osong R&D Center, Medytox Inc., Cheongju-si, Chungcheongbuk-do, Korea

[3]Department of Medical Science, Asan Medical Institute of Convergence Science and Technology, Asan Medical Center, University of Ulsan College of Medicine, Songpa-gu, Seoul, Korea

[4]Department of Pathology, Asan Medical Center, Songpa-gu, Seoul, Korea

*Corresponding author

E-mail: whkang@medytox.com (WhK)

E-mail: wcson32@hanmail.net (WCS)

[¶]These authors contributed equally to this work as first author.

[&]These authors contributed equally to this work as corresponding author.

Abstract

Human serum albumin (HSA) has been widely used as a pharmaceutical excipient in Botulinum toxin serotype A (BoNT/A) products that are indicated for use in therapeutics and cosmetics. However, HSA as a human-derived material has some concerns, such as the potential risk of transmission of infectious agents, an insufficient supply, and difficulty in maintaining a certain quality. For those reasons, newly developed BoNT/A products (CORETOX®, Medytox, Inc., Republic of Korea) contained polysorbate 20, a non-human-derived excipient, to replace the HSA. However, most safety studies of polysorbate 20 have been conducted with non-invasive routes of administration, and thus there are a few studies on the safety of polysorbate 20 when administered intramuscularly. To secure the *in vivo* safety profile of polysorbate 20, a four-week repeated intramuscular dose toxicity study (0.02, 0.1, and 0.4 mg/kg, one injection every two weeks for a total of three injections) was conducted in 66 Sprague-Dawley (SD) rats. Then, an intradermal irritation study was further conducted with 18 New Zealand White (NZW) rabbits. The toxicological evaluation of HSA (0.06 and 0.12 mg/kg) was also carried out as a comparative substance. Systemic and local toxicities were not observed in any of the SD rats or NZW rabbits based on clinical signs, body weight, hematology, clinical biochemistry, macroscopic findings on necropsy, histopathology of the injection site, and allergic reactions. The current study suggested that intramuscular administration of polysorbate 20 was considered to be safe at a level similar to that of HSA, which has an *in vivo* safety profile accumulated over the years. This provided the basis for the *in vivo* safety profile of polysorbate 20 administered intramuscularly and the scientific reliability of the use of polysorbate 20 as an alternative to HSA, which is used as an excipient for various pharmaceuticals in terms of its safety.

Introduction

Human serum albumin (HSA), the most abundant protein in human plasma, has the functions of regulating the colloid osmotic pressure, binding to and transporting various molecules, antioxidant action, anticoagulant action, regulating membrane permeability, and a direct neuroprotective action [1,2]. Therefore, HSA has been used for therapeutic purposes in patients with hemorrhage, hypovolemia, and hypoalbuminemia since about the 19th century [2]. In addition to therapeutic purposes, it is used as an excipient for various pharmaceuticals in biomedical aspects, [1,3].

Most botulinum toxin serotype A (BoNT/A) products that are indicated for use in therapeutics and cosmetics also use HSA as an excipient [4,5]. In BoNT/A products, HSA plays a role in stabilizing the neurotoxic proteins during manufacturing, transportation, and administration and in preventing the aggregation of neurotoxic proteins [6]. However, there are several concerns with HSA, although it is produced under strict regulations and has a safety profiling accumulated over the years [2,6]. Since the production of HSA mainly relies on human blood donation, the quality of HSA could differ depending on the population of the human blood donors (race or geographical distribution) [7]. Furthermore, the market demand for HSA has dramatically increased (over 500 tons), and it is one of the most widely used biopharmaceutical solutions today [7,8]. This could lead to a worldwide shortage of HSA in the market [2]. Finally, the use of HSA has a theoretical risk of spreading blood-derived pathogens [6,9]. Currently, the transmission of viral diseases, such as hepatitis B virus, human immunodeficiency virus, and West Nile virus are well controlled through strict management of HSA, but there are still concerns about the transmission of prion-derived variant Creutzfeldt-Jakob disease [10,11].

For those reasons, Medytox, Inc. (Republic of Korea) has recently developed a

complexing protein-free BoNT/A product (CORETOX®) that uses polysorbate 20, a non-human-derived excipient, to replace the HSA [12,13]. Polysorbates, a series of polyoxyethylenated sorbitan esters, are hydrophilic and nonionic surfactants that have been widely used as a food additive and stabilizer in pharmaceuticals [14,15]. Among them, polysorbate 20, called polyoxyethylene-20-sorbitan monolaurate, has 20 repeating units of polyethylene glycol. It is mainly used as a direct/indirect additive in food and emulsifiers/surfactants in cosmetics that come into direct contact with the human skin, such as shampoo, bath soap, skin cleansing products, and hair spray [15]. It has also been used as an excipient in pharmaceuticals as a stabilizer, particularly in tablet and ophthalmic solutions. Hence, most of the safety studies on polysorbate 20 have been conducted on oral toxicity and skin irritation [14]. As a result, it was found that excessive dose of polysorbate20 could induce mild/temporary eye irritation and skin irritation [14–17]. In addition, when administered orally, it adversely affects the gastro-intestinal tract and, in severe cases, could cause death [14,16]. However, there are a few studies on the safety of polysorbate 20 administered by other routes, especially intramuscular administration, which is the route of BoNT/A injection.

The objective of this study was to verify the *in vivo* safety properties of polysorbate 20 when administered intramuscularly. First, a four-week repeated intramuscular dose toxicity study in Sprague-Dawley (SD) rats (one injection every two weeks for a total of three injections) was carried out to evaluate its systemic toxicity. Second, an intradermal irritation study in New Zealand White (NZW) rabbits was carried out to evaluate local toxicity and allergic reactions. It was expected the current study could enable the evaluation of the safety of polysorbate 20 for intramuscular administration and would provide the basis for the *in vivo* safety profile of polysorbate 20 as a pharmaceutical excipient.

Materials and methods

Animals and facility

This study was reviewed and approved by the Institutional Animal Care and Use Committee of Medytox, Inc. (A-2019-009) and the Korea Testing & Research Institute (KTR, IAC2019-1500). In addition, the study design was established based on the ARRIVE guidelines [18].

In the four-week repeated intramuscular dose toxicity study performed at Medytox R&D Center (Republic of Korea), a total of 66 SD rats, consisting of 30 females (130.7 ~ 146.2g) and 36 males (163.5 ~ 187.2g), were used (Table 1). Healthy five-week-old SD rats were purchased from Orient Bio, Inc. (Republic of Korea). Upon receipt, all animals were macroscopically examined and acclimated for 7 days to the laboratory conditions (a specific pathogen-free animal room; temperature, 23 ± 3°C; humidity, 55 ± 15%; ventilation, 15 air changes per hour; light/dark cycle, 12 hours; light intensity, 150 ~ 300 Lux; polycarbonate cage; 2 ~ 3 animals per cage). Then, animals were divided into six groups with 11 animals (six male and five female SD rats) in each group. Group 1 (control group) were administered 0.9% saline every two weeks. Group 2, 3, and 4 were administered 0.02, 0.1, and 0.4 mg/kg of polysorbate 20 every two weeks, respectively. Group 5 and 6 were administered 0.06 and 0.12 mg/kg of HSA every two weeks, respectively.

In the intradermal irritation study performed at KTR (Republic of Korea), a total of 18 male NZW rabbits (2307.7 ~ 2947.8 g) were used (Table 1). Healthy three-month-old rabbits were purchased from DooYeol Biotech (Republic of Korea). Upon receipt, all animals were macroscopically examined and acclimated for 6 days to the laboratory conditions (specific pathogen-free animal room; temperature, 20.3 ~ 21.9°C; humidity, 54.5 ~ 64.9%; ventilation, 10~ 20 air changes per hour; light/dark cycle, 12 hours; light intensity, 150 ~ 300 Lux; stainless steel

cage; 1 animal per cage). Then, animals were divided into six groups with 3 male NZW rabbits in each group. Group 1 (control group) were administered 0.9% saline once. Group 2, 3, and 4 were administered 0.02, 0.1, and 0.4 mg/kg/site of polysorbate 20 once, respectively. Group 5 and 6 were administered 0.06 and 0.12 mg/kg/site of HSA once, respectively.

Table 1. Study design of four-week repeated intramuscular dose toxicity study in Sprague-Dawley rats and intradermal irritation study in New Zealand white rabbits

**Study**	**Species**	**Group**	**Substance**	**Dose volume**	**Dose**	**Route and Frequency of administration**	**No. animals (Male/Female)**
**Four-week repeated intramuscular dose toxicity study**	**Sprague-Dawley Rats**	G1	Control (0.9 % saline)	0.2 mL/kg	0 mg/kg	Intramuscular injection (Gastrocnemius muscle) Once per 2 weeks (Total 3 times)	11 (6/5)
		G2	Polysorbate 20		0.02 mg/kg		11 (6/5)
		G3	Polysorbate 20		0.1 mg/kg		11 (6/5)
		G4	Polysorbate 20		0.4 mg/kg		11 (6/5)
		G5	Human serum albumin		0.06 mg/kg		11 (6/5)
		G6	Human serum albumin		0.12 mg/kg		11 (6/5)
		**Total**					**66 (36/30)**
**Intradermal irritation study**	**New Zealand White rabbits**	G1	Control (0.9 % saline)	0.4 mL/site (Total 5 sites)	0 mg/kg/site	Intradermal injection (Dorsal back skin) Once (Total 1 time)	3 (3/0)
		G2	Polysorbate 20		0.02 mg/kg/site		3 (3/0)
		G3	Polysorbate 20		0.1 mg/kg/site		3 (3/0)
		G4	Polysorbate 20		0.4 mg/kg/site		3 (3/0)
		G5	Human serum albumin		0.06 mg/kg/site		3 (3/0)
		G6	Human serum albumin		0.12 mg/kg/site		3 (3/0)
		**Total**					**18 (18/0)**

Vehicle, positive control substance, and test substance

Saline (0.9% NaCl, DAI HAN PHARM. Co., Republic of Korea) was used as the vehicle in this study conducted in SD rats and NZW rabbits. HSA (Green Cross Corp., Republic of Korea) was selected as the comparative substance for the polysorbate 20 (Merck, Germany) that was the test substance of the current study. In the four-week repeated intramuscular dose toxicity study, the dose of HSA was calculated by multiplying the animal equivalent dose calculation factor (6.2) and the content of HSA in most BoNT/A products, 0.01 and 0.02 mg/kg (0.5 and 1.0 mg/vial, 50 kg human) [4,19]. As a result, 0.06 and 0.12 mg/kg were selected as the dose of HSA. The dose of polysorbate 20 was selected based on the content of polysorbate 20 in CORETOX® (1.0 mg/vial, 60 kg human) and the human dosage of CORETOX® (0.2 ~ 3.6 vial) [12,13]. Then, the dose of polysorbate 20 was also calculated in the same manner as when determining the dose of HSA. As a result, 0.02, 0.1, and 0.4 mg/kg were selected as the dose of polysorbate 20.

The same dose of HSA and polysorbate 20 used in the four-week repeated intramuscular dose toxicity study was applied in the intradermal irritation study in NZW rabbits. Detailed information is provided in Table 1.

In vivo administration

In the four-week repeated intramuscular dose toxicity study in SD rats, all substances (saline, HSA, polysorbate 20) were administered intramuscularly. The dosing formulation was prepared on the day of administration using saline (HSA: 0.3, 0.6 mg/mL; polysorbate 20: 0.1, 0.5, 2.0 mg/mL). The dose volume was selected as 0.2 mL/kg and the individual dose volume for each animal was calculated based on the body weight of the animal on the day of administration [20]. All substances were injected once every two weeks for four weeks (a total of 3 times; days 1, 15, 29) into one site of the gastrocnemius muscle of the right hindlimb. The dosing schedule

(interval and frequency) was set based on the clinical application schedule (total 1 time, at least three month dosing interval) and the administration site (gastrocnemius muscle) was selected based on the site generally used to evaluate the effectiveness of BoNT/A in rodents [12,21–23].

In the intradermal irritation study in NZW rabbits, all substances were administered via the intradermal route. The dosing formulation was prepared on the day of administration using saline (HSA: 0.15, 0.3 mg/mL; polysorbate 20: 0.05, 0.25, 1.0 mg/mL). The dose volume was selected as 0.4 mL/kg/site (total 2 mL/kg/head) and the individual dose volume for each animal was calculated based on the body weight of animal on the day of administration [20]. Before administration, the back skin of the rabbit was depilated of hair. Then, all substances were injected once into five sites (three left side and two right side) on the back skin (total 1 time; day 1) based on the guideline of the International Organization for Standardization (ISO) [24].

Clinical observation and body weights measurement

In the four-week repeated intramuscular dose toxicity study in SD rats, all animals were observed for clinical signs and general condition once daily throughout the study period (days 1 ~ 29). Their body weights were measured once a week (total five times), including the day of administration and at autopsy.

In the intradermal irritation study in NZW rabbits, clinical observation was conducted once daily throughout the study period (days 1 ~ 29). Furthermore, skin reactions at the administration site, including the degree of erythema, crust formation, and edema formation, were observed once daily based on the scoring system for skin reactions of the ISO [24]. Body weights were measured once a week (total five times), including on the day of administration and at autopsy.

Hematology, clinical biochemistry, and allergic reaction test

All animals (SD rats and NZW rabbits) were fasted overnight before the last day of the study. Then, the SD rats were anesthetized with isoflurane and blood samples were collected from their abdominal vein. In the NZW rabbits, blood samples were collected from the marginal ear vein without anesthesia.

Approximately 1 mL of the blood sample of all animals was put into a tube containing EDTA. After that, the following parameters were analyzed in KTR using a hematological autoanalyzer (ADVIA 120, SIEMENS, Germany): white blood cell count (WBC), differential leucocyte count (neutrophils; lymphocytes; monocytes; eosinophils; basophils), red blood cell count (RBC), hemoglobin (HGB), hematocrit (HCT), RBC indices (mean corpuscular volume, MCV; mean corpuscular hemoglobin, MCH; mean corpuscular hemoglobin concentration, MCHC), reticulocyte, and platelets.

The blood samples (except those used in the hematological analysis) were centrifuged at 3,000 rpm for 10 minutes to obtain the serum. Then, the following parameters were examined in KTR using an automatic analyzer (TBA-120FR, TOSHIBA, Japan): total protein, albumin, albumin/globulin ratio, total bilirubin, total bile acid, alkaline phosphatase (ALP), aspartate aminotransferase (AST), aspartate aminotransferase (AST), alanine aminotransferase (ALT), gamma glutamyltransferase (GGT), creatinine, blood urea nitrogen (BUN), total cholesterol, triglycerides (TG), glucose, calcium, inorganic phosphorus, creatine phosphokinase, cholinesterase, sodium, potassium, and chloride. Among these, total bile acid and cholinesterase were only analyzed for the NZW rabbits.

In addition, the levels of serum IgE and histamine of all animals were measured using an

enzyme-linked immunosorbent assay (ELISA) kit.

Necropsy and histopathology

After blood sampling from the SD rats and NZW rabbits, autopsies were performed on all animals at day 29 according to the AVMA Guidelines for the Euthanasia of Animals [25]. All SD rats were euthanized by exsanguination from the posterior vena cava and abdominal aorta under isoflurane anesthesia. All NZW rabbits were euthanized by intravenous injection of T-61 solution (MSD animal health, Republic of Korea). At necropsy, their external appearance, internal organs, and injection sites were observed macroscopically. Then, the injection site (one site of the gastrocnemius muscle of the right hindlimb for the SD rats and the five sites of the back skin for the NZW rabbits) were collected and stored in 10% neutral buffered formalin. Slides of all preserved tissues for histopathology were prepared through general tissue processing, including dehydration and paraffin embedding. All slides were then stained with hematoxylin and eosin and examined microscopically.

Statistical analysis

The Kruskal-Wallis test was performed to compare toxicological indicators, including body weights, hematology level, clinical biochemistry level, and serum IgE and histamine levels among the groups of SD rats and NZW rabbits. In the case of body weight, since there was a difference in the mean body weight among the groups before the first dose administration, the statistical analysis was performed using the ratio of body weight at the time of the measurement to the body weight before the first administration. The significance of intergroup difference

between the control and administered groups was assessed using Dunn's Rank Sum test. All statistical analyses were performed using GraphPad Prism 8.0 (GraphPad Software, Inc., San Diego, CA) and a *p*-value of < 0.05 was accepted to indicate statistical significance.

Results

Mortality and clinical Signs

In the four-week repeated intramuscular dose toxicity study, all SD rats remained well throughout the study and showed no clinical signs of disease, pain or distress. In the intradermal irritation study, no deaths or abnormal findings were observed in all NZW rabbits. Skin reactions, including erythema, eschar formation, and edema formation, were not observed regardless of the administered substance.

Changes in body weights

For both the SD rats and NZW rabbits, their body weight was measured once a week for a total of five times. Individual body weights of all animals by time point are presented in the S1 and S2 Tables.

In the four-week repeated intramuscular dose toxicity study, a gradual body weight gain of the SD rats was observed regardless of the administered substance (Fig 1a). Compared to the body weight before the first administration, all male SD rats had a body weight of 138.4% at day 8, 173.6% at day 15, 200.4% at day 22, and 225.0% at day 29, respectively, while all female SD rats had a body weight of 132.2% at day 8, 153.1% at day 15, 173.1% at day 22, and 187.3% at

day 29, respectively. There was no significant increase or decrease of the body weight of the SD rats administered polysorbate 20 and HSA compared to the control group at any of the time points.

In the intradermal irritation study, a gradual body weight gain of the NZW rabbits was also observed regardless of the administered substance (Fig 1b). Compared to their body weight before administration, all NZW rabbits had a weight of 105.0% at day 8, 111.0% at day 15, 116.7% at day 22, and 120.8% at day 29. There was also no significant difference in body weight of the NZW rabbits administered polysorbate 20 and HSA compared to the control group at any of the time points.

Fig 1. Body weight changes in a four-week repeated intramuscular dose toxicity study in Sprague-Dawley (SD) rats and an intradermal irritation study in New Zealand White (NZW) rabbits. Body weight changes measured once a week in a) SD rats and b) NZW rabbits, for a total of five times during the study period. In SD rats, all substances were injected once every two weeks for four weeks (total three times; days 1, 15, 29) into one site of the gastrocnemius muscle of the right hindlimb. Neither male nor female rat groups showed significant differences in body weight on days 8, 15, 22, and 29 (Kruskal-Wallis test). In NZW rabbits, all substances were injected once into five sites of the back skin (total one time; day 1). There was no significant difference in body weight on days 8, 15, 22, and 29 in all groups (Kruskal-Wallis test).

Findings of the level of hematology, clinical biochemistry, and serum IgE and histamine

Hematological tests and clinical biochemistry tests were performed on all SD rats and NZW rabbits. Individual results are presented in the S3 and S4 Tables. In both male and female SD rats, there were no polysorbate 20 or HSA related changes in the hematology parameters. In clinical biochemistry, compared to the control group, the albumin/globulin ratio was significantly decreased in male group 6 ($p < 0.05$), the level of glucose was significantly decreased in male group 3, 5, and 6 ($p < 0.05$, respectively), and the level of sodium was significantly increased in male group 4 ($p < 0.01$) and 6 ($p < 0.05$) (Table 2). In females, the level of total bilirubin was significantly decreased in group 2 and 4 ($p < 0.05$, respectively) and the level of chloride was significantly increased in group 2 ($p < 0.05$) and 3 ($p < 0.01$), compared to the control group (Table 3). Furthermore, in the allergic reaction test (ELISA analysis of serum IgE and histamine), significantly higher serum histamine levels were found in the female group 5 and 6 compared to the control group ($p < 0.05$).

In NZW rabbits, there were no changes in the hematology and clinical biochemistry parameters in the polysorbate 20 or HSA administered animals. There was also no significant increase or decrease in the level of serum IgE and histamine in NZW rabbits administered polysorbate 20 and HSA compared to the control group.

Table 2. The level of hematology, clinical biochemistry, and serum IgE and histamine in male Sprague-Dawley (SD) rats in the current study.

Category		Male					
		G1	G2	G3	G4	G5	G6
Hematological findings	RBC (10^6cells/μL)	7.37±0.13	7.38±0.14	7.31±0.11	7.48±0.14	7.50±0.10	7.37±0.14
	HGB (g/dL)	14.52±0.20	14.57±0.22	14.57±0.31	14.58±0.23	14.7±0.12	14.37±0.24
	HCT (%)	46.30±0.40	46.15±0.68	45.87±0.85	46.22±0.57	46.42±0.36	45.67±0.89
	MCV (fL)	62.95±0.81	62.57±0.55	62.73±0.56	61.82±0.57	61.95±1.00	62.02±0.09
	MCH (pg)	19.72±0.27	19.73±0.16	19.90±0.17	19.50±0.31	19.57±0.27	19.50±0.13
	MCHC (g/dL)	31.33±0.19	31.60±0.07	31.72±0.24	31.55±0.23	31.62±0.12	31.48±0.19
	RETIC (10^9cells/μL)	218.7±8.4	213.2±9.4	225.7±11.4	238.7±4.2	198.7±11.3	222.8±10.0
	PLT (10^3 cells/μL)	1156±63	1168±55	1161±66	1210±39	1228±28	1170±47
	WBC (10^3 cells/μL)	10.81±1.14	10.90±0.81	9.68±0.95	8.56±0.44	7.15±0.76	8.14±0.87
Clinical biochemical findings	TP (g/dL)	5.90±0.06	6.07±0.08	5.97±0.11	6.00±0.09	6.10±0.09	6.13±0.10
	ALB (g/dL)	3.85±0.04	3.88±0.03	3.83±0.06	3.85±0.03	3.97±0.04	3.88±0.06
	A/G ratio	1.88±0.04	1.78±0.03	1.78±0.04	1.80±0.04	1.87±0.04	1.73±0.02*
	T-BIL (mg/dL)	0.04±0.00	0.05±0.00	0.04±0.00	0.03±0.00	0.03±0.00	0.05±0.00
	ALP (U/L)	596.3±33.6	711.0±57.2	532.3±38.5	577.2±30.6	560.2±35.6	647.5±47.3
	AST (U/L)	90.33±6.35	119.2±30.2	92.67±4.34	81.00±4.46	106.5±4.8	101.5±9.7
	ALT (U/L)	28.33±3.46	45.50±16.37	27.17±2.85	22.83±1.56	29.00±2.66	26.17±2.44
	CREA (mg/dL)	0.44±0.01	0.45±0.02	0.46±0.02	0.44±0.02	0.40±0.01	0.42±0.01
	BUN (mg/dL)	11.78±0.60	12.60±1.00	11.93±0.54	10.42±0.74	11.17±0.48	10.90±0.41
	T-CHO (mg/dL)	71.00±3.39	78.50±4.65	76.33±4.26	72.33±5.33	65.83±5.68	82.00±3.01
	TG (mg/dL)	80.33±7.06	99.67±13.84	112.5±15.3	96.83±13.26	81.33±9.54	93.67±17.89
	GLU (mg/dL)	127.7±2.9	111.0±4.2	103.5±3.7*	117.0±7.9	99.33±3.33*	99.50±6.82*
	CA (mg/dL)	10.15±0.11	10.23±0.05	10.13±0.10	10.32±0.06	10.00±0.10	10.23±0.14
	IP (mg/dL)	8.90±0.18	8.68±0.17	8.63±0.15	8.78±0.13	9.02±0.09	8.90±0.11
	GGT (IU/L)	0.39±0.16	0.34±0.18	0.69±0.26	0.34±0.16	0.19±0.09	0.48±0.26
	CK (U/L)	529.7±45.1	529.7±29.3	578.5±60.3	452.5±39.0	745.5±50.4	652.3±79.4
	Na (mmol/L)	143.0±0.3	144.4±0.5	143.6±0.3	145.2±0.4**	144.3±0.4	144.9±0.4*
	K (mmol/L)	4.68±0.08	4.68±0.06	4.76±0.08	4.87±0.12	4.95±0.05	4.89±0.10
	Cl (mmol/L)	101.4±0.3	102.8±0.5	101.4±0.8	103.1±0.5	101.5±0.6	102.3±0.6
ELISA analysis	IgE (ng/mL)	0.233±0.014	0.270±0.001	0.246±0.015	0.181±0.008	0.200±0.016	0.188±0.004
	Histamine (ng/mL)	0.480±0.040	0.383±0.038	0.402±0.034	0.423±0.026	0.519±0.052	0.390±0.040

Data were presented as mean ± standard error of the mean.

All data were analyzed using Kruskal-Wallis test and the significance of intergroup difference between the control and administered groups was

assessed using Dunn's Rank Sum test. All statistical analyses were performed using GraphPad Prism 8.0 (GraphPad Software, Inc., San Diego, CA).

*vs. Control group: $p < 0.05$, ** vs. Control group: $p < 0.01$

Table 3. The level of hematology, clinical biochemistry, and serum IgE and histamine in female Sprague-Dawley (SD) rats in the current study.

Category		Female					
		G1	G2	G3	G4	G5	G6
**Hematological findings**	**RBC (10^6cells/μL)**	7.14±0.20	6.72±0.17	7.06±0.08	7.30±0.09	7.47±0.22	7.30±0.13
	**HGB (g/dL)**	14.08±0.30	13.78±0.31	13.96±0.20	14.58±0.12	14.74±0.28	14.52±0.28
	**HCT (%)**	42.54±0.88	42.08±1.01	42.06±0.46	44.42±0.16	44.60±0.88	44.40±0.76
	**MCV (fL)**	59.70±0.89	62.70±1.13	59.60±0.91	60.92±0.69	59.84±0.92	60.84±0.60
	**MCH (pg)**	19.76±0.22	20.46±0.06	19.78±0.36	20.00±0.26	19.76±0.29	19.88±0.19
	**MCHC (g/dL)**	33.12±0.27	32.70±0.50	33.20±0.15	32.84±0.17	33.00±0.28	32.70±0.15
	**RETIC (10^9cells/μL)**	156.9±10.2	170.9±16.1	151.9±14.7	169.0±5.2	143.1±13.7	159.8±7.6
	**PLT (10^3 cells/μL)**	995.6±167.9	858.2±201.0	1208±111	1164±78	1097±56	1217±63
	**WBC (10^3 cells/μL)**	3.48±0.43	4.24±0.97	5.62±1.00	5.88±1.13	4.71±0.35	4.38±0.37
**Clinical biochemical findings**	**TP (g/dL)**	6.10±0.05	6.06±0.14	6.10±0.23	6.04±0.04	6.02±0.09	6.20±0.13
	**ALB (g/dL)**	4.14±0.06	4.02±0.12	3.94±0.19	3.90±0.06	3.90±0.03	4.04±0.09
	**A/G ratio**	2.10±0.05	1.98±0.08	1.84±0.11	1.84±0.06	1.86±0.04	1.88±0.04
	**T-BIL (mg/dL)**	0.07±0.00	0.03±0.00*	0.05±0.01	0.03±0.01*	0.04±0.01	0.04±0.01
	**ALP (U/L)**	354.8±16.1	483.2±45.1	448.2±37.2	433.0±49.5	442.4±44.8	474.6±48.6
	**AST (U/L)**	83.00±2.51	96.60±6.17	92.60±13.1	92.00±2.00	82.00±4.32	84.20±7.04
	**ALT (U/L)**	26.20±0.80	27.00±2.55	31.20±5.00	25.40±2.38	33.20±2.06	29.60±1.96
	**CREA (mg/dL)**	0.43±0.02	0.46±0.03	0.43±0.01	0.41±0.02	0.39±0.01	0.43±0.02
	**BUN (mg/dL)**	19.88±2.57	15.58±1.01	15.94±0.88	13.46±1.22	14.62±0.86	16.86±1.08
	**T-CHO (mg/dL)**	86.80±9.28	85.80±3.81	87.00±4.55	86.60±6.59	86.40±3.82	97.80±9.41
	**TG (mg/dL)**	76.20±19.14	47.60±7.47	48.60±3.66	47.60±16.46	52.40±6.54	63.80±7.65
	**GLU (mg/dL)**	114.0±4.4	124.0±6.5	104.0±2.7	103.6±5.2	113.2±4.6	120.4±6.6
	**CA (mg/dL)**	10.04±0.10	9.96±0.13	10.16±0.20	10.08±0.14	10.28±0.04	10.44±0.10
	**IP (mg/dL)**	7.36±0.25	7.30±0.23	7.48±0.23	7.50±0.22	7.76±0.17	8.00±0.13
	**GGT (IU/L)**	0.34±0.09	0.17±0.07	0.21±0.13	0.32±0.14	0.20±0.13	0.43±0.12
	**CK (U/L)**	606.4±15.2	683.2±67.8	533.6±118.0	491.4±28.3	431.6±45.0	508.6±114.3
	**Na (mmol/L)**	140.8±0.3	141.6±0.5	141.7±0.7	141.9±0.3	141.1±0.3	141.2±0.3
	**K (mmol/L)**	4.44±0.07	4.29±0.14	4.50±0.16	4.44±0.09	4.54±0.12	4.43±0.22
	**Cl (mmol/L)**	100.2±0.3	102.5±0.4*	103.0±0.6**	101.6±0.9	102.3±0.4	101.5±0.3
**ELISA analysis**	**IgE (ng/mL)**	0.222±0.023	0.211±0.008	0.247±0.035	0.211±0.025	0.187±0.022	0.183±0.016
	**Histamine (ng/mL)**	0.253±0.030	0.311±0.043	0.385±0.027	0.257±0.034	0.570±0.128*	0.510±0.063*

Data were presented as mean ± standard error of the mean.

All data were analyzed using Kruskal-Wallis test and the significance of intergroup difference between the control and administered groups was assessed using Dunn's Rank Sum test. All statistical analyses were performed using GraphPad Prism 8.0 (GraphPad Software, Inc., San Diego, CA).

*vs. Control group: $p < 0.05$, ** vs. Control group: $p < 0.01$

Findings of necropsy and histopathology

During the necropsy on all SD rats, no polysorbate 20 or HSA related macroscopic findings were observed such as changes in external appearance, internal organs, and administration sites. In histopathological examination of the administration site, mild macrophage infiltration and neovascularization were observed with a similar degree and frequency in all administered groups, including the control group. In addition, inflammatory cells infiltration, fat infiltration, giant cells, and necrosis were observed only in some individuals regardless of the administered substance and dose (Fig 2).

In the case of the NZW rabbits, macroscopic findings at necropsy and histopathological findings at the sites of administration were not observed regardless of the administered substance (Fig 3).

Fig 2. Histopathological section of the administration site in the four-week repeated intramuscular dose toxicity study in Sprague-Dawley (SD) rats. Hematoxylin and eosin stained histopathological section (original magnification 100x) of the gastrocnemius muscle of the SD rats: a) Control group, male; b) Control group, female; c) Polysorbate 20-0.02 mg/kg, male; d) Polysorbate 20-0.02 mg/kg, female; e) Polysorbate 20-0.1 mg/kg, male; f) Polysorbate 20-0.1 mg/kg, female; g) Polysorbate 20-0.4 mg/kg, male; h) Polysorbate 20-0.4 mg/kg, female; i) Human serum albumin-0.06 mg/kg, male; j) Human serum albumin-0.06 mg/kg, female; k) Human serum albumin-0.12 mg/kg, male; l) Human serum albumin-0.12 mg/kg, female. Mild macrophage infiltration, neovascularization, inflammatory cells infiltration, and fat infiltration were observed with a similar degree and frequency in all administered groups. An arrow pointing up (↑) indicated macrophage infiltration and an arrow pointing down (↓) indicated

neovascularization.

Fig 3. Histopathological section of the administration site in the intradermal irritation study in New Zealand White (NZW) rabbits. Hematoxylin and eosin stained histopathological section (original magnification 50x) of the dorsal back skin of the NZW rabbits: a) Control group; b) Polysorbate 20-0.02 mg/kg; c) Polysorbate 20-0.1 mg/kg; d) Polysorbate 20-0.4 mg/kg; e) Human serum albumin (HSA)-0.06 mg/kg; f) HSA-0.12 mg/kg, male. No histopathological findings were found at the site of administration in all administered groups.

Discussion

Polysorbate 20 was approved by the regulatory authorities as an additive in foods, cosmetics, eye drops, and tablets in various countries [14,15]. Therefore, most clinical and nonclinical safety studies of polysorbate 20 have been conducted using non-invasive routes of administration (e.g., oral, skin, and ophthalmic administration) [16,17]. In particular, as a food additive, polysorbate 20 has various *in vivo* safety data, including *in vitro* genotoxicity and DNA reactivity based on structural alerts [26]. Recently, polysorbate 20, which was one of the excipients of CORETOX® approved by the Ministry of Food and Drug Safety (MFDS, Republic of Korea), has been used in humans via an intramuscular route of administration [12]. However, there are few studies on the safety of polysorbate 20 when administered intramuscularly. In a study conducted in four monkeys, it was reported that intramuscular injection of 275 mg/kg of polysorbate20 for 20 days did not cause toxicity in the liver and kidney [27]. This study did not evaluate clinical signs, body weight, or hematology and clinical biochemistry, which are essential criteria for evaluating toxicity [28]. Therefore, the current study was conducted to verify the

safety of intramuscular administration of polysorbate 20 through four-week repeated intramuscular dose toxicity in SD rats and an intradermal irritation study in NZW rabbits. This study is the first to fully evaluate the *in vivo* safety of polysorbate 20 when administered intramuscularly based on current guidelines of non-clinical safety research.

To evaluate the systemic toxicity of intramuscular injection of polysorbate 20, a four-week repeated dose toxicity study in SD rats was conducted. According to the MFDS, the clinical dosage of CORETOX®, which contains 1 mg of polysorbate 20 in one vial, ranged from 0.2 vial (treatment of forehead wrinkles) to 3.6 vials (stiff muscles in the upper limbs after a stroke) [12]. Thus, the dose (0.02, 0.1, and 0.4 mg/kg) of polysorbate 20 in the current study was established based on the therapeutic indication of CORETOX® in humans and the animal equivalent dose calculation factor [19]. In addition, 0.9% saline was set as the vehicle control substance, while 0.06 and 0.12 mg/kg doses of HSA, which has an *in vivo* safety profile accumulated over the years for intramuscular administration, was set as a comparative substance [2,6].

Generally, mortality, body weight, and clinical signs have the most significance in repeated dose toxicity studies [29,30]. Furthermore, changes in the levels of hematology and clinical biochemistry in laboratory animals could enable more accurate predictions of human toxicity [31,32]. In the current study, adverse effects, including mortality, clinical signs, and macroscopic findings on necropsy, were not found at any dose level of HSA and polysorbate 20 administered during the study period in all SD rats. In addition, significant changes in the body weight and the level of hematology were not observed in the polysorbate 20 and HSA administered groups compared to the control group. In the clinical biochemistry test and allergy reaction test, significant decrease in albumin/globulin ratio ($p < 0.05$), glucose ($p < 0.05$), and total bilirubin ($p < 0.05$) and significant increase in sodium ($p < 0.05$ or $p < 0.01$), chloride ($p < 0.05$ or $p < 0.01$), and serum histamine ($p < 0.05$) were observed in the polysorbate 20 or HSA

administered group compared to the control group. However, these changes had no toxicological significance as they were not dose-responsive and the levels were within the normal range for the same age of SD rats [33,34]. This indicated that intramuscular administration of 0.02, 0.1, and 0.4 mg/kg dose of polysorbate 20 did not cause systemic toxicity in SD rats.

Histopathological examination of the administration site was conducted to observe the local toxicity of the polysorbate 20 in all SD rats. At the administration site, macrophage infiltration, neovascularization, giant cell, fibrosis, and necrosis were observed to a similar degree and frequency in all administered groups regardless of the administered substance. However, these findings were observed in most of the administered groups without a dose response, including the control group. Furthermore, inflammatory lesions could occur in the body of SD rats as a defense mechanism against intramuscular injection of any foreign substances [35]. Therefore, those findings were presumed to be due to the intramuscular injection rather than any effect of polysorbate 20. In other words, there were no toxicological changes at the site of administration after intramuscular administration of polysorbate 20 based on histopathology. Furthermore, serum IgE and histamine levels were measured to determine whether allergic reactions occurred after the administration of polysorbate 20 in all SD rats. In addition to clinical signs, increased levels of serum IgE and histamine are representative indicators of an allergic reaction [36–38]. In the current study, allergic reactions were not observed in response to the administration of polysorbate 20, which indicated that polysorbate 20 is not an allergen in SD rats.

However, despite its very low frequency, side effects in the allergy category have been reported in humans using shampoos (8.4% polysorbate 20, 2 cases among 5.88 million uses), cuticle softeners (2.0% polysorbate 20, 24 cases among 131 million use), and paste masks (2.0% polysorbate 20, 11 cases among 120.7 million uses) containing polysorbate 20 [14,15]. Therefore, an additional intradermal irritation study was conducted in NZW rabbits to confirm that

polysorbate 20 was not an allergen *in vivo*. Traditionally, the intradermal test is the gold standard method for diagnosing allergic reactions [39,40]. If an immune response, such as erythema, crust formation, and edema formation, occurs at the administration site after intradermal administration of a substance, it could be concluded that the substance was an allergen to the administered subjects [24]. In the current study, immune responses of the administration site were not observed at all polysorbate 20 treated NZW rabbits after intradermal administration during the study period, which is the same as the results in the control group and the HSA administered group. Furthermore, in the polysorbate 20 administered group, toxicological changes based on the histopathological examination of the site of administration and increased levels of serum IgE and histamine were not observed. There were also no toxicologically significant changes, such as changes in the body weight or the hematology and clinical biochemistry in all rabbits administered polysorbate 20 and all toxicological values in NZW rabbits were within the normal range for the same age of the NZW rabbits [41,42]. These results of the intradermal irritation study indicated that polysorbate 20 was not an allergen in NZW rabbits, which was consistent with the results of the four-week repeated dose toxicity study in SD rats.

Conclusions

The current study, a four-week repeated intramuscular dose toxicity study in SD rats and an intradermal irritation study in NZW rabbits, provides the basis for the *in vivo* safety profile of intramuscular administration of polysorbate 20, an excipient of newly developed BoNT/A products (CORETOX®). Polysorbate 20, which did not induce systemic and local toxicities in this study, was considered to be a safe substance for intramuscular administration, just like HSA for which a safety profile has been obtained over the years. In addition, since polysorbate 20 is a

synthetic product rather than an animal-derived product, it has the advantages of a consistent quality of the product, ease of manufacture and supply, and a low risk of pathogen transmission compared to HSA. Therefore, the findings of this study provide the scientific reliability of the use of polysorbate 20 as an alternative to HSA, which is used as an excipient for most BoNT/A products, particularly in terms of its safety. Furthermore, this study suggest the possibility that polysorbate 20, which is mainly used as an additive in foods, cosmetics, eye drops, and tablets, could be used as a safe excipient for various pharmaceuticals administered intramuscularly.

Acknowledgments

Not applicable

References

1. Fanali G, Di Masi A, Trezza V, Marino M, Fasano M, Ascenzi P. Human serum albumin: from bench to bedside. Mol Aspects Med. 2012;33: 209–290.
2. Zhou T, Lu S, Liu X, Zhang Y, Xu F. Review of the rational use and adverse reactions to human serum albumin in the People's Republic of China. Patient Prefer Adherence. 2013;7: 1207.
3. Bosse D, Praus M, Kiessling P, Nyman L, Andresen C, Waters J, et al. Phase I comparability of recombinant human albumin and human serum albumin. J Clin Pharmacol. 2005;45: 57–67.
4. Benedetto A V. What's new in cosmetic dermatology. Dermatol Clin. 2019;37: 117–128.
5. Park J-Y, Sunga O, Wanitphakdeedecha R, Frevert J. Neurotoxin Impurities: A Review of Threats to Efficacy. Plast Reconstr Surg Glob Open. 2020;8.
6. Malhotra R, Huilgol SC, Selva D. Botulinum toxin and human serum albumin. Arch Ophthalmol. 2003;121: 1661.
7. Chen Z, He Y, Shi B, Yang D. Human serum albumin from recombinant DNA technology: challenges and strategies. Biochim Biophys Acta (BBA)-General Subj. 2013;1830: 5515–5525.

8. Matejtschuk P, Dash CH, Gascoigne EW. Production of human albumin solution: a continually developing colloid. Br J Anaesth. 2000;85: 887–895.

9. Chamberland ME, Alter HJ, Busch MP, Nemo G, Ricketts M. Emerging infectious disease issues in blood safety. Emerg Infect Dis. 2001;7: 552.

10. Tumber PS, Louis I. Botulinum toxin type a therapy and human serum albumin. Anesthesiol J Am Soc Anesthesiol. 2006;104: 1108.

11. Norsworthy PJ, Thompson AGB, Mok TH, Guntoro F, Dabin LC, Nihat A, et al. A blood miRNA signature associates with sporadic Creutzfeldt-Jakob disease diagnosis. Nat Commun. 2020;11. doi:10.1038/s41467-020-17655-x

12. Lee J, Chun MH, Ko YJ, Lee S-U, Kim DY, Paik N-J, et al. Efficacy and safety of MT10107 (Coretox®) in post-stroke upper limb spasticity treatment: A randomized, double-blind, active drug-controlled, multi-center, phase III clinical trial. Arch Phys Med Rehabil. 2020.

13. Kwak S, Kang W, Rhee C-H, Yang G-H, Cruz DJM. Comparative Pharmacodynamics Study of 3 Different Botulinum Toxin Type A Preparations in Mice. Dermatologic Surg. 2020;46: e132–e138.

14. Moore J. Final report on the safety assessment of polysorbates 20, 21, 40, 60, 61, 65, 80, 81, and 85. J Am Coll Toxicol. 1984;3: 1–82.

15. Lanigan RS. Final report on the safety assessment of PEG-20 sorbitan cocoate; PEG-40 sorbitan diisostearate; PEG-2,-5, and-20 sorbitan isostearate; PEG-40 and-75 sorbitan lanolate; PEG-10,-40,-44,-75, and-80 sorbitan laurate; PEG-3, and-6 sorbitan oleate; PEG-80 sorb. Int J Toxicol. 2000;19: 43–89.

16. Rowe RC, Sheskey P, Quinn M. Handbook of pharmaceutical excipients. Libros Digitales-Pharmaceutical Press; 2009.

17. Lewis RJ, Sax N. Sax's dangerous properties of industrial materials. New York; 1996.

18. Percie du Sert N, Ahluwalia A, Alam S, Avey MT, Baker M, Browne WJ, et al. Reporting animal research: Explanation and elaboration for the ARRIVE guidelines 2.0. PLoS Biol. 2020;18: e3000411.

19. Nair AB, Jacob S. A simple practice guide for dose conversion between animals and human. J basic Clin Pharm. 2016;7: 27.

20. Diehl K, Hull R, Morton D, Pfister R, Rabemampianina Y, Smith D, et al. A good practice guide to the administration of substances and removal of blood, including routes and volumes. J Appl Toxicol An Int J. 2001;21: 15–23.

21. Oh H-M, Park JH, Song DH, Chung ME. Efficacy and safety of a new botulinum toxin type A free of complexing proteins. Toxins (Basel). 2016;8: 4.

22. Aoki KR. A comparison of the safety margins of botulinum neurotoxin serotypes A, B, and F in mice. Toxicon. 2001;39: 1815–1820.

23. Kim S-H, Kim S-B, Yang G-H, Rhee C-H. Mouse compound muscle action potential assay: An alternative method to conduct the LD50 botulinum toxin type A potency test. Toxicon. 2012;60: 341–347.

24. Iso B, STANDARD B. Biological evaluation of medical devices. Part. 2009;1: 10993.

25. Leary SL, Underwood W, Anthony R, Cartner S, Corey D, Grandin T, et al. AVMA guidelines for the euthanasia of animals: 2013 edition. American Veterinary Medical Association Schaumburg, IL; 2013.

26. (ANS) EP on FA and NS added to F. Scientific Opinion on the re- evaluation of polyoxyethylene sorbitan monolaurate (E 432), polyoxyethylene sorbitan monooleate (E 433), polyoxyethylene sorbitan monopalmitate (E 434), polyoxyethylene sorbitan monostearate (E 435) and polyoxyethylene sorbita. EFSA J. 2015;13: 4152.

27. Krantz JC, Carr CJ, Bird JG, Cook S. SUGAR ALCOHOLS: XXVI. Pharmacodynamic studies of polyoxyalkylene derivatives of hexitol anhydride partial fatty acid esters. J Pharmacol Exp Ther. 1948;93: 188–195.

28. Guideline ICHHT. Guidance on nonclinical safety studies for the conduct of human clinical trials and marketing authorization for pharmaceuticals M3 (R2). International conference on harmonisation of technical requirements for registration of pharmaceuticals for human use. 2009.

29. Liu T, Li L, Teng X, Huang X, Liu H, Chen D, et al. Single and repeated dose toxicity of mesoporous hollow silica nanoparticles in intravenously exposed mice. Biomaterials. 2011;32: 1657–1668.

30. Shende P, Kulkarni YA, Gaud RS, Deshmukh K, Cavalli R, Trotta F, et al. Acute and repeated dose toxicity studies of different β- cyclodextrin- based nanosponge formulations. J Pharm Sci. 2015;104: 1856–1863.

31. El Hilaly J, Israili ZH, Lyoussi B. Acute and chronic toxicological studies of Ajuga iva in experimental animals. J Ethnopharmacol. 2004;91: 43–50.

32. Rhiouani H, El-Hilaly J, Israili ZH, Lyoussi B. Acute and sub-chronic toxicity of an aqueous extract of the leaves of Herniaria glabra in rodents. J Ethnopharmacol. 2008;118: 378–386.

33. Lillie LE, Temple NJ, Florence LZ. Reference values for young normal Sprague-Dawley

rats: weight gain, hematology and clinical chemistry. Hum Exp Toxicol. 1996;15: 612–616.

34. Petterino C, Argentino-Storino A. Clinical chemistry and haematology historical data in control Sprague-Dawley rats from pre-clinical toxicity studies. Exp Toxicol Pathol. 2006;57: 213–219.

35. Suttie AW. Boorman's Pathology of the Rat. Elsevier; 2015.

36. Wachholz PA, Dearman RJ, Kimber I. Detection of allergen-specific IgE antibody responses. J Immunotoxicol. 2005;1: 189–199.

37. Barbee RA, Brown WG, Kaltenborn W, Halonen M. Allergen skin-test reactivity in a community population sample: correlation with age, histamine skin reactions, and total serum immunoglobulin E. J Allergy Clin Immunol. 1981;68: 15–19.

38. Tanizaki Y, Komagoe H, Morinaga H, Kitani H, Goda Y, Kimura I. Allergen-and anti-IgE-induced histamine release from whole blood. Int Arch Allergy Immunol. 1984;73: 141–145.

39. Ruzicka T, Gerstmeier M, Przybilla B, Ring J. Allergy to local anesthetics: comparison of patch test with prick and intradermal test results. J Am Acad Dermatol. 1987;16: 1202–1208.

40. Wood RA, Phipatanakul W, Hamilton RG, Eggleston PA. A comparison of skin prick tests, intradermal skin tests, and RASTs in the diagnosis of cat allergy. J Allergy Clin Immunol. 1999;103: 773–779.

41. Gallego M. Laboratory reference intervals for systolic blood pressure, rectal temperature, haematology, biochemistry and venous blood gas and electrolytes in healthy pet rabbits.

Open Vet J. 2017;7: 203–207.

42. Hewitt CD, Innes DJ, Savory J, Wills MR. Normal biochemical and hematological values in New Zealand white rabbits. Clin Chem. 1989;35: 1777–1779.

Supporting information

S1 Table. Individual body weights of male (n: 36) and female (n: 30) Sprague-Dawley (SD) rats in the current study.

S2 Table. Individual body weights of male New Zealand White (NZW) rabbits (n: 18) in the current study.

S3 Table. Individual levels of hematology, clinical biochemistry, and serum IgE and histamine in Sprague-Dawley (SD) rats in the current study.

S4 Table. Individual level of hematology, clinical biochemistry, and serum IgE and histamine in New Zealand White (NZW) rabbits in the current study.

Dear editor and reviewers,

We would like to thank the editor and the reviewers for helpful and constructive comments and critiques. We have done our best to revise the manuscript in order to satisfactorily address each of the points raised. We believe that this revised manuscript has greatly benefited from these changes and hope that it will be considered satisfactory for publication in PLOS ONE.

Editor's comment

- We are grateful to the editor for the comments and suggestions, which have helped us to improve our manuscript. We have revised the manuscript based on the editor's comments and have provided our point-by-point responses to each comment below.

1. Why did you measure IgE levels and not for example HSA/PS specific IgG?

- Thank you for your comments. Generally, allergic reactions are divided into four types (Type I, II, III, and IV), of which type I and IV are the most common types of drug-related allergy (#1). Therefore, we conducted *in vivo* test, a standard method, to determine whether polysorbate 20 induces type I or IV allergies. Then, we confirmed that there was no allergic reaction, including abnormalities at the site of administration, due to polysorbate 20 in both studies (four-week repeated intramuscular dose toxicity study in SD rats and intradermal irritation study in NZW rabbits). After *in vivo* test, since only IgE-mediated anaphylaxis cases (type I allergy) were reported due to polysorbates (#2), we further measured the levels of serum IgE and histamine, which were type I allergy-related factors. The level of IgE and histamine also did not increase after administration of polysorbate 20 in both studies.
- #1: Böhm R, Proksch E, Schwarz T, Cascorbi I. Drug hypersensitivity: diagnosis, genetics, and prevention. Dtsch Arztebl Int. 2018;115: 501.
- #2: Maggio E. Polysorbates, biotherapeutics, and anaphylaxis: a review. Bioprocess Int. 2017.

2. What was the rationale for using the gastrocnemius muscle for injection rather than the thigh muscles?

- We thank you for pointing this out. In the case of BOTOX®, the first FDA-approved Botulinum toxin serotype A (BoNT/A) product, the route of administration was set to gastrocnemius muscle rather than thigh muscle in the toxicity study in rodents (Biologic license application number: 103000). In addition, in the compound muscle action potential (CMAP) and digit abduction scoring (DAS) techniques, which are generally used to evaluate the effectiveness of BoNT/A products, the route of administration was also set to gastrocnemius muscle rather than thigh muscle in the toxicity study in rodents (#1, 2, 3). Therefore, in the current study, the administration route was set to the gastrocnemius muscle for accurate comparison with the previously reported results. We have added the rationale for setting the route of administration to the revised manuscript.
- #1: Kim S-H, Kim S-B, Yang G-H, Rhee C-H. Mouse compound muscle action potential assay: An alternative method to conduct the LD50 botulinum toxin type A potency test. Toxicon. 2012;60: 341–347.

#2: Aoki KR. A comparison of the safety margins of botulinum neurotoxin serotypes A, B, and F in mice. Toxicon. 2001;39: 1815–1820.

#3: Oh H-M, Park JH, Song DH, Chung ME. Efficacy and safety of a new botulinum toxin type A free of complexing proteins. Toxins (Basel). 2016;8: 4.

Original version	Revised version<br>(the revised parts are indicated in red font)
**Line 130-132**<br>All substances were injected once every two weeks for four weeks (a total of 3 times; days 1, 15, 29) into one site of the gastrocnemius muscle of the right hindlimb.	**Line 151-154**<br>All substances were injected once every two weeks for four weeks (a total of 3 times; days 1, 15, 29) into one site of the gastrocnemius muscle of the right hindlimb. The dosing schedule (interval and frequency) was set based on the clinical application schedule (total 1 time, at least three month dosing interval) and the administration site (gastrocnemius muscle) was selected based on the site generally used to evaluate the effectiveness of BoNT/A in rodents [12,21–23].<br><br>Updated references<br>12. Lee J, Chun MH, Ko YJ, Lee S-U, Kim DY, Paik N-J, et al. Efficacy and safety of MT10107 (Coretox®) in post-stroke upper limb spasticity treatment: A randomized, double-blind, active drug-controlled, multi-center, phase III clinical trial. Arch Phys Med Rehabil. 2020.<br>21. Oh H-M, Park JH, Song DH, Chung ME. Efficacy and safety of a new botulinum toxin type A free of complexing proteins. Toxins (Basel). 2016;8: 4.<br>22. Aoki KR. A comparison of the safety margins of botulinum neurotoxin serotypes A, B, and F in mice. Toxicon. 2001;39: 1815–1820.<br>23. Kim S-H, Kim S-B, Yang G-H, Rhee C-H. Mouse compound muscle action potential assay: An alternative method to conduct the LD50

	botulinum toxin type A potency test. Toxicon. 2012;60: 341–347.

3. We recommend rephrasing Lines 202-203 to removed the toxicologists' bias of looking for death in a study ("all animals survived..") to "..all animals remained well throughout the study and showed no clinical signs of disease, pain or distress.". Please also replace uses of the word 'symptoms' (what a patient reports) instead of 'signs' (what is observable).

- Thank you for your recommendation. As you suggest, we have revised the phrase “all SD rats survived throughout the study period” in the revised manuscript. In addition, we have modified the word ‘symptoms’ to ‘signs’ throughout the revised manuscript.

Original version	Revised version<br>(the revised parts are indicated in red font)
**Line 202-204**<br>In the four-week repeated intramuscular dose toxicity study, all SD rats survived throughout the study period. In addition, no specific clinical signs were observed in all animals, including no symptoms at the site of administration.	**Line 226-227**<br>In the four-week repeated intramuscular dose toxicity study, all SD rats remained well throughout the study and showed no clinical signs of disease, pain or distress.
**Line 310-311**<br>This study did not evaluate clinical symptoms ~.	**Line 333-335**<br>This study did not evaluate clinical signs ~.
**Line 325-326**<br>Generally, mortality, body weight, and clinical symptoms ~.	**Line 349-350**<br>Generally, mortality, body weight, and clinical signs ~.
**Line 327-330**<br>In the current study, adverse effects, including mortality, clinical symptoms ~.	**Line 352-354**<br>In the current study, adverse effects, including mortality, clinical signs ~.
**Line 349-350**<br>In addition to clinical symptoms ~.	**Line375-377**<br>In addition to clinical signs ~.

4. We note that the manuscript focuses mainly on the implications of this study for the development of cosmetics, and therefore request that you explicitly assert and emphasize the medical implications of this study in order to show the full range of applications of your findings.

- Thank you for your recommendation. As you suggest, we have modified the “Introduction” and “Conclusion” part accordingly in the revised manuscript. We have emphasized the significance of this study and mentioned the scope of application of our findings in the future.

Original version	Revised version<br>(the revised parts are indicated in red font)
**Line 87-88**	**Line 94-97**<br>It was expected the current study could enable the evaluation of the safety of polysorbate 20 for intramuscular administration and would provide the basis for the *in vivo* safety profile of polysorbate 20 as a pharmaceutical excipient.
**Line 381-384**<br>Therefore, the findings of this study suggest the scientific reliability of the use of polysorbate 20 as an alternative to HSA, which is used as an excipient for most BoNT/A products and various pharmaceuticals, particularly in terms of its safety.	**Line 409-413**<br>Therefore, the findings of this study provide the scientific reliability of the use of polysorbate 20 as an alternative to HSA, which is used as an excipient for most BoNT/A products, particularly in terms of its safety. Furthermore, this study suggest the possibility that polysorbate 20, which is mainly used as an additive in foods, cosmetics, eye drops, and tablets, could be used as a safe excipient for various pharmaceuticals administered intramuscularly.

Journal Requirements

1. Please ensure that your manuscript meets PLOS ONE's style requirements, including those for file naming.

- We have checked that the revised manuscript meets PLOS ONE's style requirements.

2. We note that one or more of the authors are employed by a commercial company. Please provide an amended Funding Statement declaring this commercial affiliation, as well as a statement regarding the Role of Funders in your study. Please also provide an updated Competing Interests Statement declaring this commercial affiliation along with any other relevant declarations relating to employment, consultancy, patents, products in development, or marketed products, etc. Please include both an updated Funding Statement and Competing Interests Statement in your cover letter. We will change the online submission form on your behalf.

- We have revised the Funding Statement and Competing Interests Statement in the revised cover letter. In addition, we have updated author roles in the Author Contributions section of the online submission form and the revised manuscript.

Reviewers' comments

Reviewer #1

Comments are mentioned in the attached pdf file. Although a nice work, statistics needs to be reviewed. A little bit of language errors. Histological images needs to be marked with arrows pointing to the damage or change seen.

- Thank you for your encouraging feedback. Based on the comments in PDF file, we have revised the manuscript, including language errors and histological images. We have provided our point-by-point responses to each comment below.

1. Line 18: Modify "were" to "have". In addition, only one author can be the corresponding author.
   - Thank you for pointing this out. We have modified the text accordingly in the revised manuscript.
   - According to the PLOS ONE's submission guidelines, the following is stated in relation to the corresponding author: “Only one corresponding author can be designated in the submission system, but this does not restrict the number of corresponding authors that may be listed on the article in the event of publication. Whoever is designated as a corresponding author on the title page of the manuscript file will be listed as such upon publication. Include an email address for each corresponding author listed on the title page of the manuscript." Therefore, we kept two corresponding authors in the current study.

Original version	Revised version<br>(the revised parts are indicated in red font)
**Title page (Line 18)**<br>[&]These authors were contributed equally to this work as corresponding authors	**Title page (Line 29)**<br>[&]These authors contributed equally to this work as corresponding author.

2. Line 25-27, 51-52, 80-81: Modify "few" to "a few".
   - Thank you for pointing this out. We have modified the text accordingly in the revised manuscript.

Original version	Revised version<br>(the revised parts are indicated in red font)
**Line 25-27**<br>~ there are few studies on the safety of polysorbate 20 when administered intramuscularly.	**Line37-39**<br>~ there are a few studies on the safety of polysorbate 20 when administered intramuscularly.
**Line 51-52**	The sentence has been deleted.

~ which has few reports of toxicity and immunogenicity in humans, as an excipient.	
**Line 80-81**<br>There are few studies on the safety of polysorbate 20 administered by other routes ~.	**Line 87-89**<br>However, there are a few studies on the safety of polysorbate 20 administered by other routes, especially intramuscular administration, which is the route of BoNT/A injection.

3. Line 90 "Animals and Facility": Mention the grouping of animals in this section. For example, Animals were divided into 5 groups with 11 animals in each group. Group 1 were administered 0.01 ml of substance x for y days and so on. Kindly do this for both the substances.
   - Thank you for your comments. As you suggest, we have revised the text accordingly in the revised manuscript.

Original version	Revised version<br>(the revised parts are indicated in red font)
-	**Line 111-115**<br>Then, animals were divided into six groups with 11 animals (six male and five female SD rats) in each group. Group 1 (control group) were administered 0.9% saline every two weeks. Group 2, 3, and 4 were administered 0.02, 0.1, and 0.4 mg/kg of polysorbate 20 every two weeks, respectively. Group 5 and 6 were administered 0.06 and 0.12 mg/kg of HSA every two weeks, respectively.
-	**Line 122-125**<br>Then, animals were divided into six groups with 3 male NZW rabbits in each group. Group 1 (control group) were administered 0.9% saline once. Group 2, 3, and 4 were administered 0.02, 0.1, and 0.4 mg/kg/site of polysorbate 20 once, respectively. Group 5 and 6 were administered 0.06 and 0.12 mg/kg/site of HSA once, respectively.

4. Line 95, 101: Provide weight range of animals
   - Thank you for your comments. As you suggest, we have revised the text accordingly in the revised manuscript.

Original version	Revised version<br>(the revised parts are indicated in red font)
**Line 94-96**<br>~ a total of 66 SD rats, consisting of 30 females and 36 males, were used (Table 1).	**Line 105-107**<br>~ a total of 66 SD rats, consisting of 30 females (130.7 ~ 146.2g) and 36 males (163.5 ~ 187.2g), were used (Table 1).
**Line 101-102**<br>~ a total of 18 male NZW rabbits were used (Table 1).	**Line 116-117**<br>~ a total of 18 male NZW rabbits (2307.7 ~ 2947.8 g) were used (Table 1).

5. Line 101: Females are considered to be more sensitive. It would have been better if female animals were included. It will help in determining if there are any sex related differences.
   - Thank you for pointing this out. In the ISO guideline, the following is stated in relation to the intradermal irritation study (#1): "Healthy young adult albino rabbits of either sex from a single strain, weighing not less than 2 kg, shall be used. A minimum of three animals shall initially be used to evaluate the test material. Inject intracutaneously 0,2 ml of the extract obtained with polar or non-polar solvent at five sites on one side of each rabbit." Since the test substance was administered to five sites on the back skin, we selected a male as a test system, which was expected to have less interference between the administered substances because it is larger than that of female. In the current study, since there was no reaction by the administration of polysorbate 20 in the male SD rats, it is expected that there will be no side effects by the administration of polysorbate 20 in female SD rats.
   - #1: Iso B, STANDARD B. Biological evaluation of medical devices. Part. 2009;1: 10993.

6. Were the rats and rabbits placed in the same animal room or different ones? Since the temperatures are different, is it that the mentioned temperatures are required for their survival?
   - Thank you for your comments. The four-week repeated intramuscular dose toxicity study in SD rats were conducted in Medytox R&D Center (Republic of Korea) and all SD rats were purchased from Orient Bio, Inc. (Republic of Korea). The intradermal irritation study in NZW rabbits were performed at KTR (Republic of Korea), and all NZW rabbits were purchased from DooYeol Biotech (Republic of Korea). SD rats and NZW rabbits used in these two studies were placed in different facilities. In addition, the overall environment of the facilities for SD rats and NZW rabbits was optimally set based on the guidelines (#1 and #2)
   - #1: ARRP. Guideline 20: Guidelines for the Housing of Rats in Scientific Institutions. Animal Research Review Panel. 2007.

- #2: ARRP. Guideline 18: Guidelines for the Housing of Rabbits in Scientific Institutions. Animal Research Review Panel. 2003

7. Line 178-179: Modify “based on” to “according to”.
   - Thank you for pointing this out. We have modified the text accordingly in the revised manuscript.

Original version	Revised version<br>(the revised parts are indicated in red font)
**Line 178-179**<br>~ based on the AVMA Guidelines for the Euthanasia of Animals [26].	**Line 201-202**<br>~ according to the AVMA Guidelines for the Euthanasia of Animals [25].

8. Line 182-183: Delete “using the naked eye”.
   - Thank you for pointing this out. We have modified the text accordingly in the revised manuscript.

Original version	Revised version<br>(the revised parts are indicated in red font)
**Line 182-183**<br>~ injection sites were observed macroscopically using the naked eye.	**Line 205-206**<br>At necropsy, their external appearance, internal organs, and injection sites were observed macroscopically.

9. Line 187-188: Modify “Then, all slides were” to “All slides were then” and “hematoxylin & eosin” to “hematoxylin and eosin”. In addition, delete “at KTR”. Avoid mentioning everytime that it was done at KTR. One time mention will suffice.
   - Thank you for pointing this out. We have modified the text accordingly in the revised manuscript.

Original version	Revised version<br>(the revised parts are indicated in red font)
**Line 187-188**<br>Then, all slides were stained with hematoxylin & eosin and examined microscopically at KTR.	**Line 210-211**<br>All slides were then stained with hematoxylin and eosin and examined microscopically.
**Line 282-284**<br>Hematoxylin & eosin stained~.	**Line 303-305**<br>Hematoxylin and eosin stained~.
**Line 293-294**	**Line 316-317**

Hematoxylin & eosin stained~.	Hematoxylin and eosin stained ~.

10. Line 227-230 "In SD rats, all substances were injected once every two weeks for four weeks (total three times; days 1, 15, 29) into one site of the gastrocnemius muscle of the right hindlimb.": What was the basis for selection of these days for dosing. Why not daily as in acute toxicity studies?
    - Thank you for your comments. The objective of this study was to verify the *in vivo* safety properties of polysorbate 20, an excipient of CORETOX® (newly developed BoNT/A product), when administered intramuscularly. Therefore, the dosing schedule (interval and frequency) was set based on the clinical application schedule of BoNT/A products (#1). The clinical application interval of BOTOX® (first FDA-approved BoNT/A product), which is similar to that of CORETOX®, is once every 3 months (Biologic license application number: 103000). In addition, according to the FDA approval document of BOTOX®, a 4-week repeated dose intramuscular toxicity study in rats was performed. Therefore, we have selected the dosing schedule of polysorbate 20 (once every two weeks for four weeks, total three times; days 1, 15, 29) based on clinical application dosing schedule, information of FDA-approved BoNT/A product, and ICH guideline. Furthermore, we thought that this four-week repeated intramuscular dose toxicity study could sufficiently replace the acute toxicity study. Because the dose of polysorbate 20 in the current study (0.02, 0.1, and 0.4 mg/kg) is more than six times the clinical application dose (0.003 ~ 0.06 mg/kg) (#2 and #3) and polysorbate 20 (with CORETOX®) is administered every 3 months (or single dose) in human, as mentioned above. Since the commented part is the figure legend section, we have modified the text accordingly in the method section.
    - #1: Guideline ICHHT. Guidance on nonclinical safety studies for the conduct of human clinical trials and marketing authorization for pharmaceuticals M3 (R2). International conference on harmonisation of technical requirements for registration of pharmaceuticals for human use. 2009.
    - #2: Lee J, Chun MH, Ko YJ, Lee S-U, Kim DY, Paik N-J, et al. Efficacy and safety of MT10107 (Coretox®) in post-stroke upper limb spasticity treatment: A randomized, double-blind, active drug-controlled, multi-center, phase III clinical trial. Arch Phys Med Rehabil. 2020.
    - #3: Kwak S, Kang W, Rhee C-H, Yang G-H, Cruz DJM. Comparative Pharmacodynamics Study of 3 Different Botulinum Toxin Type A Preparations in Mice. Dermatologic Surg. 2020;46: e132–e138.

Original version	Revised version<br>(the revised parts are indicated in red font)

Line 130-132	Line 151-154
All substances were injected once every two weeks for four weeks (a total of 3 times; days 1, 15, 29) into one site of the gastrocnemius muscle of the right hindlimb.	All substances were injected once every two weeks for four weeks (a total of 3 times; days 1, 15, 29) into one site of the gastrocnemius muscle of the right hindlimb. The dosing schedule (interval and frequency) was set based on the clinical application schedule (total 1 time, at least three month dosing interval) and the administration site (gastrocnemius muscle) was selected based on the site generally used to evaluate the effectiveness of BoNT/A in rodents [12,21–23].<br><br>Updated references<br>12. Lee J, Chun MH, Ko YJ, Lee S-U, Kim DY, Paik N-J, et al. Efficacy and safety of MT10107 (Coretox®) in post-stroke upper limb spasticity treatment: A randomized, double-blind, active drug-controlled, multi-center, phase III clinical trial. Arch Phys Med Rehabil. 2020.<br>21. Oh H-M, Park JH, Song DH, Chung ME. Efficacy and safety of a new botulinum toxin type A free of complexing proteins. Toxins (Basel). 2016;8: 4.<br>22. Aoki KR. A comparison of the safety margins of botulinum neurotoxin serotypes A, B, and F in mice. Toxicon. 2001;39: 1815–1820.<br>23. Kim S-H, Kim S-B, Yang G-H, Rhee C-H. Mouse compound muscle action potential assay: An alternative method to conduct the LD50 botulinum toxin type A potency test. Toxicon. 2012;60: 341–347.

11. Line 231-232 “In NZW rabbits, all substances were injected once into five sites of the back skin (total one time; day 1).”: What is the basis of dosing selection?
    - Thank you for your comments. As mentioned above (Q5), the following is stated in relation to the intradermal irritation study in the ISO guideline (#1): “Inject intracutaneously

(intradermally) 0,2 ml of the extract obtained with polar or non-polar solvent at five sites on one side of each rabbit." In addition, polysorbate 20 (with CORETOX®) is administered every 3 months (or single dose) in human, as mentioned above (Q10). Therefore, we have selected the dosing method of polysorbate 20 based on the ISO guideline and clinical application dosing schedule. Since the commented part is the figure legend section, we have modified the text accordingly in the method section.

Original version	Revised version<br>(the revised parts are indicated in red font)
**Line 138-139**<br>Then, all substances were injected once into five sites (three left side and two right side) on the back skin (total 1 time; day 1).	**Line 160-162**<br>Then, all substances were injected once into five sites (three left side and two right side) on the back skin (total 1 time; day 1) based on the guideline of the International Organization for Standardization (ISO) [24].<br><br>Updated references<br>28. Iso B, STANDARD B. Biological evaluation of medical devices. Part. 2009;1: 10993.

12. Line 235-236, 332, Table 2 : Modify "hematology, clinical chemistry, serum IgE, and serum histamine" to "hematology, clinical chemistry and serum IgE and histamine". Also, instead of chemistry, I feel the appropriate word used could be clinical biochemistry.
    - Thank you for pointing this out. We have modified the text accordingly in the revised manuscript.

Original version	Revised version<br>(the revised parts are indicated in red font)
**Line 32-34**<br>~ clinical chemistry~ .	**Line 44-46**<br>~ clinical biochemistry ~.
**Line 153**<br>Hematology, clinical chemistry ~.	**Line 176**<br>Hematology, clinical biochemistry ~.
**Line 191-193**<br>~ clinical chemistry level, serum IgE level, and serum histamine levels ~.	**Line 214-216**<br>~ clinical biochemistry level, and serum IgE and histamine levels ~.
**Line 235-236**	**Line 261-262**

~ clinical chemistry, serum IgE, and serum histamine	~ clinical biochemistry, and serum IgE and histamine
**Line 237-238**<br>~ clinical chemistry tests were performed ~.	**Line 263-264**<br>~ clinical biochemistry tests were performed ~.
**Line 240-243**<br>However, in clinical chemistry ~.	**Line 265-269**<br>In clinical biochemistry ~.
**Line 247-249**<br>~ and clinical chemistry parameters ~.	**Line 275-276**<br>~ and clinical biochemistry parameters ~.
**Line 251 (Table 2)**<br>Table 2. The level of hematology, clinical chemistry, serum IgE, and histamine in male Sprague-Dawley (SD) rats in the current study.<br><br>Category-Biochemical findings	**Line 279 (Table 2)**<br>Table 2. The level of hematology, clinical biochemistry, and serum IgE and histamine in male Sprague-Dawley (SD) rats in the current study.<br><br>Category-Clinical biochemical findings
**Line 254 (Table 3)**<br>Table 3. The level of hematology, clinical chemistry, serum IgE, and histamine in female Sprague-Dawley (SD) rats in the current study.<br><br>Category-Biochemical findings	**Line 284 (Table 3)**<br>Table 3. The level of hematology, clinical biochemistry, and serum IgE and histamine in female Sprague-Dawley (SD) rats in the current study.<br><br>Category-Clinical biochemical findings
**Line 310-311**<br>~ clinical chemistry ~	**Line 333-335**<br>~ clinical biochemistry ~.
**Line 326-327**<br>~ clinical chemistry ~.	**Line 350-352**<br>~ clinical biochemistry ~.
**Line 332-334**<br>In the clinical chemistry test ~.	**Line 356-360**<br>In the clinical biochemistry test ~.
**Line 366-369**<br>~ clinical chemistry ~.	**Line 393-396**<br>~ clinical biochemistry ~.
**Line 516-517 (S3 Table)**<br>S3 Table. Individual levels of hematology, clinical chemistry, serum IgE, and histamine in Sprague-Dawley (SD) rats in the current study.	**Line 532-533 (S3 Table)**<br>S3 Table. Individual levels of hematology, clinical biochemistry, and serum IgE and histamine in Sprague-Dawley (SD) rats in the current study.
**Line 518-519 (S4 Table)**	**Line 534-524 (S4 Table)**

S4 Table. Individual levels of hematology, clinical chemistry, serum IgE, and histamine in New Zealand White (NZW) rabbits in the current study.	S4 Table. Individual level of hematology, clinical biochemistry, and serum IgE and histamine in New Zealand White (NZW) rabbits in the current study.

13. Line 239-240: Doses have already been mentioned in methods section. Repetition unnecessary. If any changes were observed, then mention the doses on which changes were observed only.
    - Thank you for pointing this out. We have modified the text accordingly in the revised manuscript.

Original version	Revised version<br>(the revised parts are indicated in red font)
**Line 239-240**<br>In SD rats, there were no polysorbate 20 (0.02, 0.1, and 0.4 mg/kg) or HSA (0.06 and 0.12 mg/kg) related changes in the hematology parameters.	**Line 264-265**<br>In both male and female SD rats, there were no polysorbate 20 or HSA related changes in the hematology parameters.

14. Line 241, 244: Significanlty higher or lower, please mention. In addition, modify “group administered” to “group that were administered”.
    - Thank you for pointing this out. We have modified the text accordingly in the revised manuscript.

Original version	Revised version<br>(the revised parts are indicated in red font)
**Line 240-243**<br>However, in clinical chemistry, the levels of albumin/globulin ratio, total bilirubin, glucose, sodium, and chloride were significantly different in the polysorbate 20 or HSA administered groups compared to the vehicle control group (Tables 2 and 3). Furthermore, in the allergic reaction test (ELISA analysis of serum IgE and histamine), significantly higher serum histamine levels were found in the female group administered HSA at a dose of 0.06 and	**Line 265-274**<br>In clinical biochemistry, compared to the control group, the albumin/globulin ratio was significantly decreased in male group 6 ($p < 0.05$), the level of glucose was significantly decreased in male group 3, 5, and 6 ($p < 0.05$, respectively), and the level of sodium was significantly increased in male group 4 ($p < 0.01$) and 6 ($p < 0.05$) (Table 2). In females, the level of total bilirubin was significantly decreased in group 2 and 4 ($p < 0.05$, respectively) and the level of chloride was significantly increased in group 2 ($p < 0.05$) and 3 ($p < 0.01$),

0.12 mg/kg compared to the vehicle control group (Table 3, Kruskal-Wallis test, $p < 0.05$).	compared to the control group (Table 3). Furthermore, in the allergic reaction test (ELISA analysis of serum IgE and histamine), significantly higher serum histamine levels were found in the female group 5 and 6 compared to the control group ($p < 0.05$).

15. Line 242, 245, 284, 294, 342, 363: Delete "Vehicle". Mention in the beginning of the manuscript that control group was administered the vehicle only. And then just mention control in all the places in the manuscript.
    - Thank you for pointing this out. We have modified the text accordingly in the revised manuscript.

Original version	Revised version<br>(the revised parts are indicated in red font)
**Table 1**<br>Substance – Vehicle (0.9% Saline)	**Table 1**<br>Substance – Control (0.9% Saline)
**Line 217-218**<br>~ compared to the vehicle control group at any of the time points.	**Line 241-242**<br>~ compared to the control group at any of the time points.
**Line 222-223**<br>~ compared to the vehicle control group at any of the time points.	**Line 246-248**<br>~ compared to the control group at any of the time points.
**Line 242**<br>~ compared to the vehicle control group (Tables 2 and 3).	**Line 265-272**<br>~compared to the control group, ~ (Table 2). ~ compared to the control group (Table 3).
**Line 245**<br>~ compared to the vehicle control group (Table 3, Kruskal-Wallis test, $p < 0.05$).	**Line 272-274**<br>~ compared to the control group ($p < 0.05$).
**Line 250**<br>~ compared to the vehicle control group.	**Line 276-278**<br>~ compared to the control group.
**Line 252 (Table 2)**<br>*vs. Vehicle Control group: $p < 0.05$, ** vs. Vehicle Control group: $p < 0.01$	**Line 283 (Table 2)**<br>*vs. Control group: $p < 0.05$, ** vs. Control group: $p < 0.01$
**Line 255 (Table 3)**	**Line 289 (Table 3)**

*vs. Vehicle Control group: $p < 0.05$, ** vs. Vehicle Control group: $p < 0.01$	*vs. Control group: $p < 0.05$, ** vs. Control group: $p < 0.01$
**Line 259-268**<br>~ including the vehicle control group.	**Line 293-295**<br>~ including the control group.
**Line 283-284 (Fig 2)**<br>a) Vehicle control group, male; b) Vehicle control group, female; ~	**Line 305 (Fig 2)**<br>a) Control group, male; b) Control group, female; ~
**Line 294 (Fig 3)**<br>a) Vehicle control group; ~	**Line 317 (Fig 3)**<br>a) Control group; ~
**Line 330-332**<br>~ compared to the vehicle control group.	**Line 354-356**<br>~ compared to the control group.
**Line 332-334**<br>~ compared to the vehicle control group.	**Line 356-360**<br>~ compared to the control group.
**Line 341-343**<br>~ including the vehicle control group.	**Line 367-369**<br>~ including the control group.
**Line 361-364**<br>~ in the vehicle control group and ~	**Line 388-390**<br>~ in the control group and ~

16. Line 248: If the doses are already mentioned in the methods section, there is no need to mention them again and again. It can be simply mentioned that no changes were seen in all the administered doses. If there is a change, them mention the specific dose at which the change was seen.
    - Thank you for pointing this out. We have modified the text accordingly in the revised manuscript.

Original version	Revised version<br>(the revised parts are indicated in red font)
**Line 247-249**<br>In NZW rabbits, there were no changes in the hematology and clinical chemistry parameters in the polysorbate 20 (0.02, 0.1, and 0.4 mg/kg/site) or HSA (0.06 and 0.12 mg/kg/site) administered animals.	**Line 275-276**<br>In NZW rabbits, there were no changes in the hematology and clinical biochemistry parameters in the polysorbate 20 or HSA administered animals.

17. Line 253 and 256: Why arent the data represented as mean=/- standard error of mean? Furthermore, ANOVA or Kruskal-Wallis test are usually followed by a post hoc test such as Dunnet's test, Bonferoni's test or Tukey's test. However, none of the tests have been done to make comparasions. Authors kindly perform a test which best suits your data represented to make comparasions between

test and control values and one test and another test values. Remove the shadings, significant values can be represented by a superscript *

- Thank you for pointing this out. The Kruskal-Wallis test performed to compare toxicological indicators in the current study is a ranking-based nonparametric statistical test. Therefore, we presented the median and range in Tables 2 and 3 to show the data without statistical bias. However, since the raw data of the information presented in Tables 2 and 3 are all presented in S3 table, we have modified Tables 2 and 3 (mean ± standard error of the mean, no shadings) accordingly in the revised manuscript. In addition, the Dunn's Rank Sum test was performed as a post hoc test of Kruskal-Wallis test for the significance of intergroup difference between the control and administered groups. We have modified the text accordingly in the method section.

Original version	Revised version<br>(the revised parts are indicated in red font)
**Line 191-196**<br>The Kruskal-Wallis test was performed to compare toxicological indicators ~.	**Line 219-220**<br>The Kruskal-Wallis test was performed to compare toxicological indicators ~. The significance of intergroup difference between the control and administered groups was assessed using Dunn's Rank Sum test.
**Line 251-253 (Table 2)**<br>Data (Medain [range])<br>Statistically significant items (Kruskal-Wallis test) compared to the vehicle control are shaded gray.	**Line 280-282 (Table 2)**<br>Data were presented as mean ± standard error of the mean.<br>All data were analyzed using Kruskal-Wallis test and the significance of intergroup difference between the control and administered groups was assessed using Dunn's Rank Sum test. All statistical analyses were performed using GraphPad Prism 8.0 (GraphPad Software, Inc., San Diego, CA).
**Line 254-256 (Table 3)**<br>Data (Medain [range])<br>Statistically significant items (Kruskal-Wallis test) compared to the vehicle control are shaded gray.	**Line 286-289 (Table 3)**<br>Data were presented as mean ± standard error of the mean.<br>All data were analyzed using Kruskal-Wallis test and the significance of intergroup difference between the control and administered groups was

	assessed using Dunn's Rank Sum test. All statistical analyses were performed using GraphPad Prism 8.0 (GraphPad Software, Inc., San Diego, CA).

18. Line 257: Modify "in" to "of".
    - Thank you for pointing this out. We have modified the text accordingly in the revised manuscript.

Original version	Revised version<br>(the revised parts are indicated in red font)
**Line 257**<br>Findings in necropsy and histopathology	**Line 290**<br>Findings of necropsy and histopathology

19. Line 258: Modify "polysorbate 20 or HSA related macroscopic findings were not observed ~" to " no polyabsorbate 20 or....... macroscopic findings were observed such as changes in external appearance......".
    - Thank you for pointing this out. We have modified the text accordingly in the revised manuscript.

Original version	Revised version<br>(the revised parts are indicated in red font)
**Line 258-259**<br>During the necropsy on all SD rats, polysorbate 20 or HSA related macroscopic findings were not observed, including in external appearance, internal organs, and administration sites.	**Line 291-293**<br>During the necropsy on all SD rats, no polysorbate 20 or HSA related macroscopic findings were observed such as changes in external appearance, internal organs, and administration sites

20. Line 259-276: Changes observed should be represented as the mean or average changes observed in the whole group, not individual animal changes. Do not mention individual animal changes, rather describe the mean value obtained from the observed group. Don't mention individual rat results, mention what was most commonly observed. Follow this for all the below results too. Please mention only the most common effects seen over all. Please refer some toxicity papers and observe how data are represented.
    - Thank you your recommendations. In the current study, there was no significant histopathological change according to the administered substance in SD rats. Therefore, we have deleted individual changes and only represented the change of the whole groups for the

concise histopathology results. We have modified the text accordingly in the revised manuscript.

Original version	Revised version<br>(the revised parts are indicated in red font)
**Line 259-276**<br>In histopathological examination of the administration site, mild macrophage infiltration (vehicle control group: 6 male and 5 female rats; polysorbate 20-0.02 mg/kg: 6 male and 5 female rats; polysorbate 20-0.1 mg/kg: 4 male and 4 female rats; polysorbate 20-0.4 mg/kg: 6 male and 5 female rats; HSA-0.06 mg/kg: 6 male and 5 female rats; HSA-0.12 mg/kg: 6 male and 5 female rats) and neovascularization (vehicle control group: 6 male and 5 female rats; polysorbate 20-0.02 mg/kg: 4 male and 5 female rats; polysorbate 20-0.1 mg/kg: 3 male and 3 female rats; polysorbate 20-0.4 mg/kg: 4 male and 2 female rats; HSA-0.06 mg/kg: 3 male and 4 female rats; HSA-0.12 mg/kg: 5 male and 4 female rats) were observed with a similar degree and frequency in all administration groups, including the vehicle control group. In addition, inflammatory cells infiltration (1 male rat) and fat infiltration (2 female rats) were observed in the vehicle control group. Among the polysorbate 20 administration group, fat infiltration (3 female rats) was observed in the 0.02 mg/kg group; fat infiltration, giant cells, and necrosis (1 male rat) were observed in the 0.1 mg/kg group; inflammatory cells infiltration (1 male rat) and fat infiltration (1 female rat) were observed in the 0.4 mg/kg group. Among the HSA administration group, inflammatory cells infiltration, fibrosis, fat infiltration, and	**Line 293-297**<br>In histopathological examination of the administration site, mild macrophage infiltration and neovascularization were observed with a similar degree and frequency in all administered groups, including the control group. In addition, inflammatory cells infiltration, fat infiltration, giant cells, and necrosis were observed only in some individuals regardless of the administered substance and dose (Fig 2).

necrosis for 1 male rat, fibrosis for 2 female rats, and inflammatory cells infiltration, fat infiltration, and necrosis for 1 female were observed in the 0.06 mg/kg group; fibrosis (1 male rat) and fat infiltration (1 female rats) were observed in the 0.12 mg/kg group (Fig 2).	

21. Line 267, 364: Modify "administration group" to "administered group".
    - Thank you for pointing this out. We have modified the text accordingly in the revised manuscript.

Original version	Revised version<br>(the revised parts are indicated in red font)
**Line 267-268**<br>~ in all administration groups, including the vehicle control group.	**Line 293-295**<br>~ in all administered groups, including the control group.
**Line 288-290 (Fig 2)**<br>~ in all administration groups.	**Line 309-311 (Fig 2)**<br>~ in all administered groups.
**Line 296-297 (Fig 3)**<br>~ in all administration groups.	**Line 319-320 (Fig 3)**<br>~ in all administered groups.
**Line 333-334**<br>~ in a polysorbate 20 or HSA administration group compared to the vehicle control group.	**Line 356-360**<br>~ in the polysorbate 20 or HSA administered group compared to the control group.
**Line 341**<br>~ in all administration groups regardless of the administered substance.	**Line 365-367**<br>~ in all administered groups regardless of the administered substance.
**Line 364**<br>~ in the vehicle control group and the HSA administration group.	**Line 388-390**<br>~ in the control group and the HSA administered group.
**Lind 364-365**<br>Furthermore, in the polysorbate 20 administration group ~.	**Line 391-393**<br>Furthermore, in the polysorbate 20 administered group ~.

22. Line 332-334: Mention p value. And Modify "a" to "the".
    - Thank you for pointing this out. We have modified the text accordingly in the revised manuscript.

Original version	Revised version (the revised parts are indicated in red font)
**Line 332-334** In the clinical chemistry test and allergy reaction test, significant differences in albumin/globulin ratio, total bilirubin, glucose, sodium, and chloride were observed in a polysorbate 20 or HSA administration group compared to the vehicle control group.	**Line 356-360** In the clinical biochemistry test and allergy reaction test, significant decrease in albumin/globulin ratio (p < 0.05), glucose (p < 0.05), and total bilirubin (p < 0.05) and significant increase in sodium (p < 0.05 or p < 0.01), chloride (p < 0.05 or p < 0.01), and serum histamine (p < 0.05) were observed in the polysorbate 20 or HSA administered group compared to the control group.

23. Figure 2: Histological images needs to be marked with arrows pointing to the damage or change seen.

- Thank you for pointing this out. In Figure 2, macrophage infiltration and neovascularization were seen in all administered group (Fig 2a ~ l). In Figure 3, No histopathological findings were seen in all administered groups (Fig 3a ~ f). Therefore, we have modified the Figure 2 and Figure legend 2 accordingly in the revised manuscript.

Original version	Revised version (the revised parts are indicated in red font)
**Line 288-290** Mild macrophage infiltration, neovascularization, inflammatory cells infiltration, and fat infiltration were observed with a similar degree and frequency in all administration groups.	**Line 309-313** Mild macrophage infiltration, neovascularization, inflammatory cells infiltration, and fat infiltration were observed with a similar degree and frequency in all administered groups. An arrow pointing up (↑) indicated macrophage infiltration and an arrow pointing down (↓) indicated neovascularization.

Reviewers' comments

Reviewer #2

The paper mentioned the use of polysorbate-20 as a replacement of the HSA in the BoNT/A formulation.

- Thank you for your overall comments, which have substantially improved the manuscript. We have provided our point-by-point responses to each comment below.

1. The study mentioned the use of Polysorbate-20 as an excipient for the BoNT products. But no where in the paper any results mention about presence of Botulinum neurotoxin along with polysorbate-20. According to study results polysorbate-20 is nontoxic excipient, and similar as HSA for the any pharmaceutical products administer through transdermal or intramuscular route. Not just limited for the BoNT. Additionally, Polysorbate-20 is already approved exicipient from FDA, so significance of this study is limited.

- Thank you for your comments. Polysorbate 20 is an inactive ingredient for drug products that have already been approved by the FDA for auricular, intramuscular, intravenous, nasal, ophthalmic, oral, subcutaneous, topical, and vaginal administration (Drug Approvals and Databases). However, since it is mainly used as an excipient in tablet and ophthalmic solutions, most of the safety studies on polysorbate 20 have been conducted on oral toxicity and skin irritation (#1 and 2). There are few studies on the safety of polysorbate 20 when administered intramuscularly. Therefore, the current study was conducted to obtain *in vivo* safety data for intramuscular administration of polysorbate 20, which has little data currently accumulated. Findings of the current study suggest that polysorbate 20 could be used as a safe excipient for various pharmaceuticals administered intramuscularly. In addition, BoNT/A product (CORETOX®) that uses polysorbate 20 as an excipient have already been approved by the Ministry of Food and Drug Safety (Republic of Korea) and are on the market. In the approval process, *in vivo* safety and efficacy studies were already conducted in the presence of Botulinum neurotoxin along with polysorbate 20 and the relevant results were submitted to regulatory authorities. Therefore, in the current study, the safety of Botulinum neurotoxin along with polysorbate 20 was not investigated.
- #1: Moore J. Final report on the safety assessment of polysorbates 20, 21, 40, 60, 61, 65, 80, 81, and 85. J Am Coll Toxicol. 1984;3: 1–82.
- #2: Lanigan RS. Final report on the safety assessment of PEG-20 sorbitan cocoate; PEG-40 sorbitan diisostearate; PEG-2,-5, and-20 sorbitan isostearate; PEG-40 and-75 sorbitan lanolate; PEG-10,-40,-44,-75, and-80 sorbitan laurate; PEG-3, and-6 sorbitan oleate; PEG-80 sorb. Int J Toxicol. 2000;19: 43–89.

2. The title as well the introduction should be rewritten without emphasizing the Botulinum toxin. The current data seems the controls used for the formulation study. However, authors can include the possible use of polysorbated-20 as an excipient for Botulinum toxin products in the discussion but should not be only therapeutic product.
    - Thank you for your recommendation. As you suggest, we have revised the title to “Safety verification for polysorbate 20, pharmaceutical excipient for intramuscular administration, in Sprague-Dawley rats and New Zealand White rabbits”. Throughout the ‘Introduction’ part, we have rewritten the manuscript to emphasize polysorbate 20 and HSA rather than Botulinum toxin. In addition, in ‘Introduction’ and ’Conclusions’ part, we have revised the manuscript to emphasize that the findings of the current study could be applied to a various therapeutic product, not limited to BoNT/A product.

Original version	Revised version<br>(the revised parts are indicated in red font)
**Title**<br>Safety verification for polysorbate 20, pharmaceutical excipient of newly developed Botulinum toxin serotype A products, in Sprague-Dawley rats and New Zealand White rabbits	**Title**<br>Safety verification for polysorbate 20, pharmaceutical excipient for intramuscular administration, in Sprague-Dawley rats and New Zealand White rabbits
**Line 41-87 (Introduction)**	**Line 53-97 (Introduction)**<br>Not presented
**Line 82-83**<br>The objective of this study was to verify the *in vivo* safety properties of polysorbate 20, an excipient in a newly developed BoNT/A product, when administered intramuscularly.	**Line 90-91**<br>The objective of this study was to verify the *in vivo* safety properties of polysorbate 20 when administered intramuscularly.
**Line 82-83**<br>-	**Line 94-97**<br>It was expected the current study could enable the evaluation of the safety of polysorbate 20 for intramuscular administration and would provide the basis for the *in vivo* safety profile of polysorbate 20 as a pharmaceutical excipient.
**Line 381-384**	**Line 409-413**

Therefore, the findings of this study suggest the scientific reliability of the use of polysorbate 20 as an alternative to HSA which is used as an excipient for most BoNT/A products and various pharmaceuticals, particularly in terms of its safety	Therefore, the findings of this study provide the scientific reliability of the use of polysorbate 20 as an alternative to HSA, which is used as an excipient for most BoNT/A products, particularly in terms of its safety. Furthermore, this study suggest the possibility that polysorbate 20, which is mainly used as an additive in foods, cosmetics, eye drops, and tablets, could be used as a safe excipient for various pharmaceuticals administered intramuscularly.

3. To use the polysorbated-20 as an excipient in the BoNT based formulation required in depth efficacy, safety, pharmacokinetics and pharmacodynamic study of the Botulinum toxin in the presence of polysorbate-20. The data should be compared with the existing formulation like Botox or dysport etc. The study having BoNT formulation with Polysorbate-20 has already published and mentioned in the paper as a reference # 18 and 19. So the significance of this particular control study reduces.
   - Thank you for your comments. As mentioned in the answer to Q2, *in vivo* safety, efficacy, pharmacodynamic studies were already conducted in the presence of Botulinum neurotoxin along with polysorbate 20 and the relevant results were submitted to regulatory authorities. Considering the characteristics of Botulinum toxin (local diffusion at the injection site, followed by rapid systemic metabolism and excretion), pharmacokinetic studies were not conducted. In those studies, the efficacy and toxicity of BOTOX® (uses HSA as an excipient) and CORETOX® (uses polysorbate 20 as an excipient) were also compared. In addition, since polysorbate 20 is an inactive ingredient, it is considered that efficacy, pharmacokinetics, and pharmacodynamic studies other than safety study are unnecessary when used alone. Therefore, the current study was conducted to obtain *in vivo* safety data of polysorbate 20. Although polysorbate 20 have already been approved by the FDA for the pharmaceutical excipient, the current study is considered worthwhile given the lack of safety data on intramuscular administration of polysorbate 20 (#1 and #2). It is expected that findings of the current study provides the basis for the *in vivo* safety profile of polysorbate 20 administered intramuscularly and the scientific reliability of the use of polysorbate 20 as an excipient for various pharmaceuticals in terms of its safety.

   - #1: Moore J. Final report on the safety assessment of polysorbates 20, 21, 40, 60, 61, 65, 80, 81, and 85. J Am Coll Toxicol. 1984;3: 1–82.

- #2: Lanigan RS. Final report on the safety assessment of PEG-20 sorbitan cocoate; PEG-40 sorbitan diisostearate; PEG-2,-5, and-20 sorbitan isostearate; PEG-40 and-75 sorbitan lanolate; PEG-10,-40,-44,-75, and-80 sorbitan laurate; PEG-3, and-6 sorbitan oleate; PEG-80 sorb. Int J Toxicol. 2000;19: 43–89.

4. Data table 2 and 3 title are same. Please specify if table 2 belong to polysorbate-20 or for HSA.
    - Thank you for your comments. Table 2 shows the level of hematology, clinical biochemistry, and serum IgE and histamine in male SD rats, while Table 3 shows the same values as table 2 in female SD rats.
