# Supplementary material for: Safety verification for polysorbate 20, pharmaceutical excipient for intramuscular administration, in Sprague-Dawley rats and New Zealand White rabbits
Source: PLoS One. 2021 Aug 27;16(8):e0256869. doi: 10.1371/journal.pone.0256869 (PMC8396741; doi:10.1371/journal.pone.0256869)
Supplement: Attachment — Submitted filename: Response to Reviewers.docx [file pone.0256869.s006.docx]

**Dear editor and reviewers,**

We would like to thank the editor and the reviewers for helpful and constructive comments and critiques. We have done our best to revise the manuscript in order to satisfactorily address each of the points raised. We believe that this revised manuscript has greatly benefited from these changes and hope that it will be considered satisfactory for publication in PLOS ONE.

**Editor’s comment**

- We are grateful to the editor for the comments and suggestions, which have helped us to improve our manuscript. We have revised the manuscript based on the editor’s comments and have provided our point-by-point responses to each comment below.

1. Why did you measure IgE levels and not for example HSA/PS specific IgG?

- Thank you for your comments. Generally, allergic reactions are divided into four types (Type I, II, III, and IV), of which type I and IV are the most common types of drug-related allergy (#1). Therefore, we conducted *in vivo* test, a standard method, to determine whether polysorbate 20 induces type I or IV allergies. Then, we confirmed that there was no allergic reaction, including abnormalities at the site of administration, due to polysorbate 20 in both studies (four-week repeated intramuscular dose toxicity study in SD rats and intradermal irritation study in NZW rabbits). After *in vivo* test, since only IgE-mediated anaphylaxis cases (type I allergy) were reported due to polysorbates (#2), we further measured the levels of serum IgE and histamine, which were type I allergy-related factors. The level of IgE and histamine also did not increase after administration of polysorbate 20 in both studies.
- #1: Böhm R, Proksch E, Schwarz T, Cascorbi I. Drug hypersensitivity: diagnosis, genetics, and prevention. Dtsch Arztebl Int. 2018;115: 501.
- #2: Maggio E. Polysorbates, biotherapeutics, and anaphylaxis: a review. Bioprocess Int. 2017.

2. What was the rationale for using the gastrocnemius muscle for injection rather than the thigh muscles?

- We thank you for pointing this out. In the case of BOTOX^®^, the first FDA-approved Botulinum toxin serotype A (BoNT/A) product, the route of administration was set to gastrocnemius muscle rather than thigh muscle in the toxicity study in rodents (Biologic license application number: 103000). In addition, in the compound muscle action potential (CMAP) and digit abduction scoring (DAS) techniques, which are generally used to evaluate the effectiveness of BoNT/A products, the route of administration was also set to gastrocnemius muscle rather than thigh muscle in the toxicity study in rodents (#1, 2, 3). Therefore, in the current study, the administration route was set to the gastrocnemius muscle for accurate comparison with the previously reported results. We have added the rationale for setting the route of administration to the revised manuscript.
- #1: Kim S-H, Kim S-B, Yang G-H, Rhee C-H. Mouse compound muscle action potential assay: An alternative method to conduct the LD50 botulinum toxin type A potency test. Toxicon. 2012;60: 341–347.

#2: Aoki KR. A comparison of the safety margins of botulinum neurotoxin serotypes A, B, and F in mice. Toxicon. 2001;39: 1815–1820.

#3: Oh H-M, Park JH, Song DH, Chung ME. Efficacy and safety of a new botulinum toxin type A free of complexing proteins. Toxins (Basel). 2016;8: 4.

| Original version | Revised version  (the revised parts are indicated in red font) |
| --- | --- |
| **Line 130-132**  All substances were injected once every two weeks for four weeks (a total of 3 times; days 1, 15, 29) into one site of the gastrocnemius muscle of the right hindlimb. | **Line 151-154**  All substances were injected once every two weeks for four weeks (a total of 3 times; days 1, 15, 29) into one site of the gastrocnemius muscle of the right hindlimb. The dosing schedule (interval and frequency) was set based on the clinical application schedule (total 1 time, at least three month dosing interval) and the administration site (gastrocnemius muscle) was selected based on the site generally used to evaluate the effectiveness of BoNT/A in rodents [12,21–23].  Updated references  12. Lee J, Chun MH, Ko YJ, Lee S-U, Kim DY, Paik N-J, et al. Efficacy and safety of MT10107 (Coretox®) in post-stroke upper limb spasticity treatment: A randomized, double-blind, active drug-controlled, multi-center, phase III clinical trial. Arch Phys Med Rehabil. 2020.  21. Oh H-M, Park JH, Song DH, Chung ME. Efficacy and safety of a new botulinum toxin type A free of complexing proteins. Toxins (Basel). 2016;8: 4.  22. Aoki KR. A comparison of the safety margins of botulinum neurotoxin serotypes A, B, and F in mice. Toxicon. 2001;39: 1815–1820.  23. Kim S-H, Kim S-B, Yang G-H, Rhee C-H. Mouse compound muscle action potential assay: An alternative method to conduct the LD50 botulinum toxin type A potency test. Toxicon. 2012;60: 341–347. |

3. We recommend rephrasing Lines 202-203 to removed the toxicologists' bias of looking for death in a study ("all animals survived..") to "..all animals remained well throughout the study and showed no clinical signs of disease, pain or distress.". Please also replace uses of the word 'symptoms' (what a patient reports) instead of 'signs' (what is observable).

- Thank you for your recommendation. As you suggest, we have revised the phrase “all SD rats survived throughout the study period” in the revised manuscript. In addition, we have modified the word ‘symptoms’ to ‘signs’ throughout the revised manuscript.

| Original version | Revised version  (the revised parts are indicated in red font) |
| --- | --- |
| **Line 202-204**  In the four-week repeated intramuscular dose toxicity study, all SD rats survived throughout the study period. In addition, no specific clinical signs were observed in all animals, including no symptoms at the site of administration. | **Line 226-227**  In the four-week repeated intramuscular dose toxicity study, all SD rats remained well throughout the study and showed no clinical signs of disease, pain or distress. |
| **Line 310-311**  This study did not evaluate clinical symptoms ~. | **Line 333-335**  This study did not evaluate clinical signs ~. |
| **Line 325-326**  Generally, mortality, body weight, and clinical symptoms ~. | **Line 349-350**  Generally, mortality, body weight, and clinical signs ~. |
| **Line 327-330**  In the current study, adverse effects, including mortality, clinical symptoms ~. | **Line 352-354**  In the current study, adverse effects, including mortality, clinical signs ~. |
| **Line 349-350**  In addition to clinical symptoms ~. | **Line375-377**  In addition to clinical signs ~. |

4. We note that the manuscript focuses mainly on the implications of this study for the development of cosmetics, and therefore request that you explicitly assert and emphasize the medical implications of this study in order to show the full range of applications of your findings.

- Thank you for your recommendation. As you suggest, we have modified the “Introduction” and “Conclusion” part accordingly in the revised manuscript. We have emphasized the significance of this study and mentioned the scope of application of our findings in the future.

| Original version | Revised version  (the revised parts are indicated in red font) |
| --- | --- |
| **Line 87-88** | **Line 94-97**  It was expected the current study could enable the evaluation of the safety of polysorbate 20 for intramuscular administration and would provide the basis for the *in vivo* safety profile of polysorbate 20 as a pharmaceutical excipient. |
| **Line 381-384**  Therefore, the findings of this study suggest the scientific reliability of the use of polysorbate 20 as an alternative to HSA, which is used as an excipient for most BoNT/A products and various pharmaceuticals, particularly in terms of its safety. | **Line 409-413**  Therefore, the findings of this study provide the scientific reliability of the use of polysorbate 20 as an alternative to HSA, which is used as an excipient for most BoNT/A products, particularly in terms of its safety. Furthermore, this study suggest the possibility that polysorbate 20, which is mainly used as an additive in foods, cosmetics, eye drops, and tablets, could be used as a safe excipient for various pharmaceuticals administered intramuscularly. |

**Journal Requirements**

1. Please ensure that your manuscript meets PLOS ONE's style requirements, including those for file naming.

- We have checked that the revised manuscript meets PLOS ONE's style requirements.

2. We note that one or more of the authors are employed by a commercial company. Please provide an amended Funding Statement declaring this commercial affiliation, as well as a statement regarding the Role of Funders in your study. Please also provide an updated Competing Interests Statement declaring this commercial affiliation along with any other relevant declarations relating to employment, consultancy, patents, products in development, or marketed products, etc. Please include both an updated Funding Statement and Competing Interests Statement in your cover letter. We will change the online submission form on your behalf.

- We have revised the Funding Statement and Competing Interests Statement in the revised cover letter. In addition, we have updated author roles in the Author Contributions section of the online submission form and the revised manuscript.

**Reviewers' comments**

**Reviewer #1**

Comments are mentioned in the attached pdf file. Although a nice work, statistics needs to be reviewed. A little bit of language errors. Histological images needs to be marked with arrows pointing to the damage or change seen.

- Thank you for your encouraging feedback. Based on the comments in PDF file, we have revised the manuscript, including language errors and histological images. We have provided our point-by-point responses to each comment below.

1. Line 18: Modify "were" to "have". In addition, only one author can be the corresponding author.

- Thank you for pointing this out. We have modified the text accordingly in the revised manuscript.
- According to the PLOS ONE's submission guidelines, the following is stated in relation to the corresponding author: “Only one corresponding author can be designated in the submission system, but this does not restrict the number of corresponding authors that may be listed on the article in the event of publication. Whoever is designated as a corresponding author on the title page of the manuscript file will be listed as such upon publication. Include an email address for each corresponding author listed on the title page of the manuscript." Therefore, we kept two corresponding authors in the current study.

| Original version | Revised version  (the revised parts are indicated in red font) |
| --- | --- |
| **Title page (Line 18)**  ^&^These authors were contributed equally to this work as corresponding authors | **Title page (Line 29)**  ^&^These authors contributed equally to this work as corresponding author. |

2. Line 25-27, 51-52, 80-81: Modify "few" to "a few".

- Thank you for pointing this out. We have modified the text accordingly in the revised manuscript.

| Original version | Revised version  (the revised parts are indicated in red font) |
| --- | --- |
| **Line 25-27**  ~ there are few studies on the safety of polysorbate 20 when administered intramuscularly. | **Line37-39**  ~ there are a few studies on the safety of polysorbate 20 when administered intramuscularly. |
| **Line 51-52**  ~ which has few reports of toxicity and immunogenicity in humans, as an excipient. | The sentence has been deleted. |
| **Line 80-81**  There are few studies on the safety of polysorbate 20 administered by other routes ~. | **Line 87-89**  However, there are a few studies on the safety of polysorbate 20 administered by other routes, especially intramuscular administration, which is the route of BoNT/A injection. |

3. Line 90 “Animals and Facility”: Mention the grouping of animals in this section. For example, Animals were divided into 5 groups with 11 animals in each group. Group 1 were administered 0.01 ml of substance x for y days and so on. Kindly do this for both the substances.

- Thank you for your comments. As you suggest, we have revised the text accordingly in the revised manuscript.

| Original version | Revised version  (the revised parts are indicated in red font) |
| --- | --- |
| - | **Line 111-115**  Then, animals were divided into six groups with 11 animals (six male and five female SD rats) in each group. Group 1 (control group) were administered 0.9% saline every two weeks. Group 2, 3, and 4 were administered 0.02, 0.1, and 0.4 mg/kg of polysorbate 20 every two weeks, respectively. Group 5 and 6 were administered 0.06 and 0.12 mg/kg of HSA every two weeks, respectively. |
| **-** | **Line 122-125**  Then, animals were divided into six groups with 3 male NZW rabbits in each group. Group 1 (control group) were administered 0.9% saline once. Group 2, 3, and 4 were administered 0.02, 0.1, and 0.4 mg/kg/site of polysorbate 20 once, respectively. Group 5 and 6 were administered 0.06 and 0.12 mg/kg/site of HSA once, respectively. |

4. Line 95, 101: Provide weight range of animals

- Thank you for your comments. As you suggest, we have revised the text accordingly in the revised manuscript.

| Original version | Revised version  (the revised parts are indicated in red font) |
| --- | --- |
| **Line 94-96**  ~ a total of 66 SD rats, consisting of 30 females and 36 males, were used (Table 1). | **Line 105-107**  ~ a total of 66 SD rats, consisting of 30 females (130.7 ~ 146.2g) and 36 males (163.5 ~ 187.2g), were used (Table 1). |
| **Line 101-102**  ~ a total of 18 male NZW rabbits were used (Table 1). | **Line 116-117**  ~ a total of 18 male NZW rabbits (2307.7 ~ 2947.8 g) were used (Table 1). |

5. Line 101: Females are considered to be more sensitive. It would have been better if female animals were included. It will help in determining if there are any sex related differences.

- Thank you for pointing this out. In the ISO guideline, the following is stated in relation to the intradermal irritation study (#1): “Healthy young adult albino rabbits of either sex from a single strain, weighing not less than 2 kg, shall be used. A minimum of three animals shall initially be used to evaluate the test material. Inject intracutaneously 0,2 ml of the extract obtained with polar or non-polar solvent at five sites on one side of each rabbit.” Since the test substance was administered to five sites on the back skin, we selected a male as a test system, which was expected to have less interference between the administered substances because it is larger than that of female. In the current study, since there was no reaction by the administration of polysorbate 20 in the male SD rats, it is expected that there will be no side effects by the administration of polysorbate 20 in female SD rats.
- #1: Iso B, STANDARD B. Biological evaluation of medical devices. Part. 2009;1: 10993.

6. Were the rats and rabbits placed in the same animal room or different ones? Since the temperatures are different, is it that the mentioned temperatures are required for their survival?

- Thank you for your comments. The four-week repeated intramuscular dose toxicity study in SD rats were conducted in Medytox R&D Center (Republic of Korea) and all SD rats were purchased from Orient Bio, Inc. (Republic of Korea). The intradermal irritation study in NZW rabbits were performed at KTR (Republic of Korea), and all NZW rabbits were purchased from DooYeol Biotech (Republic of Korea). SD rats and NZW rabbits used in these two studies were placed in different facilities. In addition, the overall environment of the facilities for SD rats and NZW rabbits was optimally set based on the guidelines (#1 and #2)
- #1: ARRP. Guideline 20: Guidelines for the Housing of Rats in Scientific Institutions. Animal Research Review Panel. 2007.
- #2: ARRP. Guideline 18: Guidelines for the Housing of Rabbits in Scientific Institutions. Animal Research Review Panel. 2003

7. Line 178-179: Modify “based on” to “according to”.

- Thank you for pointing this out. We have modified the text accordingly in the revised manuscript.

| Original version | Revised version  (the revised parts are indicated in red font) |
| --- | --- |
| **Line 178-179**  ~ based on the AVMA Guidelines for the Euthanasia of Animals [26]. | **Line 201-202**  ~ according to the AVMA Guidelines for the Euthanasia of Animals [25]. |

8. Line 182-183: Delete “using the naked eye”.

- Thank you for pointing this out. We have modified the text accordingly in the revised manuscript.

| Original version | Revised version  (the revised parts are indicated in red font) |
| --- | --- |
| **Line 182-183**  ~ injection sites were observed macroscopically using the naked eye. | **Line 205-206**  At necropsy, their external appearance, internal organs, and injection sites were observed macroscopically. |

9. Line 187-188: Modify “Then, all slides were” to “All slides were then” and “hematoxylin & eosin” to “hematoxylin and eosin”. In addition, delete “at KTR”. Avoid mentioning everytime that it was done at KTR. One time mention will suffice.

- Thank you for pointing this out. We have modified the text accordingly in the revised manuscript.

| Original version | Revised version  (the revised parts are indicated in red font) |
| --- | --- |
| **Line 187-188**  Then, all slides were stained with hematoxylin & eosin and examined microscopically at KTR. | **Line 210-211**  All slides were then stained with hematoxylin and eosin and examined microscopically. |
| **Line 282-284**  Hematoxylin & eosin stained~. | **Line 303-305**  Hematoxylin and eosin stained~. |
| **Line 293-294**  Hematoxylin & eosin stained~. | **Line 316-317**  Hematoxylin and eosin stained ~. |

10. Line 227-230 “In SD rats, all substances were injected once every two weeks for four weeks (total three times; days 1, 15, 29) into one site of the gastrocnemius muscle of the right hindlimb.”: What was the basis for selection of these days for dosing. Why not daily as in acute toxicity studies?

- Thank you for your comments. The objective of this study was to verify the *in vivo* safety properties of polysorbate 20, an excipient of CORETOX^®^ (newly developed BoNT/A product), when administered intramuscularly. Therefore, the dosing schedule (interval and frequency) was set based on the clinical application schedule of BoNT/A products (#1). The clinical application interval of BOTOX^®^ (first FDA-approved BoNT/A product), which is similar to that of CORETOX^®^, is once every 3 months (Biologic license application number: 103000). In addition, according to the FDA approval document of BOTOX^®^, a 4-week repeated dose intramuscular toxicity study in rats was performed. Therefore, we have selected the dosing schedule of polysorbate 20 (once every two weeks for four weeks, total three times; days 1, 15, 29) based on clinical application dosing schedule, information of FDA-approved BoNT/A product, and ICH guideline. Furthermore, we thought that this four-week repeated intramuscular dose toxicity study could sufficiently replace the acute toxicity study. Because the dose of polysorbate 20 in the current study (0.02, 0.1, and 0.4 mg/kg) is more than six times the clinical application dose (0.003 ~ 0.06 mg/kg) (#2 and #3) and polysorbate 20 (with CORETOX^®^) is administered every 3 months (or single dose) in human, as mentioned above. Since the commented part is the figure legend section, we have modified the text accordingly in the method section.
- #1: Guideline ICHHT. Guidance on nonclinical safety studies for the conduct of human clinical trials and marketing authorization for pharmaceuticals M3 (R2). International conference on harmonisation of technical requirements for registration of pharmaceuticals for human use. 2009.
- #2: Lee J, Chun MH, Ko YJ, Lee S-U, Kim DY, Paik N-J, et al. Efficacy and safety of MT10107 (Coretox®) in post-stroke upper limb spasticity treatment: A randomized, double-blind, active drug-controlled, multi-center, phase III clinical trial. Arch Phys Med Rehabil. 2020.
- #3: Kwak S, Kang W, Rhee C-H, Yang G-H, Cruz DJM. Comparative Pharmacodynamics Study of 3 Different Botulinum Toxin Type A Preparations in Mice. Dermatologic Surg. 2020;46: e132–e138.

| Original version | Revised version  (the revised parts are indicated in red font) |
| --- | --- |
| **Line 130-132**  All substances were injected once every two weeks for four weeks (a total of 3 times; days 1, 15, 29) into one site of the gastrocnemius muscle of the right hindlimb. | **Line 151-154**  All substances were injected once every two weeks for four weeks (a total of 3 times; days 1, 15, 29) into one site of the gastrocnemius muscle of the right hindlimb. The dosing schedule (interval and frequency) was set based on the clinical application schedule (total 1 time, at least three month dosing interval) and the administration site (gastrocnemius muscle) was selected based on the site generally used to evaluate the effectiveness of BoNT/A in rodents [12,21–23].  Updated references  12. Lee J, Chun MH, Ko YJ, Lee S-U, Kim DY, Paik N-J, et al. Efficacy and safety of MT10107 (Coretox®) in post-stroke upper limb spasticity treatment: A randomized, double-blind, active drug-controlled, multi-center, phase III clinical trial. Arch Phys Med Rehabil. 2020.  21. Oh H-M, Park JH, Song DH, Chung ME. Efficacy and safety of a new botulinum toxin type A free of complexing proteins. Toxins (Basel). 2016;8: 4.  22. Aoki KR. A comparison of the safety margins of botulinum neurotoxin serotypes A, B, and F in mice. Toxicon. 2001;39: 1815–1820.  23. Kim S-H, Kim S-B, Yang G-H, Rhee C-H. Mouse compound muscle action potential assay: An alternative method to conduct the LD50 botulinum toxin type A potency test. Toxicon. 2012;60: 341–347. |

11. Line 231-232 “In NZW rabbits, all substances were injected once into five sites of the back skin (total one time; day 1).”: What is the basis of dosing selection?

- Thank you for your comments. As mentioned above (Q5), the following is stated in relation to the intradermal irritation study in the ISO guideline (#1): “Inject intracutaneously (intradermally) 0,2 ml of the extract obtained with polar or non-polar solvent at five sites on one side of each rabbit.” In addition, polysorbate 20 (with CORETOX^®^) is administered every 3 months (or single dose) in human, as mentioned above (Q10). Therefore, we have selected the dosing method of polysorbate 20 based on the ISO guideline and clinical application dosing schedule. Since the commented part is the figure legend section, we have modified the text accordingly in the method section.

| Original version | Revised version  (the revised parts are indicated in red font) |
| --- | --- |
| **Line 138-139**  Then, all substances were injected once into five sites (three left side and two right side) on the back skin (total 1 time; day 1). | **Line 160-162**  Then, all substances were injected once into five sites (three left side and two right side) on the back skin (total 1 time; day 1) based on the guideline of the International Organization for Standardization (ISO) [24].  Updated references  28. Iso B, STANDARD B. Biological evaluation of medical devices. Part. 2009;1: 10993. |

12. Line 235-236, 332, Table 2 : Modify “hematology, clinical chemistry, serum IgE, and serum histamine” to “hematology, clinical chemistry and serum IgE and histamine”. Also, instead of chemistry, I feel the appropriate word used could be clinical biochemistry.

- Thank you for pointing this out. We have modified the text accordingly in the revised manuscript.

| Original version | Revised version  (the revised parts are indicated in red font) |
| --- | --- |
| **Line 32-34**  ~ clinical chemistry~ . | **Line 44-46**  ~ clinical biochemistry ~. |
| **Line 153**  Hematology, clinical chemistry ~. | **Line 176**  Hematology, clinical biochemistry ~. |
| **Line 191-193**  ~ clinical chemistry level, serum IgE level, and serum histamine levels ~. | **Line 214-216**  ~ clinical biochemistry level, and serum IgE and histamine levels ~. |
| **Line 235-236**  ~ clinical chemistry, serum IgE, and serum histamine | **Line 261-262**  ~ clinical biochemistry, and serum IgE and histamine |
| **Line 237-238**  ~ clinical chemistry tests were performed ~. | **Line 263-264**  ~ clinical biochemistry tests were performed ~. |
| **Line 240-243**  However, in clinical chemistry ~. | **Line 265-269**  In clinical biochemistry ~. |
| **Line 247-249**  ~ and clinical chemistry parameters ~. | **Line 275-276**  ~ and clinical biochemistry parameters ~. |
| **Line 251 (Table 2)**  Table 2. The level of hematology, clinical chemistry, serum IgE, and histamine in male Sprague-Dawley (SD) rats in the current study.  Category-Biochemical findings | **Line 279 (Table 2)**  Table 2. The level of hematology, clinical biochemistry, and serum IgE and histamine in male Sprague-Dawley (SD) rats in the current study.  Category-Clinical biochemical findings |
| **Line 254 (Table 3)**  Table 3. The level of hematology, clinical chemistry, serum IgE, and histamine in female Sprague-Dawley (SD) rats in the current study.  Category-Biochemical findings | **Line 284 (Table 3)**  Table 3. The level of hematology, clinical biochemistry, and serum IgE and histamine in female Sprague-Dawley (SD) rats in the current study.  Category-Clinical biochemical findings |
| **Line 310-311**  ~ clinical chemistry ~ | **Line 333-335**  ~ clinical biochemistry ~. |
| **Line 326-327**  ~ clinical chemistry ~. | **Line 350-352**  ~ clinical biochemistry ~. |
| **Line 332-334**  In the clinical chemistry test ~. | **Line 356-360**  In the clinical biochemistry test ~. |
| **Line 366-369**  ~ clinical chemistry ~. | **Line 393-396**  ~ clinical biochemistry ~. |
| **Line 516-517 (S3 Table)**  S3 Table. Individual levels of hematology, clinical chemistry, serum IgE, and histamine in Sprague-Dawley (SD) rats in the current study. | **Line 532-533 (S3 Table)**  S3 Table. Individual levels of hematology, clinical biochemistry, and serum IgE and histamine in Sprague-Dawley (SD) rats in the current study. |
| **Line 518-519 (S4 Table)**  S4 Table. Individual levels of hematology, clinical chemistry, serum IgE, and histamine in New Zealand White (NZW) rabbits in the current study. | **Line 534-524 (S4 Table)**  S4 Table. Individual level of hematology, clinical biochemistry, and serum IgE and histamine in New Zealand White (NZW) rabbits in the current study. |

13. Line 239-240: Doses have already been mentioned in methods section. Repetition unnecessary. If any changes were observed, then mention the doses on which changes were observed only.

- Thank you for pointing this out. We have modified the text accordingly in the revised manuscript.

| Original version | Revised version  (the revised parts are indicated in red font) |
| --- | --- |
| **Line 239-240**  In SD rats, there were no polysorbate 20 (0.02, 0.1, and 0.4 mg/kg) or HSA (0.06 and 0.12 mg/kg) related changes in the hematology parameters. | **Line 264-265**  In both male and female SD rats, there were no polysorbate 20 or HSA related changes in the hematology parameters. |

14. Line 241, 244: Significanlty higher or lower, please mention. In addition, modify “group administered” to “group that were administered”.

- Thank you for pointing this out. We have modified the text accordingly in the revised manuscript.

| Original version | Revised version  (the revised parts are indicated in red font) |
| --- | --- |
| **Line 240-243**  However, in clinical chemistry, the levels of albumin/globulin ratio, total bilirubin, glucose, sodium, and chloride were significantly different in the polysorbate 20 or HSA administered groups compared to the vehicle control group (Tables 2 and 3). Furthermore, in the allergic reaction test (ELISA analysis of serum IgE and histamine), significantly higher serum histamine levels were found in the female group administered HSA at a dose of 0.06 and 0.12 mg/kg compared to the vehicle control group (Table 3, Kruskal-Wallis test, *p* < 0.05). | **Line 265-274**  In clinical biochemistry, compared to the control group, the albumin/globulin ratio was significantly decreased in male group 6 (*p* < 0.05), the level of glucose was significantly decreased in male group 3, 5, and 6 (*p* < 0.05, respectively), and the level of sodium was significantly increased in male group 4 (*p* < 0.01) and 6 (*p* < 0.05) (Table 2). In females, the level of total bilirubin was significantly decreased in group 2 and 4 (*p* < 0.05, respectively) and the level of chloride was significantly increased in group 2 (*p* < 0.05) and 3 (*p* < 0.01), compared to the control group (Table 3). Furthermore, in the allergic reaction test (ELISA analysis of serum IgE and histamine), significantly higher serum histamine levels were found in the female group 5 and 6 compared to the control group (*p* < 0.05). |

15. Line 242, 245, 284, 294, 342, 363: Delete “Vehicle”. Mention in the beginning of the manuscript that control group was administered the vehicle only. And then just mention control in all the places in the manuscript.

- Thank you for pointing this out. We have modified the text accordingly in the revised manuscript.

| Original version | Revised version  (the revised parts are indicated in red font) |
| --- | --- |
| **Table 1**  Substance – Vehicle (0.9% Saline) | **Table 1**  Substance – Control (0.9% Saline) |
| **Line 217-218**  ~ compared to the vehicle control group at any of the time points. | **Line 241-242**  ~ compared to the control group at any of the time points. |
| **Line 222-223**  ~ compared to the vehicle control group at any of the time points. | **Line 246-248**  ~ compared to the control group at any of the time points. |
| **Line 242**  ~ compared to the vehicle control group (Tables 2 and 3). | **Line 265-272**  ~compared to the control group, ~ (Table 2). ~ compared to the control group (Table 3). |
| **Line 245**  ~ compared to the vehicle control group (Table 3, Kruskal-Wallis test, *p* < 0.05). | **Line 272-274**  ~ compared to the control group (*p* < 0.05). |
| **Line 250**  ~ compared to the vehicle control group. | **Line 276-278**  ~ compared to the control group. |
| **Line 252 (Table 2)**  *vs. Vehicle Control group: *p* < 0.05, ** vs. Vehicle Control group: *p* < 0.01 | **Line 283 (Table 2)**  ^*^vs. Control group: *p* < 0.05, ^**^ vs. Control group: *p* < 0.01 |
| **Line 255 (Table 3)**  *vs. Vehicle Control group: *p* < 0.05, ** vs. Vehicle Control group: *p* < 0.01 | **Line 289 (Table 3)**  ^*^vs. Control group: *p* < 0.05, ^**^ vs. Control group: *p* < 0.01 |
| **Line 259-268**  ~ including the vehicle control group. | **Line 293-295**  ~ including the control group. |
| **Line 283-284 (Fig 2)**  a) Vehicle control group, male; b) Vehicle control group, female; ~ | **Line 305 (Fig 2)**  a) Control group, male; b) Control group, female; ~ |
| **Line 294 (Fig 3)**  a) Vehicle control group; ~ | **Line 317 (Fig 3)**  a) Control group; ~ |
| **Line 330-332**  ~ compared to the vehicle control group. | **Line 354-356**  ~ compared to the control group. |
| **Line 332-334**  ~ compared to the vehicle control group. | **Line 356-360**  ~ compared to the control group. |
| **Line 341-343**  ~ including the vehicle control group. | **Line 367-369**  ~ including the control group. |
| **Line 361-364**  ~ in the vehicle control group and ~ | **Line 388-390**  ~ in the control group and ~ |

16. Line 248: If the doses are already mentioned in the methods section, there is no need to mention them again and again. It can be simply mentioned that no changes were seen in all the administered doses. If there is a change, them mention the specific dose at which the change was seen.

- Thank you for pointing this out. We have modified the text accordingly in the revised manuscript.

| Original version | Revised version  (the revised parts are indicated in red font) |
| --- | --- |
| **Line 247-249**  In NZW rabbits, there were no changes in the hematology and clinical chemistry parameters in the polysorbate 20 (0.02, 0.1, and 0.4 mg/kg/site) or HSA (0.06 and 0.12 mg/kg/site) administered animals. | **Line 275-276**  In NZW rabbits, there were no changes in the hematology and clinical biochemistry parameters in the polysorbate 20 or HSA administered animals. |

17. Line 253 and 256: Why arent the data represented as mean=/- standard error of mean? Furthermore, ANOVA or Kruskal-Wallis test are usually followed by a post hoc test such as Dunnet's test, Bonferoni's test or Tukey's test. However, none of the tests have been done to make comparasions. Authors kindly perform a test which best suits your data represented to make comparasions between test and control values and one test and another test values. Remove the shadings, significant values can be represented by a superscript *

- Thank you for pointing this out. The Kruskal-Wallis test performed to compare toxicological indicators in the current study is a ranking-based nonparametric statistical test. Therefore, we presented the median and range in Tables 2 and 3 to show the data without statistical bias. However, since the raw data of the information presented in Tables 2 and 3 are all presented in S3 table, we have modified Tables 2 and 3 (mean ± standard error of the mean, no shadings) accordingly in the revised manuscript. In addition, the Dunn's Rank Sum test was performed as a post hoc test of Kruskal-Wallis test for the significance of intergroup difference between the control and administered groups. We have modified the text accordingly in the method section.

| Original version | Revised version  (the revised parts are indicated in red font) |
| --- | --- |
| **Line 191-196**  The Kruskal-Wallis test was performed to compare toxicological indicators ~. | **Line 219-220**  The Kruskal-Wallis test was performed to compare toxicological indicators ~. The significance of intergroup difference between the control and administered groups was assessed using Dunn's Rank Sum test. |
| **Line 251-253 (Table 2)**  Data (Medain [range])  Statistically significant items (Kruskal-Wallis test) compared to the vehicle control are shaded gray. | **Line 280-282 (Table 2)**  Data were presented as mean ± standard error of the mean.  All data were analyzed using Kruskal-Wallis test and the significance of intergroup difference between the control and administered groups was assessed using Dunn's Rank Sum test. All statistical analyses were performed using GraphPad Prism 8.0 (GraphPad Software, Inc., San Diego, CA). |
| **Line 254-256 (Table 3)**  Data (Medain [range])  Statistically significant items (Kruskal-Wallis test) compared to the vehicle control are shaded gray. | **Line 286-289 (Table 3)**  Data were presented as mean ± standard error of the mean.  All data were analyzed using Kruskal-Wallis test and the significance of intergroup difference between the control and administered groups was assessed using Dunn's Rank Sum test. All statistical analyses were performed using GraphPad Prism 8.0 (GraphPad Software, Inc., San Diego, CA). |

18. Line 257: Modify "in" to "of".

- Thank you for pointing this out. We have modified the text accordingly in the revised manuscript.

| Original version | Revised version  (the revised parts are indicated in red font) |
| --- | --- |
| **Line 257**  Findings in necropsy and histopathology | **Line 290**  Findings of necropsy and histopathology |

19. Line 258: Modify "polysorbate 20 or HSA related macroscopic findings were not observed ~" to " no polyabsorbate 20 or....... macroscopic findings were observed such as changes in external appearance......".

- Thank you for pointing this out. We have modified the text accordingly in the revised manuscript.

| Original version | Revised version  (the revised parts are indicated in red font) |
| --- | --- |
| **Line 258-259**  During the necropsy on all SD rats, polysorbate 20 or HSA related macroscopic findings were not observed, including in external appearance, internal organs, and administration sites. | **Line 291-293**  During the necropsy on all SD rats, no polysorbate 20 or HSA related macroscopic findings were observed such as changes in external appearance, internal organs, and administration sites |

20. Line 259-276: Changes observed should be represented as the mean or average changes observed in the whole group, not individual animal changes. Do not mention individual animal changes, rather describe the mean value obtained from the observed group. Don’t mention individual rat results, mention what was most commonly observed. Follow this for all the below results too. Please mention only the most common effects seen over all. Please refer some toxicity papers and observe how data are represented.

- Thank you your recommendations. In the current study, there was no significant histopathological change according to the administered substance in SD rats. Therefore, we have deleted individual changes and only represented the change of the whole groups for the concise histopathology results. We have modified the text accordingly in the revised manuscript.

| Original version | Revised version  (the revised parts are indicated in red font) |
| --- | --- |
| **Line 259-276**  In histopathological examination of the administration site, mild macrophage infiltration (vehicle control group: 6 male and 5 female rats; polysorbate 20-0.02 mg/kg: 6 male and 5 female rats; polysorbate 20-0.1 mg/kg: 4 male and 4 female rats; polysorbate 20-0.4 mg/kg: 6 male and 5 female rats; HSA-0.06 mg/kg: 6 male and 5 female rats; HSA-0.12 mg/kg: 6 male and 5 female rats) and neovascularization (vehicle control group: 6 male and 5 female rats; polysorbate 20-0.02 mg/kg: 4 male and 5 female rats; polysorbate 20-0.1 mg/kg: 3 male and 3 female rats; polysorbate 20-0.4 mg/kg: 4 male and 2 female rats; HSA-0.06 mg/kg: 3 male and 4 female rats; HSA-0.12 mg/kg: 5 male and 4 female rats) were observed with a similar degree and frequency in all administration groups, including the vehicle control group. In addition, inflammatory cells infiltration (1 male rat) and fat infiltration (2 female rats) were observed in the vehicle control group. Among the polysorbate 20 administration group, fat infiltration (3 female rats) was observed in the 0.02 mg/kg group; fat infiltration, giant cells, and necrosis (1 male rat) were observed in the 0.1 mg/kg group; inflammatory cells infiltration (1 male rat) and fat infiltration (1 female rat) were observed in the 0.4 mg/kg group. Among the HSA administration group, inflammatory cells infiltration, fibrosis, fat infiltration, and necrosis for 1 male rat, fibrosis for 2 female rats, and inflammatory cells infiltration, fat infiltration, and necrosis for 1 female were observed in the 0.06 mg/kg group; fibrosis (1 male rat) and fat infiltration (1 female rats) were observed in the 0.12 mg/kg group (Fig 2). | **Line 293-297**  In histopathological examination of the administration site, mild macrophage infiltration and neovascularization were observed with a similar degree and frequency in all administered groups, including the control group. In addition, inflammatory cells infiltration, fat infiltration, giant cells, and necrosis were observed only in some individuals regardless of the administered substance and dose (Fig 2). |

21. Line 267, 364: Modify “administration group” to “administered group”.

- Thank you for pointing this out. We have modified the text accordingly in the revised manuscript.

| Original version | Revised version  (the revised parts are indicated in red font) |
| --- | --- |
| **Line 267-268**  ~ in all administration groups, including the vehicle control group. | **Line 293-295**  ~ in all administered groups, including the control group. |
| **Line 288-290 (Fig 2)**  **~** in all administration groups. | **Line 309-311 (Fig 2)**  ~ in all administered groups. |
| **Line 296-297 (Fig 3)**  **~** in all administration groups. | **Line 319-320 (Fig 3)**  ~ in all administered groups. |
| **Line 333-334**  ~ in a polysorbate 20 or HSA administration group compared to the vehicle control group. | **Line 356-360**  ~ in the polysorbate 20 or HSA administered group compared to the control group. |
| **Line 341**  ~ in all administration groups regardless of the administered substance. | **Line 365-367**  ~ in all administered groups regardless of the administered substance. |
| **Line 364**  ~ in the vehicle control group and the HSA administration group. | **Line 388-390**  ~ in the control group and the HSA administered group. |
| **Lind 364-365**  Furthermore, in the polysorbate 20 administration group ~. | **Line 391-393**  Furthermore, in the polysorbate 20 administered group ~. |

22. Line 332-334: Mention p value. And Modify “a” to “the”.

- Thank you for pointing this out. We have modified the text accordingly in the revised manuscript.

| Original version | Revised version  (the revised parts are indicated in red font) |
| --- | --- |
| **Line 332-334**  In the clinical chemistry test and allergy reaction test, significant differences in albumin/globulin ratio, total bilirubin, glucose, sodium, and chloride were observed in a polysorbate 20 or HSA administration group compared to the vehicle control group. | **Line 356-360**  In the clinical biochemistry test and allergy reaction test, significant decrease in albumin/globulin ratio (*p* < 0.05), glucose (*p* < 0.05), and total bilirubin (*p* < 0.05) and significant increase in sodium (*p* < 0.05 or *p* < 0.01), chloride (*p* < 0.05 or *p* < 0.01), and serum histamine (*p* < 0.05) were observed in the polysorbate 20 or HSA administered group compared to the control group. |

23. Figure 2: Histological images needs to be marked with arrows pointing to the damage or change seen.

- Thank you for pointing this out. In Figure 2, macrophage infiltration and neovascularization were seen in all administered group (Fig 2a ~ l). In Figure 3, No histopathological findings were seen in all administered groups (Fig 3a ~ f). Therefore, we have modified the Figure 2 and Figure legend 2 accordingly in the revised manuscript.

| Original version | Revised version  (the revised parts are indicated in red font) |
| --- | --- |
| **Line 288-290**  Mild macrophage infiltration, neovascularization, inflammatory cells infiltration, and fat infiltration were observed with a similar degree and frequency in all administration groups. | **Line 309-313**  Mild macrophage infiltration, neovascularization, inflammatory cells infiltration, and fat infiltration were observed with a similar degree and frequency in all administered groups. An arrow pointing up (↑) indicated macrophage infiltration and an arrow pointing down (↓) indicated neovascularization. |

**Reviewers' comments**

**Reviewer #2**

The paper mentioned the use of polysorbate-20 as a replacement of the HSA in the BoNT/A formulation.

- Thank you for your overall comments, which have substantially improved the manuscript. We have provided our point-by-point responses to each comment below.

1. The study mentioned the use of Polysorbate-20 as an excipient for the BoNT products. But no where in the paper any results mention about presence of Botulinum neurotoxin along with polysorbate-20. According to study results polysorbate-20 is nontoxic excipient, and similar as HSA for the any pharmaceutical products administer through transdermal or intramuscular route. Not just limited for the BoNT. Additionally, Polysorbate-20 is already approved exicipient from FDA, so significance of this study is limited.

- Thank you for your comments. Polysorbate 20 is an inactive ingredient for drug products that have already been approved by the FDA for auricular, intramuscular, intravenous, nasal, ophthalmic, oral, subcutaneous, topical, and vaginal administration (Drug Approvals and Databases). However, since it is mainly used as an excipient in tablet and ophthalmic solutions, most of the safety studies on polysorbate 20 have been conducted on oral toxicity and skin irritation (#1 and 2). There are few studies on the safety of polysorbate 20 when administered intramuscularly. Therefore, the current study was conducted to obtain *in vivo* safety data for intramuscular administration of polysorbate 20, which has little data currently accumulated. Findings of the current study suggest that polysorbate 20 could be used as a safe excipient for various pharmaceuticals administered intramuscularly. In addition, BoNT/A product (CORETOX^®^) that uses polysorbate 20 as an excipient have already been approved by the Ministry of Food and Drug Safety (Republic of Korea) and are on the market. In the approval process, *in vivo* safety and efficacy studies were already conducted in the presence of Botulinum neurotoxin along with polysorbate 20 and the relevant results were submitted to regulatory authorities. Therefore, in the current study, the safety of Botulinum neurotoxin along with polysorbate 20 was not investigated.
- #1: Moore J. Final report on the safety assessment of polysorbates 20, 21, 40, 60, 61, 65, 80, 81, and 85. J Am Coll Toxicol. 1984;3: 1–82.
- #2: Lanigan RS. Final report on the safety assessment of PEG-20 sorbitan cocoate; PEG-40 sorbitan diisostearate; PEG-2,-5, and-20 sorbitan isostearate; PEG-40 and-75 sorbitan lanolate; PEG-10,-40,-44,-75, and-80 sorbitan laurate; PEG-3, and-6 sorbitan oleate; PEG-80 sorb. Int J Toxicol. 2000;19: 43–89.

2. The title as well the introduction should be rewritten without emphasizing the Botulinum toxin. The current data seems the controls used for the formulation study. However, authors can include the possible use of polysorbated-20 as an excipient for Botulinum toxin products in the discussion but should not be only therapeutic product.

- Thank you for your recommendation. As you suggest, we have revised the title to “Safety verification for polysorbate 20, pharmaceutical excipient for intramuscular administration, in Sprague-Dawley rats and New Zealand White rabbits”. Throughout the ‘Introduction’ part, we have rewritten the manuscript to emphasize polysorbate 20 and HSA rather than Botulinum toxin. In addition, in ‘Introduction’ and ’Conclusions’ part, we have revised the manuscript to emphasize that the findings of the current study could be applied to a various therapeutic product, not limited to BoNT/A product.

| Original version | Revised version  (the revised parts are indicated in red font) |
| --- | --- |
| **Title**  Safety verification for polysorbate 20, pharmaceutical excipient of newly developed Botulinum toxin serotype A products, in Sprague-Dawley rats and New Zealand White rabbits | **Title**  Safety verification for polysorbate 20, pharmaceutical excipient for intramuscular administration, in Sprague-Dawley rats and New Zealand White rabbits |
| **Line 41-87 (Introduction)** | **Line 53-97 (Introduction)**  Not presented |
| **Line 82-83**  The objective of this study was to verify the *in vivo* safety properties of polysorbate 20, an excipient in a newly developed BoNT/A product, when administered intramuscularly. | **Line 90-91**  The objective of this study was to verify the *in vivo* safety properties of polysorbate 20 when administered intramuscularly. |
| **Line 82-83**  - | **Line 94-97**  It was expected the current study could enable the evaluation of the safety of polysorbate 20 for intramuscular administration and would provide the basis for the *in vivo* safety profile of polysorbate 20 as a pharmaceutical excipient. |
| **Line 381-384**  Therefore, the findings of this study suggest the scientific reliability of the use of polysorbate 20 as an alternative to HSA which is used as an excipient for most BoNT/A products and various pharmaceuticals, particularly in terms of its safety | **Line 409-413**  Therefore, the findings of this study provide the scientific reliability of the use of polysorbate 20 as an alternative to HSA, which is used as an excipient for most BoNT/A products, particularly in terms of its safety. Furthermore, this study suggest the possibility that polysorbate 20, which is mainly used as an additive in foods, cosmetics, eye drops, and tablets, could be used as a safe excipient for various pharmaceuticals administered intramuscularly. |

3. To use the polysorbated-20 as an excipient in the BoNT based formulation required in depth efficacy, safety, pharmacokinetics and pharmacodynamic study of the Botulinum toxin in the presence of polysorbate-20. The data should be compared with the existing formulation like Botox or dysport etc. The study having BoNT formulation with Polysorbate-20 has already published and mentioned in the paper as a reference # 18 and 19. So the significance of this particular control study reduces.

- Thank you for your comments. As mentioned in the answer to Q2, *in vivo* safety, efficacy, pharmacodynamic studies were already conducted in the presence of Botulinum neurotoxin along with polysorbate 20 and the relevant results were submitted to regulatory authorities. Considering the characteristics of Botulinum toxin (local diffusion at the injection site, followed by rapid systemic metabolism and excretion), pharmacokinetic studies were not conducted. In those studies, the efficacy and toxicity of BOTOX^®^ (uses HSA as an excipient) and CORETOX^®^ (uses polysorbate 20 as an excipient) were also compared. In addition, since polysorbate 20 is an inactive ingredient, it is considered that efficacy, pharmacokinetics, and pharmacodynamic studies other than safety study are unnecessary when used alone. Therefore, the current study was conducted to obtain *in vivo* safety data of polysorbate 20. Although polysorbate 20 have already been approved by the FDA for the pharmaceutical excipient, the current study is considered worthwhile given the lack of safety data on intramuscular administration of polysorbate 20 (#1 and #2). It is expected that findings of the current study provides the basis for the *in vivo* safety profile of polysorbate 20 administered intramuscularly and the scientific reliability of the use of polysorbate 20 as an excipient for various pharmaceuticals in terms of its safety.
- #1: Moore J. Final report on the safety assessment of polysorbates 20, 21, 40, 60, 61, 65, 80, 81, and 85. J Am Coll Toxicol. 1984;3: 1–82.
- #2: Lanigan RS. Final report on the safety assessment of PEG-20 sorbitan cocoate; PEG-40 sorbitan diisostearate; PEG-2,-5, and-20 sorbitan isostearate; PEG-40 and-75 sorbitan lanolate; PEG-10,-40,-44,-75, and-80 sorbitan laurate; PEG-3, and-6 sorbitan oleate; PEG-80 sorb. Int J Toxicol. 2000;19: 43–89.

4. Data table 2 and 3 title are same. Please specify if table 2 belong to polysorbate-20 or for HSA.

- Thank you for your comments. Table 2 shows the level of hematology, clinical biochemistry, and serum IgE and histamine in male SD rats, while Table 3 shows the same values as table 2 in female SD rats.
