# Supplementary material for: Safety verification for polysorbate 20, pharmaceutical excipient for intramuscular administration, in Sprague-Dawley rats and New Zealand White rabbits
Source: PLoS One. 2021 Aug 27;16(8):e0256869. doi: 10.1371/journal.pone.0256869 (PMC8396741; doi:10.1371/journal.pone.0256869)
Supplement: Attachment — Submitted filename: Response to Reviewers.docx [file pone.0256869.s008.docx]

**Dear editor and reviewers,**

We appreciate the editor and the reviewers to review our manuscript. Our manuscript has been remarkably improved due to their valuable comments. Our responses to the comments are provided below.

**Reviewers' comments**

**[Reviewer #1]**

1. If the authors have adequately addressed your comments raised in a previous round of review and you feel that this manuscript is now acceptable for publication, you may indicate that here to bypass the “Comments to the Author” section, enter your conflict of interest statement in the “Confidential to Editor” section, and submit your "Accept" recommendation: **All comments have been addressed.**
2. Is the manuscript technically sound, and do the data support the conclusions?**: Yes**
3. Has the statistical analysis been performed appropriately and rigorously?**: Yes**
4. Have the authors made all data underlying the findings in their manuscript fully available?**: Yes**
5. Is the manuscript presented in an intelligible fashion and written in standard English?**: No**
6. Review Comments to the Author: **All queries have been addressed. An interesting work. Some minor language errors. References require a little bit of checking. The comments are mentioned in the attached pdf file.**

- Thank you for your feedback. We have provided our point-by-point responses to each comment below.

7. Abstract does not contain keywords?

- We have already submitted keywords within the submission system (Botulinum toxin type A products; Pharmaceutical excipient; Polysorbate 20; Human serum albumin). However, we have added keywords in the revised manuscript.

| Original version | Revised version  (the revised parts are indicated in red font) |
| --- | --- |
|  | **Line 52-53**  Keywords: Botulinum toxin type A products, Pharmaceutical excipient, Polysorbate 20, Human serum albumin |

8. Line 57: 19 superscript th

- Thank you for pointing this out. We have modified the text accordingly in the revised manuscript.

| Original version | Revised version  (the revised parts are indicated in red font) |
| --- | --- |
| **Line 57**  Therefore, HSA has been used for therapeutic purposes in patients with hemorrhage, hypovolemia, and hypoalbuminemia since about the 19th century [2]. | **Line 60**  Therefore, HSA has been used for therapeutic purposes in patients with hemorrhage, hypovolemia, and hypoalbuminemia since about the 19^th^ century [2]. |

9. Line 126-127 (Table 1): Number of animals (male/female)

- Thank you for pointing this out. We have modified the text accordingly in the revised manuscript.

| Original version | Revised version  (the revised parts are indicated in red font) |
| --- | --- |
| **Table 1**  No. animals (Male/Female) | **Table 1**  Number of animals (male/female) |

10. Line 131: Which

- Thank you for pointing this out. We have modified the text accordingly in the revised manuscript.

| Original version | Revised version  (the revised parts are indicated in red font) |
| --- | --- |
| **Line 131**  HSA (Green Cross Corp., Republic of Korea) was selected as the comparative substance for the polysorbate 20 (Merck, Germany) that was the test substance of the current study. | **Line 135**  HSA (Green Cross Corp., Republic of Korea) was selected as the comparative substance for the polysorbate 20 (Merck, Germany) which was the test substance of the current study. |

11. Line 137-139: Delete ‘Then’ and ‘manner as when determining the dose of HSA’. Join this sentence (As a result, 0.02, 0.1, and 0.4 mg/kg were selected as the dose of polysorbate 20.) with the earlier sentence.

- Thank you for comments. We have modified the text accordingly in the revised manuscript.

| Original version | Revised version  (the revised parts are indicated in red font) |
| --- | --- |
| **Line 137-139**  Then, the dose of polysorbate 20 was also calculated in the same manner as when determining the dose of HSA. As a result, 0.02, 0.1, and 0.4 mg/kg were selected as the dose of polysorbate 20 | **Line 141-142**  The dose of polysorbate 20 was also calculated in the same manner and 0.02, 0.1, and 0.4 mg/kg were selected as the dose of polysorbate 20. |

12. Line 189, 206: Delete ‘Then’. Please do not start a sentence with then. Kindly correct in other places also.

- Thank you for pointing this out. We have modified the text accordingly in the revised manuscript.

| Original version | Revised version  (the revised parts are indicated in red font) |
| --- | --- |
| **Line 41**  Then, an intradermal irritation study was further conducted with 18 New Zealand White (NZW) rabbits. | **Line 41**  An intradermal irritation study was further conducted with 18 New Zealand White (NZW) rabbits. |
| **Line 111**  Then, animals were divided into six groups with 11 animals (six male and five female SD rats) in each group. | **Line 115**  Animals were divided into six groups with 11 animals (six male and five female SD rats) in each group. |
| **Line 122**  Then, animals were divided into six groups with 3 male NZW rabbits in each group. | **Line 125**  Animals were divided into six groups with 3 male NZW rabbits in each group. |
| **Line 160**  Then, all substances were injected once into five sites (three left side and two right side) on the back skin (total 1 time; day 1) based on the guideline of the International Organization for Standardization (ISO) [24]. | **Line 165**  All substances were then injected once into five sites (three left side and two right side) on the back skin (total 1 time; day 1) based on the guideline of the International Organization for Standardization (ISO) [24]. |
| **Line 178**  Then, the SD rats were anesthetized with isoflurane and blood samples were collected from their abdominal vein. | **Line 183**  The SD rats were anesthetized with isoflurane and blood samples were collected from their abdominal vein. |
| **Line 189**  Then, the following parameters were examined in KTR using an automatic analyzer (TBA-120FR, TOSHIBA, Japan): | **Line 194**  The following parameters were then examined in KTR using an automatic analyzer (TBA-120FR, TOSHIBA, Japan): |
| **Line 206**  Then, the injection site (one site of the gastrocnemius muscle of the right hindlimb for the SD rats and the five sites of the back skin for the NZW rabbits) were collected and stored in 10% neutral buffered formalin. | **Line 211**  The injection site (one site of the gastrocnemius muscle of the right hindlimb for the SD rats and the five sites of the back skin for the NZW rabbits) were collected and stored in 10% neutral buffered formalin. |

13. Line 213: Please mention that data are represented as mean ± standard error of mean (SEM).

- Thank you for comments. We have modified the text accordingly in the revised manuscript.

| Original version | Revised version  (the revised parts are indicated in red font) |
| --- | --- |
|  | **Line 227-228**  Data are represented as mean ± standard error of the mean (SEM). |

14. Line 232: Delete ‘Changes in’.

- Thank you for pointing this out. We have modified the text accordingly in the revised manuscript.

| Original version | Revised version  (the revised parts are indicated in red font) |
| --- | --- |
| **Line 232**  Changes in body weights | **Line 238**  Body weights |

15. Line 235 and 264: In a sentence wherever the word table comes please make the ‘t’ small. While mentioning as a caption or a table then the ‘t’ can be capitalised. Kindly make these corrections in other locations as well.

- Thank you for comments. According to the PLOS ONE's submission guidelines, the following is stated in relation to tables:
- [Table citations] Tables should be cited as “Table 1”, “Table 2”, etc.
- [Examples] Vestibulum adipiscing urna ut lectus gravida, et bland Table 1.
- Therefore, we have kept the existing table citation form.

16. Line 237: Is it Fig. 1a or Fig 1a? Please kindly refer the journal guidelines once.

- Thank you for comments. According to the PLOS ONE's submission guidelines, the following is stated in relation to the figures:
- [Figure citations] Cite figures as “Fig 1”, “Fig 2”, etc.
- [Examples] Vestibulum adipiscing urna ut lectus gravida, vitae (Fig 1) interdum. Nam sit amet nulla lacus a, Figs 1 and 2 ultrices tellus.
- Therefore, we have kept the existing figure citation form.

17. Line 239, 241: “respectively” is inappropriate here, since you have already mentioned the weight immediately against the day. If days were mentioned consecutively followed by weights consecutively, then respectively may be used.

- Thank you for pointing this out. We have modified the text accordingly in the revised manuscript.

| Original version | Revised version  (the revised parts are indicated in red font) |
| --- | --- |
| **Line 237-241**  Compared to the body weight before the first administration, all male SD rats had a body weight of 138.4% at day 8, 173.6% at day 15, 200.4% at day 22, and 225.0% at day 29, respectively, while all female SD rats had a body weight of 132.2% at day 8, 153.1% at day 15, 173.1% at day 22, and 187.3% at day 29, respectively. | **Line 243-246**  Compared to the body weight before the first administration, all male SD rats had a body weight of 138.4% at day 8, 173.6% at day 15, 200.4% at day 22, and 225.0% at day 29, while all female SD rats had a body weight of 132.2% at day 8, 153.1% at day 15, 173.1% at day 22, and 187.3% at day 29. |

18. Line 261-262: Delete “Findings of”. Delete “the level of” (hematology is a study not level). Change “serum IgE and histamine” to “serum IgE and histamine levels”.

- Thank you for pointing this out. We have modified the text accordingly in the revised manuscript.

| Original version | Revised version  (the revised parts are indicated in red font) |
| --- | --- |
| **Line 261-262**  Findings of the level of hematology, clinical biochemistry, and serum IgE and histamine | **Line 267-268**  Hematology, clinical biochemistry, and serum IgE and histamine levels |

19. Line 266-272: Delete “was”.

- Thank you for pointing this out. We have modified the text accordingly in the revised manuscript.

| Original version | Revised version  (the revised parts are indicated in red font) |
| --- | --- |
| **Line 266-272**  In clinical biochemistry, compared to the control group, the albumin/globulin ratio was significantly decreased in male group 6 (*p* < 0.05), the level of glucose was significantly decreased in male group 3, 5, and 6 (*p* < 0.05, respectively), and the level of sodium was significantly increased in male group 4 (*p* < 0.01) and 6 (*p* < 0.05) (Table 2).  In females, the level of total bilirubin was significantly decreased in group 2 and 4 (*p* < 0.05, respectively) and the level of chloride was significantly increased in group 2 (*p* < 0.05) and 3 (*p* < 0.01), compared to the control group (Table 3). | **Line 271-277**  In clinical biochemistry, compared to the control group, the albumin/globulin ratio significantly decreased in male group 6 (*p* < 0.05), the level of glucose significantly decreased in male group 3, 5, and 6 (*p* < 0.05, respectively), and the level of sodium significantly increased in male group 4 (*p* < 0.01) and 6 (*p* < 0.05) (Table 2).  In females, the level of total bilirubin significantly decreased in group 2 and 4 (*p* < 0.05, respectively) and the level of chloride significantly increased in group 2 (*p* < 0.05) and 3 (*p* < 0.01), compared to the control group (Table 3). |

20. Line 277: Change “increase or decrease” to “change”.

- Thank you for pointing this out. We have modified the text accordingly in the revised manuscript.

| Original version | Revised version  (the revised parts are indicated in red font) |
| --- | --- |
| **Line 276-278**  There was also no significant increase or decrease in the level of serum IgE and histamine in NZW rabbits administered polysorbate 20 and HSA compared to the control group. | **Line 282-284**  There was also no significant change in the level of serum IgE and histamine in NZW rabbits administered polysorbate 20 and HSA compared to the control group. |

21. Line 279 and 284: Delete “The level of”, and “in the current study”. Change “IgE and histamine” to “IgE and histamine levels”.

- Thank you for pointing this out. We have modified the text accordingly in the revised manuscript.

| Original version | Revised version  (the revised parts are indicated in red font) |
| --- | --- |
| **Line 279**  Table 2. The level of hematology, clinical biochemistry, and serum IgE and histamine in male Sprague-Dawley (SD) rats in the current study. | **Line 285**  Table 2. Hematology, clinical biochemistry, and serum IgE and histamine levels in male Sprague-Dawley (SD) rats. |
| **Line 284-285**  Table 3. The level of hematology, clinical biochemistry, and serum IgE and histamine in female Sprague-Dawley (SD) rats in the current study. | **Line 290**  Table 3. Hematology, clinical biochemistry, and serum IgE and histamine levels in female Sprague-Dawley (SD) rats. |

22. Line 280-282 and 286-288: Change “Data were presented as mean ± standard error of the mean” to “Data ae presented as mean ± SEM”. Delete “All statistical analyses were performed using GraphPad Prism 282 8.0 (GraphPad Software, Inc., San Diego, CA)”

- Thank you for pointing this out. We have modified the text accordingly in the revised manuscript.

| Original version | Revised version  (the revised parts are indicated in red font) |
| --- | --- |
| **Line 280-282**  Data were presented as mean ± standard error of the mean.  All data were analyzed using Kruskal-Wallis test and the significance of intergroup difference between the control and administered groups was assessed using Dunn's Rank Sum test. All statistical analyses were performed using GraphPad Prism 8.0 (GraphPad Software, Inc., San Diego, CA). | **Line 286-288**  Data are presented as mean ± SEM.  All data were analyzed using Kruskal-Wallis test and the significance of intergroup difference between the control and administered groups was assessed using Dunn's Rank Sum test. [deleted] |
| **Line 286-288**  Data were presented as mean ± standard error of the mean.  All data were analyzed using Kruskal-Wallis test and the significance of intergroup difference between the control and administered groups was assessed using Dunn's Rank Sum test. All statistical analyses were performed using GraphPad Prism 8.0 (GraphPad Software, Inc., San Diego, CA). | **Line 291-293**  Data are presented as mean ± SEM.  All data were analyzed using Kruskal-Wallis test and the significance of intergroup difference between the control and administered groups was assessed using Dunn's Rank Sum test. [deleted] |

23. Line 290: Delete “Findings of”.

- Thank you for pointing this out. We have modified the text accordingly in the revised manuscript.

| Original version | Revised version  (the revised parts are indicated in red font) |
| --- | --- |
| **Line 290**  Findings of necropsy and histopathology | **Line 295**  Necropsy and histopathology |

24. Line 371: Change “those” to “these”.

- Thank you for pointing this out. We have modified the text accordingly in the revised manuscript.

| Original version | Revised version  (the revised parts are indicated in red font) |
| --- | --- |
| **Line 371-372**  Therefore, those findings were presumed to be due to the intramuscular injection rather than any effect of polysorbate 20. | **Line 376**  Therefore, these findings were presumed to be due to the intramuscular injection rather than any effect of polysorbate 20. |

25. Line 381: Just 2% will be better instead of 2.0%.

- Thank you for pointing this out. We have modified the text accordingly in the revised manuscript.

| Original version | Revised version  (the revised parts are indicated in red font) |
| --- | --- |
| **Line 379-382**  However, despite its very low frequency, side effects in the allergy category have been reported in humans using shampoos (8.4% polysorbate 20, 2 cases among 5.88 million uses), cuticle softeners (2.0% polysorbate 20, 24 cases among 131 million use), and paste masks (2.0% polysorbate 20, 11 cases among 120.7 million uses) containing polysorbate 20 [14,15]. | **Line 384-387**  However, despite its very low frequency, side effects in the allergy category have been reported in humans using shampoos (8.4% polysorbate 20, 2 cases among 5.88 million uses), cuticle softeners (2% polysorbate 20, 24 cases among 131 million use), and paste masks (2% polysorbate 20, 11 cases among 120.7 million uses) containing polysorbate 20 [14,15]. |

26. Line 393-396: Change “hematology and clinical biochemistry” to “hematology and clinical biochemistry parameters”. Delete “of the NZW rabbits”.

- Thank you for pointing this out. We have modified the text accordingly in the revised manuscript.

| Original version | Revised version  (the revised parts are indicated in red font) |
| --- | --- |
| **Line 393-396**  There were also no toxicologically significant changes, such as changes in the body weight or the hematology and clinical biochemistry in all rabbits administered polysorbate 20 and all toxicological values in NZW rabbits were within the normal range for the same age of the NZW rabbits [41,42]. | **Line 398-401**  There were also no toxicologically significant changes, such as changes in the body weight or the hematology and clinical biochemistry parameters in all rabbits administered polysorbate 20 and all toxicological values in NZW rabbits were within the normal range for the same age [41,42]. |

27. Line 397: Change “was” to “is”.

- Thank you for pointing this out. We have modified the text accordingly in the revised manuscript.

| Original version | Revised version  (the revised parts are indicated in red font) |
| --- | --- |
| **Line 396-398**  These results of the intradermal irritation study indicated that polysorbate 20 was not an allergen in NZW rabbits, which was consistent with the results of the four-week repeated dose toxicity study in SD rats. | **Line 401-403**  These results of the intradermal irritation study indicated that polysorbate 20 is not an allergen in NZW rabbits, which was consistent with the results of the four-week repeated dose toxicity study in SD rats. |

28. Line 415: Authors maybe can wish to thank the institutions the works was carried out and the people who assisted in the work, if they wish to. Not applicable is not the appropriate term.

- Thank you for your comments. We have added thanks to colleagues who helped with the works.

| Original version | Revised version  (the revised parts are indicated in red font) |
| --- | --- |
| **Line 415**  Not applicable | **Line 420-421**  The authors thanks Do Yeon Lee and Min-Seo Choi, colleagues at the Medytox, Inc., for their helpful discussion and careful reading of the manuscript. |

29. Line 466: Please check the journal abbreviation.

- Thank you for pointing this out. We have modified the text accordingly in the revised manuscript.

| Original version | Revised version  (the revised parts are indicated in red font) |
| --- | --- |
| **Line 464-466**  Diehl K, Hull R, Morton D, Pfister R, Rabemampianina Y, Smith D, et al. A good practice guide to the administration of substances and removal of blood, including routes and volumes. J Appl Toxicol An Int J. 2001;21: 15–23. | **Line 470-472**  Diehl K, Hull R, Morton D, Pfister R, Rabemampianina Y, Smith D, et al. A good practice guide to the administration of substances and removal of blood, including routes and volumes. J Appl Toxicol. 2001;21: 15–23. |

30. Line 490 and 519: Please check the journal abbreviation (use standard abbreviations and check LTWA/ISSN website).

- Thank you for your comments. According to the PLOS ONE's submission guidelines, the following is stated in relation to the reference format:
- “PLOS uses the reference style outlined by the International Committee of Medical Journal Editors (ICMJE). Journal name abbreviations should be those found in the National Center for Biotechnology Information (NCBI) databases.”
- We have checked journal name abbreviations (NCBI database) and kept the existing reference citation form.

**[Reviewer #2]**

1. If the authors have adequately addressed your comments raised in a previous round of review and you feel that this manuscript is now acceptable for publication, you may indicate that here to bypass the “Comments to the Author” section, enter your conflict of interest statement in the “Confidential to Editor” section, and submit your "Accept" recommendation: **All comments have been addressed.**
2. Is the manuscript technically sound, and do the data support the conclusions?**: Yes**
3. Has the statistical analysis been performed appropriately and rigorously?**: Yes**
4. Have the authors made all data underlying the findings in their manuscript fully available?**: Yes**
5. Is the manuscript presented in an intelligible fashion and written in standard English?**: Yes**
6. Review Comments to the Author: **No response**

- Thank you for the encouraging feedback, including previous reviews.
